# Biogeographic and disease-specific alterations in epidermal lipid composition and single-cell analysis of acral keratinocytes

**DOI:** 10.1172/jci.insight.159762

**Published:** 2022-08-22

**Authors:** Alexander A. Merleev, Stephanie T. Le, Claire Alexanian, Atrin Toussi, Yixuan Xie, Alina I. Marusina, Steven M. Watkins, Forum Patel, Allison C. Billi, Julie Wiedemann, Yoshihiro Izumiya, Ashish Kumar, Ranjitha Uppala, J. Michelle Kahlenberg, Fu-Tong Liu, Iannis E. Adamopoulos, Elizabeth A. Wang, Chelsea Ma, Michelle Y. Cheng, Halani Xiong, Amanda Kirane, Guillaume Luxardi, Bogi Andersen, Lam C. Tsoi, Carlito B. Lebrilla, Johann E. Gudjonsson, Emanual Maverakis

**Affiliations:** 1 Department of Dermatology, University of California, Davis, Sacramento, California, USA.; 2 Department of Chemistry, University of California, Davis, Davis, California, USA.; 3 Verso Biosciences, Davis, California, USA.; 4 Department of Dermatology, University of Michigan, Ann Arbor, Michigan, USA.; 5 Department of Dermatology, University of California Irvine, Irvine, California, USA.; 6 Department of Internal Medicine, Division of Rheumatology, University of Michigan, Ann Arbor, Michigan, USA.; 7 Department of Rheumatology and; 8 Department of Surgery, University of California, Davis, Sacramento, California, USA.; 9 Department of Computational Medicine and Bioinformatics, Department of Biostatistics, Center for Statistical Genetics, University of Michigan, Ann Arbor, Michigan, USA.; 10 Department of Biochemistry and Molecular Medicine and; 11 Foods for Health Institute, University of California, Davis, Davis, California, USA.

**Keywords:** Dermatology, Skin

## Abstract

The epidermis is the outermost layer of skin. Here, we used targeted lipid profiling to characterize the biogeographic alterations of human epidermal lipids across 12 anatomically distinct body sites, and we used single-cell RNA-Seq to compare keratinocyte gene expression at acral and nonacral sites. We demonstrate that acral skin has low expression of EOS acyl-ceramides and the genes involved in their synthesis, as well as low expression of genes involved in filaggrin and keratin citrullination (*PADI1* and *PADI3*) and corneodesmosome degradation, changes that are consistent with increased corneocyte retention. Several overarching principles governing epidermal lipid expression were also noted. For example, there was a strong negative correlation between the expression of 18-carbon and 22-carbon sphingoid base ceramides. Disease-specific alterations in epidermal lipid gene expression and their corresponding alterations to the epidermal lipidome were characterized. Lipid biomarkers with diagnostic utility for inflammatory and precancerous conditions were identified, and a 2-analyte diagnostic model of psoriasis was constructed using a step-forward algorithm. Finally, gene coexpression analysis revealed a strong connection between lipid and immune gene expression. This work highlights (a) mechanisms by which the epidermis is uniquely adapted for the specific environmental insults encountered at different body surfaces and (b) how inflammation-associated alterations in gene expression affect the epidermal lipidome.

## Introduction

The stratum corneum (SC) is the outermost layer of the epidermis. It is composed of terminally differentiated and enucleated keratinocytes that reside within a lipid matrix, like bricks and mortar. The lipid matrix is a heterogenous mixture of free fatty acids (FAs), cholesterol, and ceramides (Supplemental Figure 1; supplemental material available online with this article; https://doi.org/10.1172/jci.insight.159762DS1). Combined, these lipids form a permeability barrier that limits transepidermal water loss (1, 2), prevents penetration of environmental substances by diffusion (3), halts electrolytes from escaping through the skin, and serves as a major impediment to microorganism invasion (4). However, in addition to their structural functions, SC lipids also initiate signal transduction events that, among other things, facilitate keratinocyte differentiation and innate and adaptive immune cell activation (5, 6).

Although the skin transcriptome and SC lipids have been extensively studied in health and disease (7–11), little is known about how the skin is geographically tuned to meet the unique environmental challenges encountered at the different anatomical surfaces of the human body. In a broad sense, we know that the skin covering the heel of the foot is thick and rigid to resist physical trauma, while the skin of the face is thin and flexible to accommodate for the dynamics of facial expression. But, with the exception of a few studies demonstrating regional alterations in the skin transcriptome, usually in the setting of skin disease, transcriptomic and lipidomic research on skin biogeography has been limited (11–13). Here, we identify the biogeographical alterations of the keratinocyte transcriptome at the single-cell level that yield site-specific structural changes in the epidermis, including specific geographic and disease-associated alterations to the epidermal lipidome.

Commonly, pathogenic alterations to the epidermis manifest clinically as scale, which forms on the surface of the skin when the SC exfoliates abnormally. Pertinent to the current study is the fact that many dermatologic conditions have unique-appearing scale, which we hypothesize is due to unique alterations of the epidermal lipidome. For example, psoriasis is a chronic immune-mediated skin disease that is characterized by well-demarcated erythematous plaques with overlying micaceous scale (14). Previous lipid profiling studies have demonstrated that patients with psoriasis have disease-associated alterations of their epidermal ceramides (8–10) and ceramide intermediates (15). Additional epidermal lipid alterations have also been noted in patients with atopic dermatitis (AD). However, to date, diagnostic lipid biomarkers have not been identified for any of these diseases, and previous reports of disease-associated lipid alterations have generally focused on differential expression of ceramide classes, rather than specific structural changes linked to alterations in lipid gene expression.

Here, we (a) identify transcriptional alterations that likely account for the site-specific structural changes of the human epidermis; (b) construct a detailed biogeographic map of epidermal lipid expression and make predictions as to how compositional changes to epidermal lipids can alter the structure of the SC; (c) uncover the general rules governing correlative relationships among different lipid structures; (d) identify potentially novel clusters of coregulated lipid genes; (e) link lipid gene expression to the expression of autoinflammatory mediators, especially *IL36B*; (f) characterize a variety of disease-specific alterations in epidermal lipid expression; (g) identify and validate disease-specific diagnostic lipid biomarkers; and (h) demonstrate how disease-associated cytokines can alter lipid gene expression, likely resulting in the characteristic changes in epidermal lipid expression that are associated with the same cytokines.

## Results

### Biogeography of epidermal lipids.

**Figure 1:**
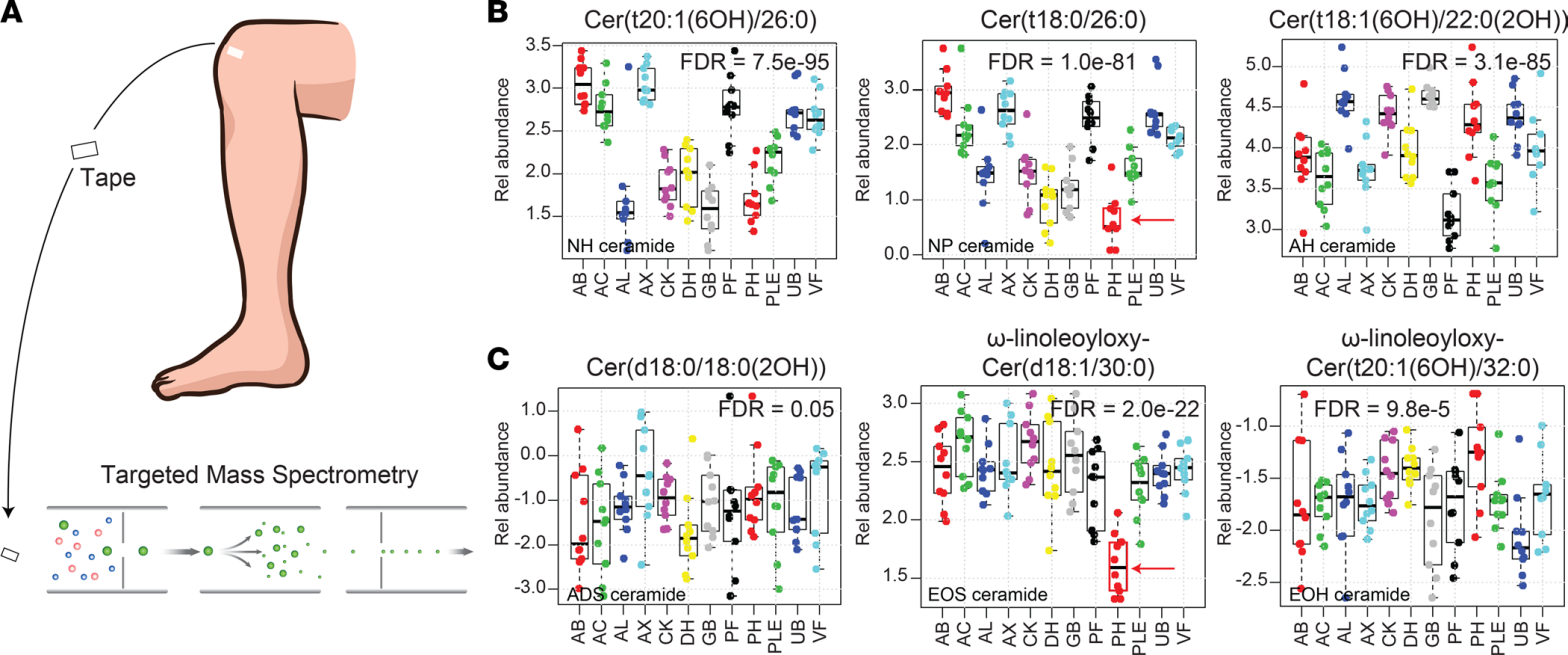
Biogeographical variation of epidermal ceramides. (**A**) Analysis of tape-stripping samples by targeted mass spectrometry. (**B**) The relative abundance of 351 lipids was simultaneously monitored across 12 different anatomic locations; representative ceramides are shown. Sites monitored included abdomen (AB), antecubital fossa (AC), alar crease (AL), axillae (AX), cheek (CK), dorsal surface of the hand (DH), glabella (GB), popliteal fossa (PF), plantar heel (PH), anterior proximal lower extremity (PLE), upper back (UB), and volar forearm (VF). Results are presented as box-and-whisker plots, where the upper and lower bars connected to each box indicate the boundaries of the normal distribution, and the box edges mark the first and third quartile boundaries within each distribution. The dark horizontal line represents the median. The relative abundances of the NH ceramide Cer(t20:1[6OH]/26:0), the NP ceramide Cer(t18:0/26:0), and the AH ceramide Cer(t18:1[6OH]/22:0[2OH]) were highly variable across different anatomic locations. (**C**) In contrast, the expression of the ADS dihydroxyceramide Cer(d18:0/18:0[2OH]) was largely invariable across all anatomic sites, a finding representative of all ADS ceramides monitored (Supplemental Table 1). Acyl-ceramides EOS ω-linoleoyloxy-Cer(d18:1/30:0) and EOH ω-linoleoyloxy-Cer(t20:1[6OH]/32:0) are also shown. Note the low expression of the EOS ceramide in PH epidermis, which was a typical finding for EOS ceramide expression (Supplemental Table 1).

To characterize the natural biogeography of epidermal lipid expression, tape stripping samples were taken from 10 adults, balanced for age and sex (median age, 26 ± 3 years), with no underlying skin or major medical conditions. Each individual had 12 different anatomic sites sampled (abdomen [AB], antecubital fossa [AC], alar crease [AL], axillae [AX], cheek [CK], dorsal surface of hand [DH], glabella [GB], popliteal fossa [PF], plantar heel [PH], anterior proximal lower extremity [PLE], upper back [UB], and volar forearm [VF]). A targeted mass spectrometry approach (Figure 1A) was then employed to simultaneously quantify the abundance of 350 epidermal lipids within each sample (Supplemental Table 1). The subclass structures of these lipids are shown in Supplemental Figure 1. A mixed effects model revealed that 272 of the monitored lipids had anatomic site specificity (FDR < 0.05) (Supplemental Table 2). Representative ceramides are shown in Figure 1B. Among the monitored lipids, the 6-hydroxyceramides Cer(t20:1[6OH]/26:0) and Cer(t18:1[6OH]/22:0[2OH]), and the phytoceramide Cer(t18:0/26:0) had the strongest differential expression (FDR = 7.5 × 10^–95^, 3.1 × 10^–85^, and 1.0 × 10^–81^, respectively). Specifically, the expression of the NH ceramide Cer(t20:1[6OH]/26:0) was relatively high within the epidermis of the AB, AC, AX, and PF and relatively low within the epidermis of the AL, CK, DH, GB, and PH. In contrast, the AH ceramide Cer(t18:1[6OH]/22:0[2OH]) had the opposite expression pattern, with relative high expression in the epidermis of AL, CK, GB, and PH (Figure 1B). These lipid expression patterns suggest that some anatomical sites are more closely related in terms of their lipid composition. However, unlike NH, NP, and AH ceramides, the abundance of ADS ceramides was very similar across the different anatomic locations; ADS ceramide Cer(d18:0/18:0[2OH]) is shown here as an example (Figure 1C). The lack of similarity between PH and other body sites was also noteworthy (Figure 1, B and C, red arrows).

**Figure 2:**
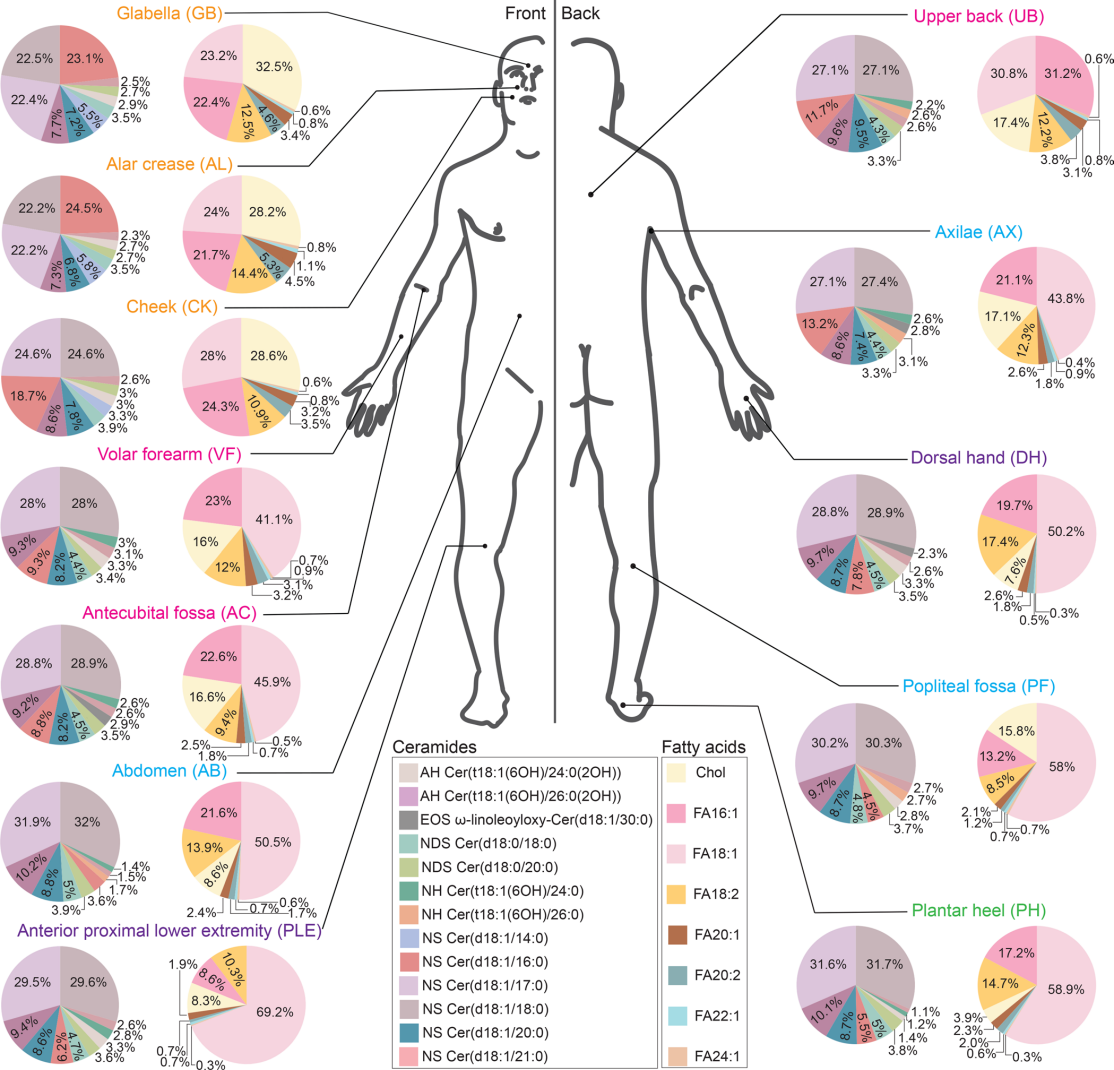
Biogeography of epidermal lipids across 12 anatomical sites. Anatomical sites used for skin adhesive tape sampling are shown. Two pie charts are shown for each anatomical location, one containing ceramides (left) and the other cholesterol and unsaturated fatty acids (right). For each pie chart, 100% equals the combined abundance of listed lipids. Lipids included in the pie charts are color coded and listed in the key. Across different sites, some pie charts look similar to one another (e.g., GB, AL, and CK), indicating similarities in lipid composition.

Next, to better visualize the biogeographic variation in epidermal lipid expression, we constructed (a) pie charts from representative highly abundant lipids and (b) a cluster dendrogram to organize anatomic sites by their lipid similarities. For each location, 2 pie charts were constructed: one for representative ceramides and the second for common unsaturated FAs and cholesterol, where 100% equals the combined total for the listed lipids (not all epidermal lipids). Lipids selected for inclusion in the pie charts had (a) variable expression by anatomic location and (b) were abundant enough to be visualized in the pie chart after exclusion of saturated FAs. This analysis revealed that GB, AL, and CK had a high relative abundance of cholesterol sulfate (Chol-SO4) (Figure 2). In contrast, PH had a low relative abundance of Chol-SO4 but a high relative abundance of the FA 18:1. The UB was unique in that FA 16:1 was the most common unsaturated FA (Figure 2). In terms of ceramides, GB and AL had a high abundance of NS ceramide Cer(d18:1/16:0) (>20%, relative to other displayed ceramides), while NS ceramides Cer(d18:1/17:0) and Cer(d18:1/18:0) were more abundant at other anatomic sites, especially AB, PH, and PF (Figure 2).

**Figure 3:**
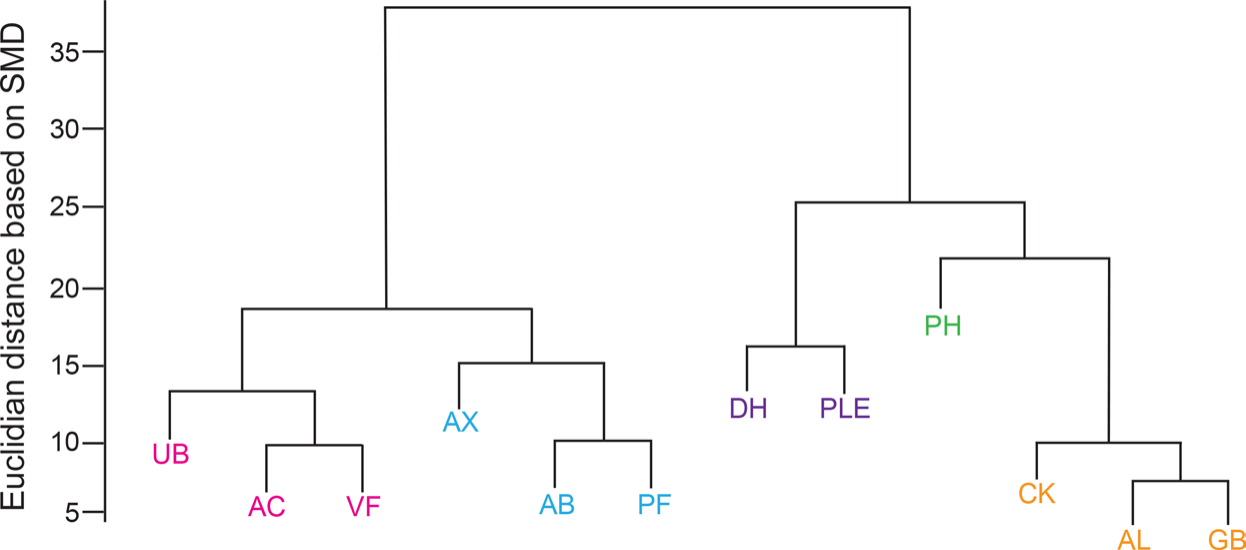
Clustering of different anatomical surfaces by similarities in epidermal lipid composition. A cluster dendrogram was constructed to assess similarities in epidermal lipid composition between different anatomical surfaces. Anatomic locations that cluster together are depicted in the same color. The height of the link connecting different anatomical locations represents how similar their respective epidermal lipid compositions are. Note that PH does not cluster well with other anatomic sites.

**Figure 4:**
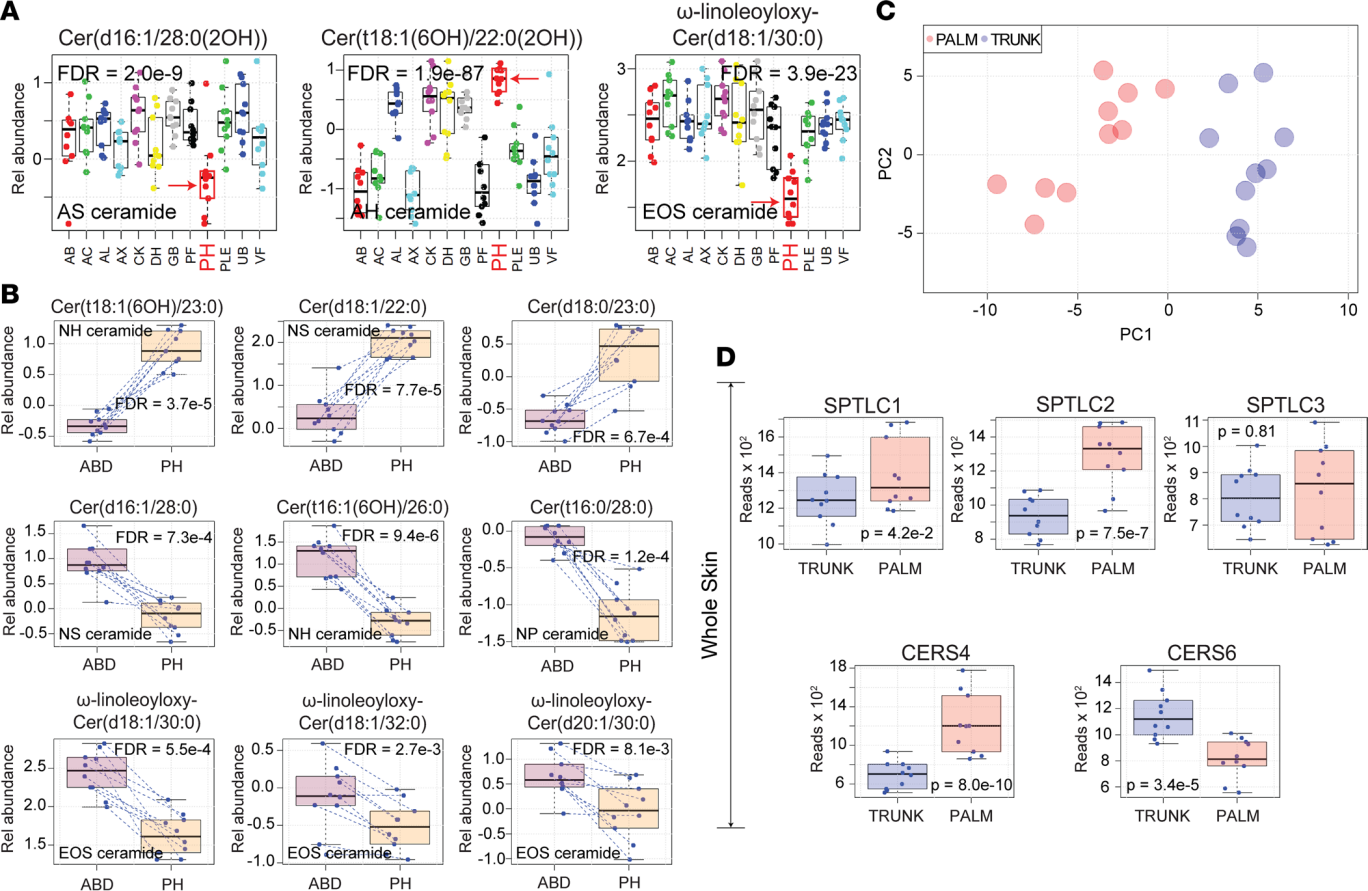
Palmoplantar skin has a unique epidermal lipid composition. (**A**) Results are displayed as box-and-whisker plots, as described in Figure 1. Representative lipids were chosen to highlight the differences in lipid composition between PH (red arrow) and other anatomic locations. Note the low abundance of AS ceramide Cer(d16:1/28:0[2OH]) in PH skin and the increased abundance of AH ceramide Cer(t18:1[6OH]/22:0[2OH]). This pattern was also observed for all monitored ceramides with similar length sphingoid and fatty acid moieties (Supplemental Figure 2 and Supplemental Table 1). The acyl-ceramide EOSω-linoleoyloxy-Cer(d18:1/30:0) was also decreased in PH epidermis. (**B**) Additional examples of differentially expressed ceramides in PH skin that follow the same trends as presented in **A**. (**C**) Principal component analysis of lipid-associated metabolic gene expression data revealed complete separation of samples by anatomic location. (**D**) Whole-tissue RNA-Seq performed on palm and trunk biopsy specimens identifies differentially expressed lipid-associated metabolic genes. Genes presented here are relevant to the synthesis of the differentially expressed ceramides in PH epidermis presented in **A** and **B**.

When lipid similarities were presented hierarchically as a cluster dendrogram, the high degree of similarity between CK, AL, and GB; UB, AC, and VF; AX, AB, and PF; and DH and PLE was clearly evident (Figure 3). Of these, the CK, AL, and GB cluster appeared to be most similar to one another. PH failed to colocalize with other anatomic sites. The unique lipid profile of the PH epidermis was also evident in the box-and-whisker plots of the individually monitored ceramides (Figure 4, A and B and Supplemental Figure 2, red arrows). Specifically, ceramides composed of an 18-carbon sphingoid base and a FA moiety 20, 22, or 23 carbons in length were uniformly increased in PH skin (Figure 4B and Supplemental Figure 2B). PH skin was also hallmarked by the relative low expression of 16-carbon sphingoid base ceramides with long-chain FAs (Figure 4B). Another striking finding was the strong downregulation of the acyl-ceramide ω-linoleoyloxy-Cer(d18:1/30:0) in PH skin (Figure 4, A and B).

### Transcriptome alterations in lipid-associated genes.

To investigate how epidermal ceramides of acral skin were synthesized, we next performed RNA-Seq to search for differential expression in lipid-associated metabolic genes that could account for the noted differences in the epidermal ceramides. Differential gene expression analysis demonstrated that 45 out of a set of 83 genes (54%) related to lipid metabolism were significantly differentially expressed in palm versus trunk skin (FDR < 0.05) (Supplemental Table 3 and Figure 4D). A principal component analysis (PCA) of lipid-associated metabolic gene expression data revealed complete separation of samples by anatomic location (Figure 4C).

**Figure 5:**
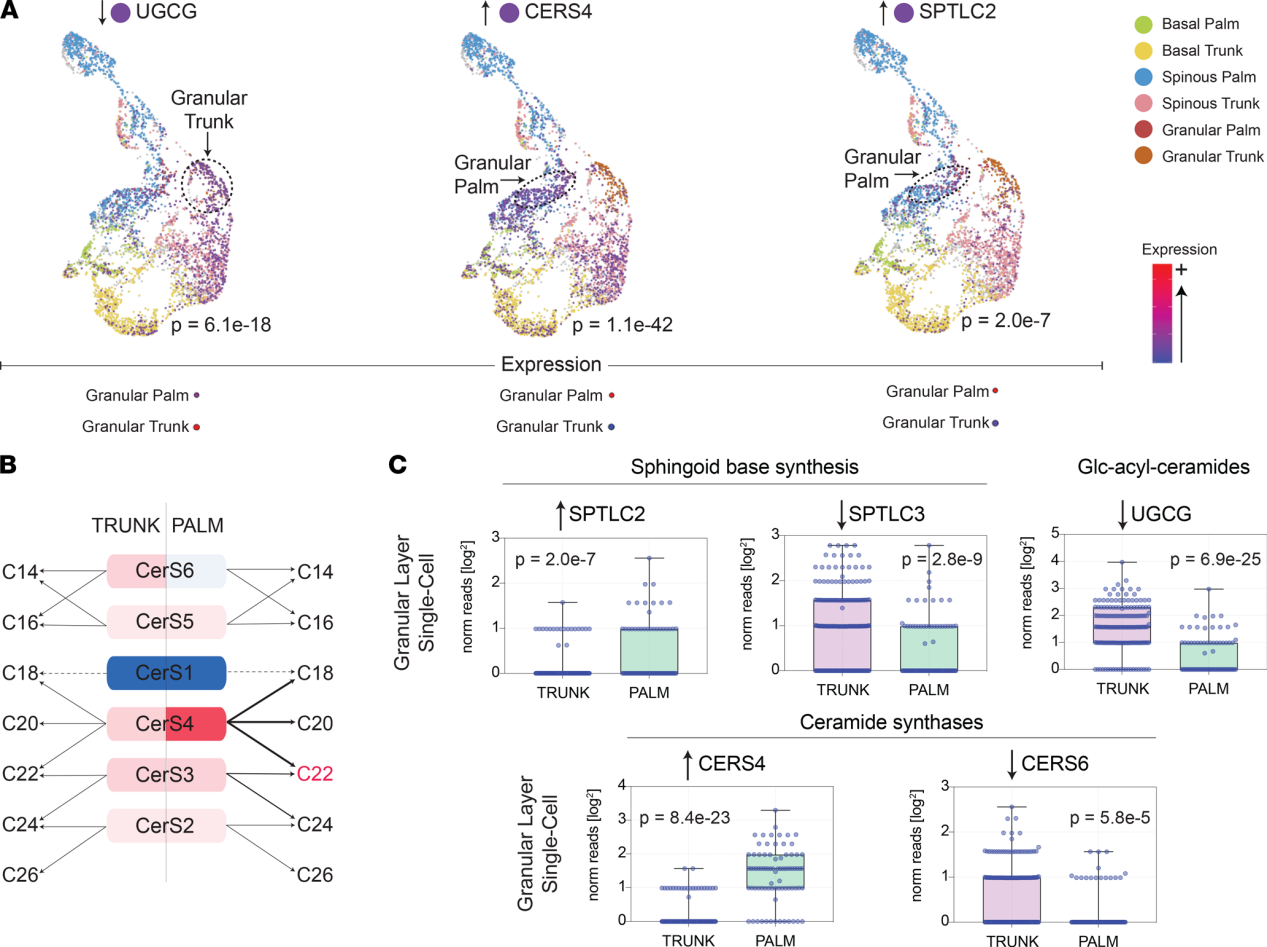
Differential expression of lipid-associated metabolic genes assessed by single-cell sequencing. (**A**) Individual keratinocyte transcriptome data are presented using the uniform manifold approximation and projection (UMAP) method. Each dot represents an individual keratinocyte. Note that keratinocytes originating from the same anatomic location and epidermal layer cluster together (basal [palm, green; trunk, yellow], spinous [palm, blue; trunk, pink], and granular [palm, red; trunk, orange] layers). Cells expressing the noted lipid-associated are depicted in purple. Dashed circles are drawn around the keratinocyte population, in which the expression of the noted gene is most strongly upregulated. (**B**) Predicted alterations in ceramide lipid expression based on ceramide synthase gene expression in trunk versus palm skin. (**C**) Single-cell RNA-Seq data of palm and trunk epidermal granular layer keratinocytes presented as box-and-whisker plots. Genes selected for presentation are relevant to the observed lipid alterations in PH skin. Each individual data point represents the number of reads that mapped to the indicated gene in a unique granular layer keratinocyte.

We next conducted single-cell RNA-Seq of trunk and palm epidermis (Figure 5, A and C). This analysis revealed that differential expression of lipid-associated metabolic genes is highly dependent on the type of keratinocyte analyzed. For example, in acral skin, *SPTLC2* is significantly upregulated only in granular layer keratinocytes and not in spinous or basal layer keratinocytes. In contrast, *CERS4* expression is increased across all keratinocyte layers in acral skin. Diagrams of how these alterations in lipid-associated genes should alter epidermal PH ceramide expression are presented in Figure 5B and Supplemental Figure 3. Also, the acyl-ceramide–associated gene, *ABHD5*, was strongly downregulated in acral skin (Supplemental Figure 4B), which coincides with the low expression of the acyl-ceramide ω-linoleoyloxy-Cer(d18:1/30:0) in PH skin (Figure 1C). One issue with these results is that single-cell RNA-Seq was performed on acral hand skin and the targeted lipid profiling was performed on acral foot skin. Thus, to ensure consistency across these acral surfaces, a subset of patients underwent acral foot biopsies for bulk RNA-Seq analysis, which revealed that the key genes discussed above were also differentially expressed in acral foot skin (Supplemental Figure 4C).

### Transcriptome alterations are likely to yield the compact orthokeratosis of acral skin.

**Figure 6:**
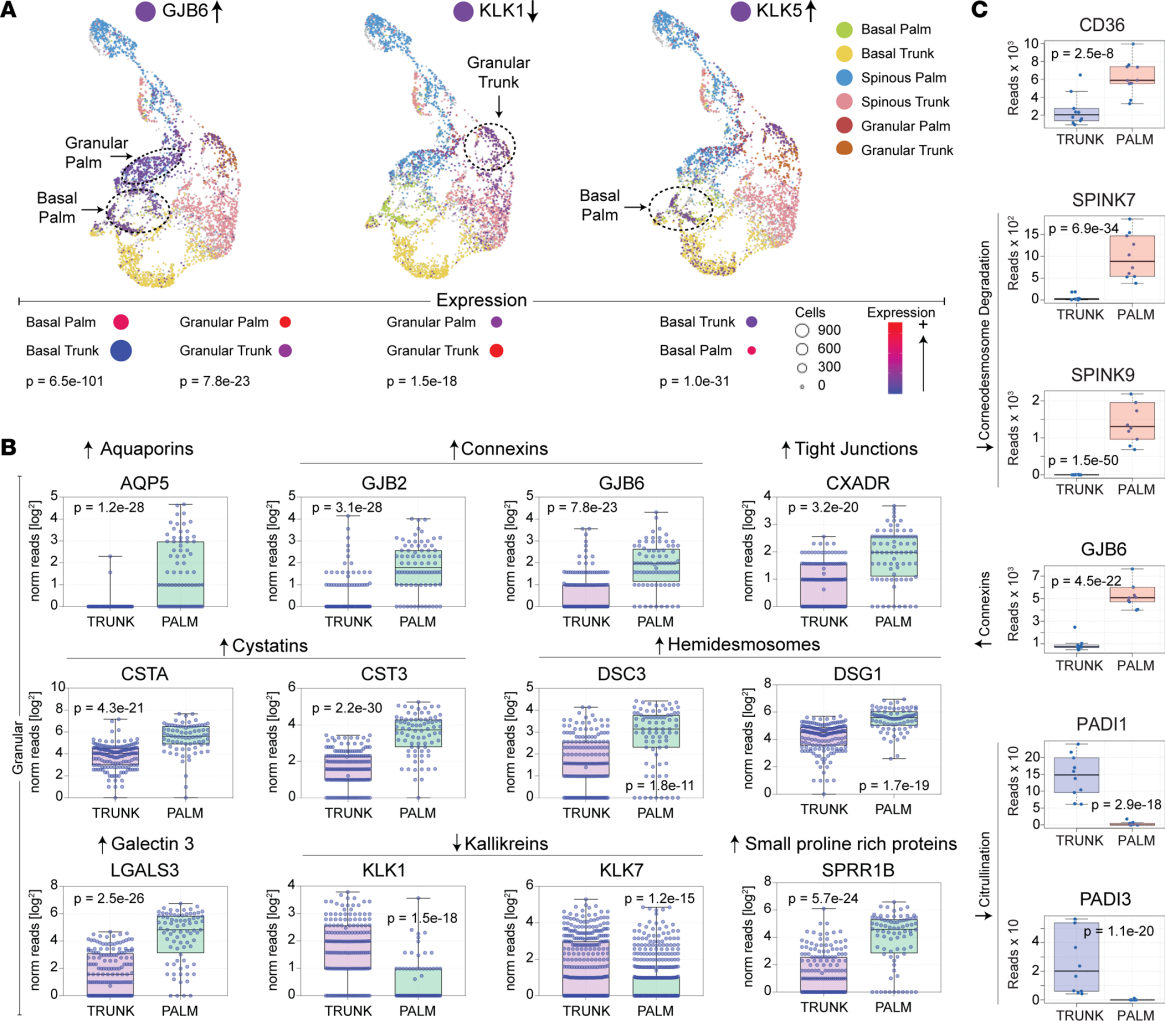
Transcriptome alterations in acral keratinocytes provide insight into the distinctive epidermal features of palmoplantar skin. (**A**) UMAP dimensionality reduction plots of single-cell RNA-Seq data. Single-cell RNA-Seq was performed on keratinocytes isolated from paired palm and trunk skin biopsies. Each dot represents an individual keratinocyte (basal [palm, green; trunk, yellow]; spinous [palm, blue; trunk, pink]; and granular [palm, red; trunk, orange] layers). Cells expressing the noted gene of interest are depicted in purple. Dashed circles are drawn around the keratinocyte population in which the expression of the noted gene is most strongly upregulated. Arrows to the left of gene names represents the directionality of gene expression in acral keratinocytes. (**B**) Single-cell RNA-Seq data presented as box-and-whisker plots. Each individual data point represents the number of reads that mapped to the indicated gene in a single granular layer keratinocyte. (**C**) Paired biopsies obtained from palm and trunk skin were evaluated by RNA-Seq. Results of select genes relevant to keratin citrullination and corneodesmosome degradation are presented as box-and-whisker plots.

For corneocyte desquamation to occur, corneocyte inter-corneocyte connections (corneodesmosomes) need to be degraded. Desmosomes hold together keratinocytes in the epidermis. At the outermost layer of the stratum granulosum, corneodesmosomes are formed from the addition of corneodesmosin to the extracellular portion of the desmosomes. The resulting electron-dense structures are integrated into the cornified cell envelopes. Desquamation occurs when the corneodesmosomes are fully degraded. Since acral skin is hallmarked by corneocyte retention, we searched for alterations in the acral skin transcriptome that would delay corneocyte desquamation. Single-cell RNA-Seq revealed that acral skin granular layer keratinocytes have low expression of corneodesmosome-degrading *KLK1* and *KLK7* (Figure 6, A and B). In contrast, the granular layer expression of *KLK5* was not downregulated (Figure 6A), but the KLK5-specific inhibitor, *SPINK9*, was highly overexpressed in palm skin (Figure 6C). Also, the cysteine protease inhibitors, *CSTA* and *CST3*, were strongly upregulated in granular layer acral keratinocytes (Figure 6B), while the filaggrin and keratin citrullination enzymes, *PADI1* and *PADI3*, were strongly downregulated (Figure 6C). Numerous other interesting patterns of gene expression in acral keratinocytes were also noted, including upregulation of genes encoding hemidesmosomes, connexins, tight junctions, cornified envelope proteins, CD36, and galectin-3 (Figure 6 and Supplemental Figure 5). These alterations are summarized in Supplemental Figure 6.

### Keratin alterations in acral skin.

**Figure 7:**
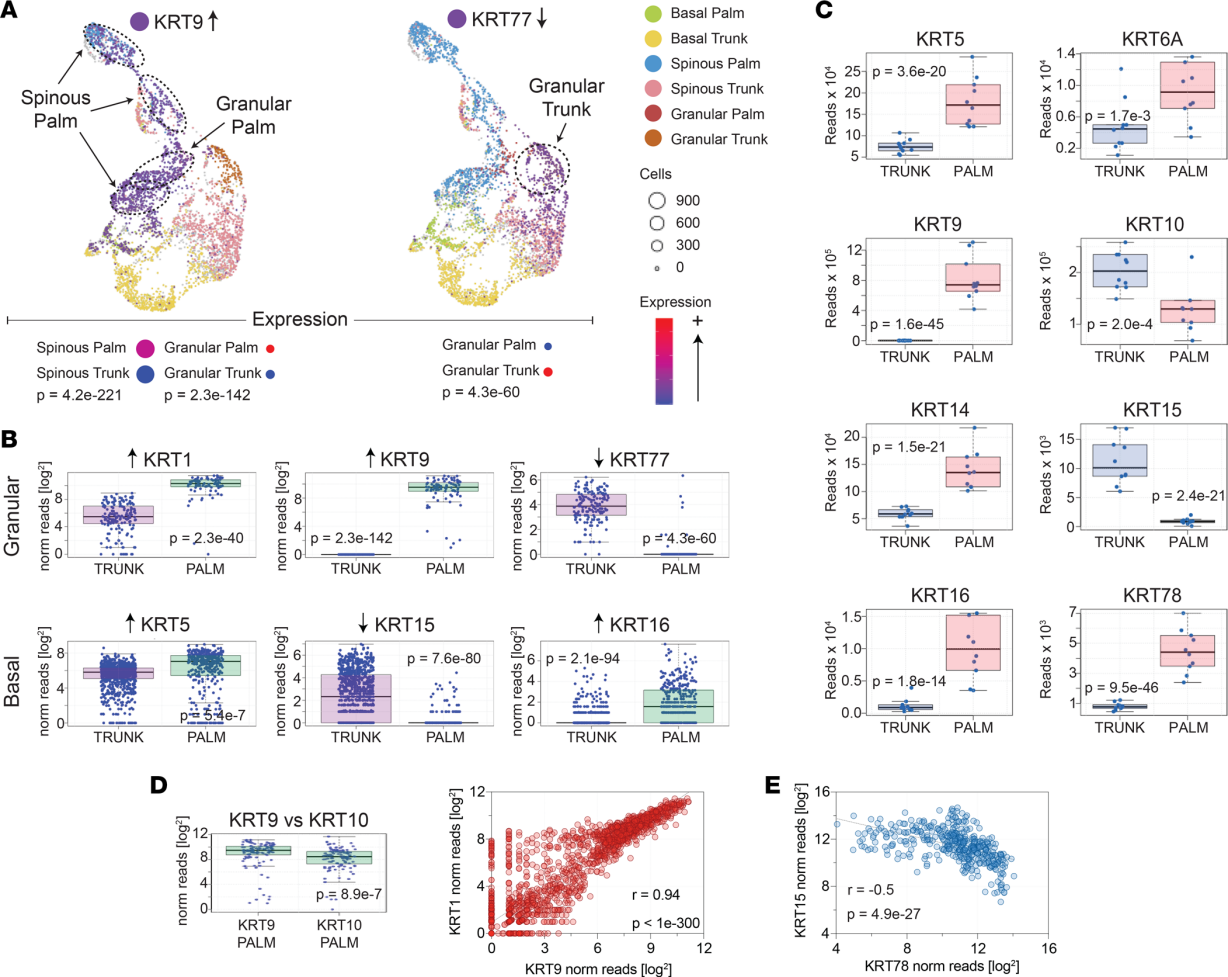
Acral skin is associated with altered expression of keratin genes. (**A**) UMAP dimensionality reduction plots of single-cell RNA-Seq data. Single-cell RNA-Seq was performed on keratinocytes isolated from paired palm and trunk skin biopsies as described in Figure 6. (**B**) Basal and granular layer single-cell keratin expression. Each individual data point represents the number of reads that mapped to the indicated gene in a single basal or granular layer keratinocyte. (**C**) RNA-Seq was performed on whole-skin biopsies obtained from palm and trunk. Box-and-whisker plots of representative keratin genes differentially expressed in palm skin are shown. (**D**) Box-and-whisker plot of *KRT9* and *KRT10* expression in palm skin. Scatter plot of *KRT9* versus *KRT1* expression in individual granular layer palm keratinocytes. Each dot represents a single granular layer keratinocyte. Note the strong correlation between *KRT1* and *KRT9* within each cell. (**E**) Correlation scatter plot of *KRT15* and *KRT78* in cultured keratinocytes, where each data point represents a different primary keratinocyte culture.

Whole tissue RNA-Seq demonstrated a clear downregulation of *KRT10* in acral skin (Figure 7C); however, the expression of its usual binding partner, *KRT1*, was strongly upregulated (Figure 7B). Interestingly, within individual keratinocytes, the increased expression of *KRT1* strongly correlated with the upregulation of *KRT9* (*r* = 0.94, *P <* 1 × 10^–300^) (Figure 7D). Furthermore, the expression of *KRT9* in single acral granular layer keratinocytes was 1.7 times that of *KRT10* (Figure 7D). Thus, in acral granular layer keratinocytes, the dominant keratin pair is likely *KRT1*/*KRT9* rather than the well-characterized *KRT1*/*KRT10* pair that is dominant at nonacral sites. It is therefore not surprising that the pathology of *KRT9* deficiency is limited to acral surfaces, such as in the case of palmoplantar keratoderma (16, 17). Single-cell RNA-Seq and whole-tissue RNA-Seq also identified other keratins upregulated in acral skin including *KRT6A*, *KRT16*, and *KRT78* (Figure 7C and Supplemental Figure 5, B and C). Downregulated keratins in acral skin included the basal layer–specific *KRT15* and the granular layer–specific *KRT77* (Figure 7B). Finally, a reciprocal relationship between *KRT78* and *KRT15* was evident in primary human keratinocyte cell lines (Figure 7E). Other differentially expressed keratins are shown in Supplemental Figure 5, B and C.

### Disease-associated lipid alterations.

Diseases such as psoriasis, AD, actinic keratoses (AK), seborrheic keratoses (SK), and tinea corporis (TI) can be diagnosed by their clinical appearance, especially by their nearly pathognomonic epidermal changes. We, thus, hypothesized that the characteristic epidermal features (i.e., unique types of scale) associated with these diseases should have corresponding molecular signatures in the epidermal lipidome. To investigate this possibility, we performed targeted mass spectrometry to profile epidermal lipids in lesional psoriasis skin (PP) (*n =* 37); nonlesional psoriasis (PN) (*n =* 16); lesional AD (*n =* 10); nonlesional atopic dermatitis (AN) (*n =* 9); AK (*n =* 10); SK (*n =* 9), TI (*n =* 3); and healthy control (NN) (*n =* 20) skin (Supplemental Table 4).

**Figure 8:**
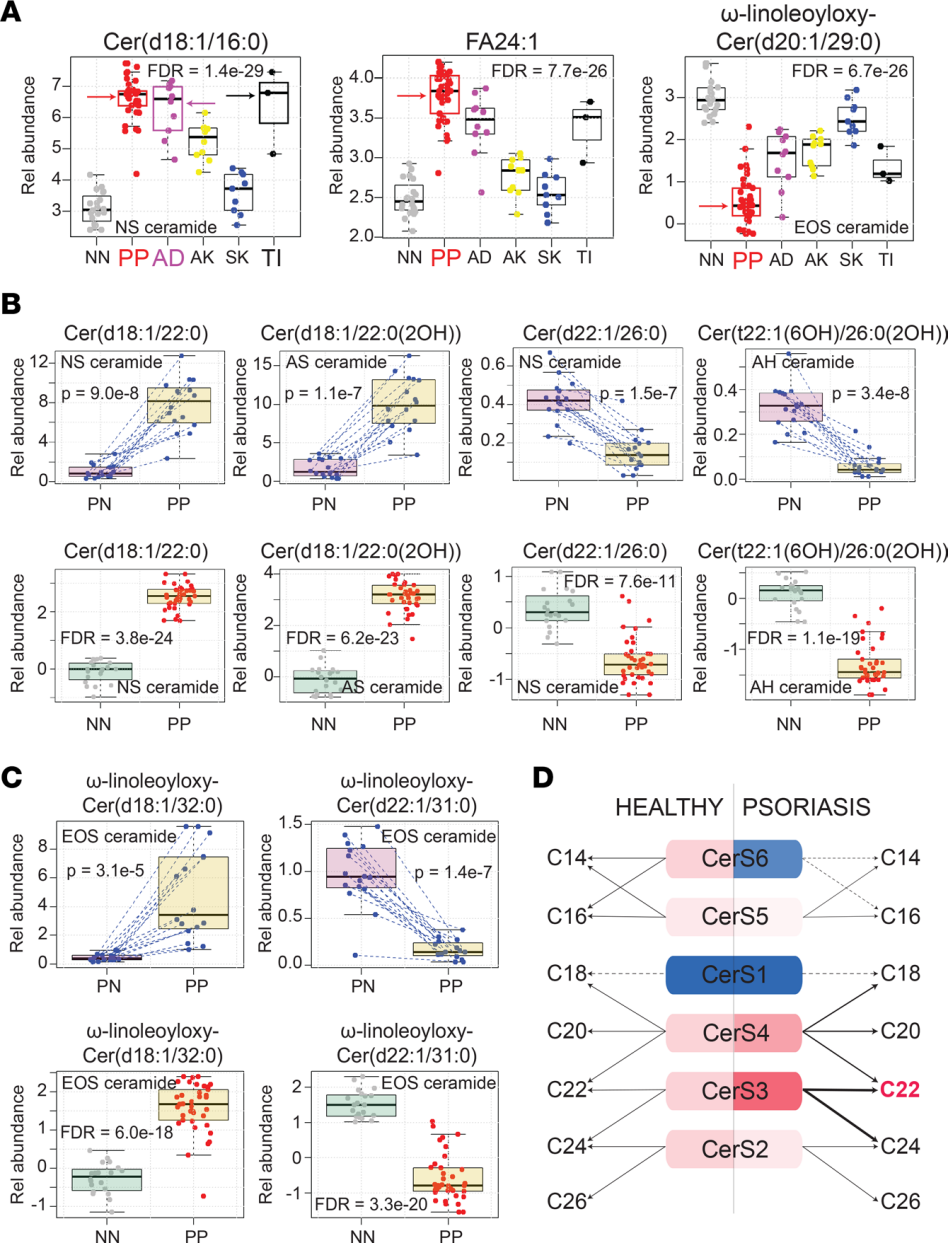
Lipid alterations in psoriasis skin. (**A**) Abundance of epidermal lipids in different dermatologic diseases as determined by targeted mass spectrometry. Results are displayed as box-and-whisker plots (as described in Figure 1). Representative lipids were chosen to highlight the characteristic patterns of lipid expression in lesional psoriasis epidermis. Red, purple, and black arrows highlight the upregulation of NS ceramide Cer(d18:1/16:0) (upper left corner) in psoriasis, atopic dermatitis, and tinea corporis lesional epidermis, respectively. NS ceramide Cer(d18:1/16:0) is an example of how certain ceramides with 18-carbon sphingoid bases are upregulated in inflammatory skin. FA 24:1 illustrates the general upregulation of unsaturated fatty acids in psoriasis, and EOS ceramide ω-linoleoyloxy-Cer(d20:1/29:0) is an example of a differentially expressed EOS ceramide. (**B**) Additional examples illustrating the trends in ceramide expression in psoriasis lesional epidermis. Upper row presents lipid expression data from paired psoriasis lesional and nonlesional samples. Lower row presents data for psoriasis versus healthy controls. (Results for additional monitored ceramides demonstrating these same trends can be found in Supplemental Figure 7, and results for all monitored lipids can be found in Supplemental Table 5.) (**C**) Differential expression of EOS ceramides in psoriasis depends in part on the length of their sphingoid base. (**D**) Predicted alterations in ceramide synthesis in psoriasis skin based upon psoriasis-associated alterations in lipid-gene expression.

This revealed a significant upregulation of the NS ceramide Cer(d18:1/16:0) in lesional skin of psoriasis, AD, and TI (FDR = 1.7 × 10^–25^; Figure 8A). Several other general observations were also apparent. For example, 18-carbon sphingoid base ceramides with FA moieties of approximately 22 carbons in length were strongly upregulated in lesional psoriasis skin (Figure 8, B and D, and Supplemental Figure 7A). In contrast, 22-carbon sphingoid base ceramides with FA moieties 26 carbons in length were strongly downregulated (Figure 8B and Supplemental Figure 7B). These general rules held true across all ceramide subclasses. Thus, rather than a particular ceramide class being up- or downregulated in psoriasis, there are specific structural features that can predict the ceramide’s expression pattern; and within each ceramide class, there will be some members that are upregulated and others downregulated. For example, when compared with healthy control or paired nonlesional skin, acyl-ceramide EOS ω-linoleoyloxy-Cer(d18:1/32:0) was strongly upregulated and EOS ω-linoleoyloxy-Cer(d22:1/30:0) was strongly downregulated in psoriasis (Figure 8C and Supplemental Figure 7C). In total, analysis of lesional versus paired nonlesional psoriasis samples identified 288 lipids that were differentially expressed (Supplemental Table 5). Finally, when compared with all other diagnostic groups combined, there were 301 lipids differentially expressed in psoriatic skin (FDR < 0.05, Supplemental Table 6). Of these, NH ceramide Cer(t18:1[6OH]/30:0), NS ceramide Cer(d18:1/30:0), FA 24:1, and EOS ceramide ω-linoleoyloxy-Cer(d20:1/29:0) were among the most differentially expressed (FDR = 1.6 × 10^–22^, 1.6 × 10^–23^, 7.7 × 10^–26^, 6.7 × 10^–26^, respectively) (Figure 8A and Supplemental Table 6).

**Figure 9:**
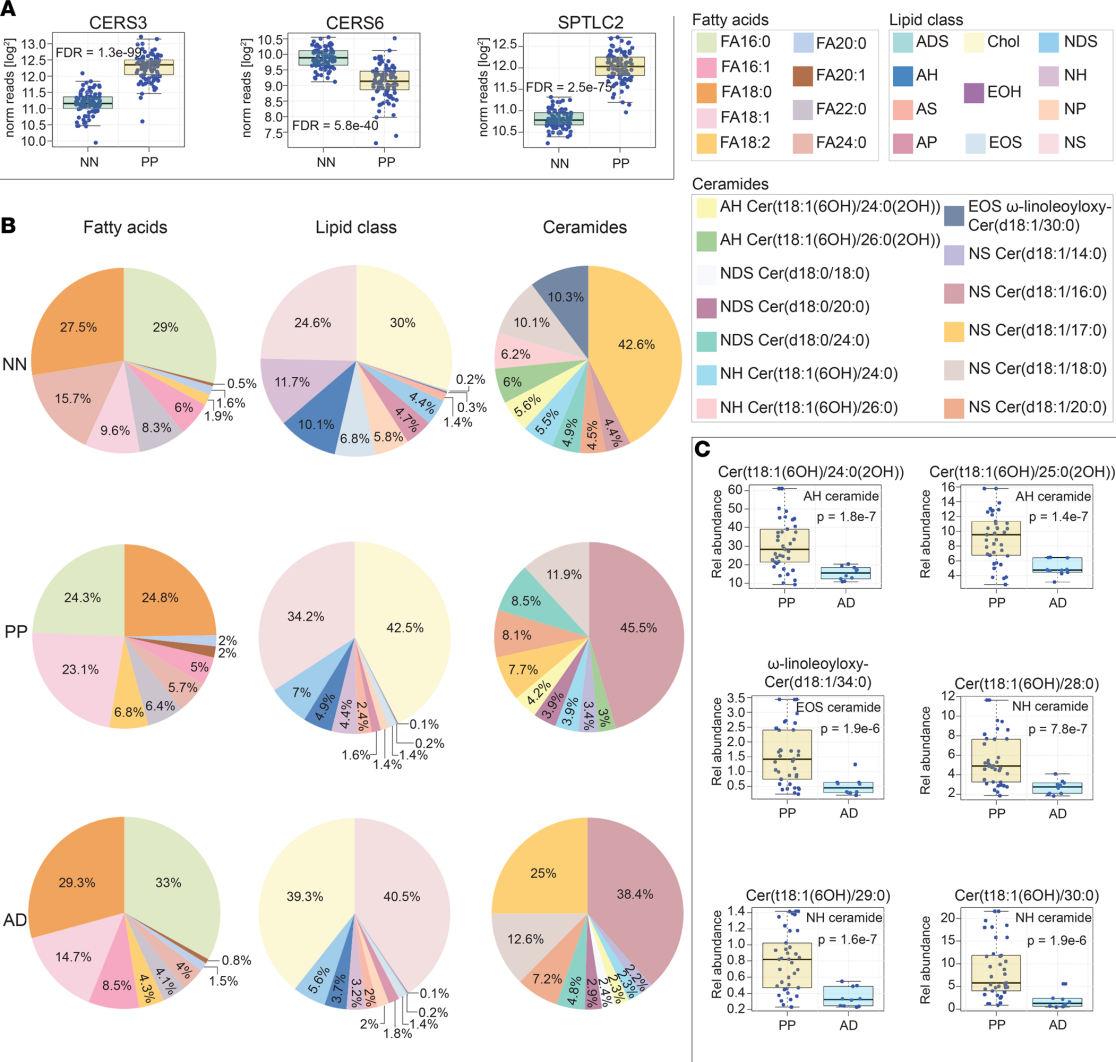
Similarities and differences between the lipid alterations seen in atopic dermatitis and psoriasis. (**A**) Whole-tissue RNA-Seq of psoriasis lesional skin identifies differentially expressed lipid-associated metabolic genes. Results are presented as box-and-whisker plots. (Additional gene expression data is presented in Supplemental Figure 8.) (**B**) Pie charts of epidermal lipid expression in control healthy skin, lesional atopic dermatitis skin, and lesional psoriasis skin. Note that 100% equals the combined abundance for listed lipids, not all epidermal lipids. The pie charts on the left are of representative fatty acids. The relative abundances of cholesterol and ceramides subclasses are depicted in the pie charts in the center. Pie charts on the right are of representative ceramides. Note that the strong upregulation of NS ceramide Cer(d18:1/16:0) in psoriasis makes it difficult to appreciate changes in other ceramide structures. (**C**) Examples of differentially expressed epidermal ceramides in psoriasis versus atopic dermatitis.

Similarly, characterization of the epidermal lipids in the setting of AD identified 115 lipids that were altered in lesional skin versus paired nonlesional skin from the same patients (FDR < 0.05, Supplemental Table 7). AD lesional skin was found to have a strong upregulation of the NS ceramide Cer(d18:1/16:0) (Figure 8A and Figure 9B). When AD was compared directly to psoriasis (AD versus PP), 82 differentially expressed lipids were identified (Supplemental Table 8), with the AH ceramide Cer(t18:1[6OH]/25:0[2OH]) being the most significant (*P =* 1.4 × 10^–7^) (Figure 9C).

To visualize the major lipid compositional differences between psoriasis, AD, and healthy controls, we constructed 3 different types of pie charts that displayed (a) the relative abundance of the different lipid classes, (b) the relative abundance of select FAs and Chol-SO4, and (c) the relative abundance of select ceramides. In all the above pie charts, 100% equals the combined total of only the listed lipids — not every epidermal lipid (Figure 9B). Clearly highlighted in these diagrams is the increased relative expression of Chol-SO4 in lesional psoriasis and AD skin. Psoriasis skin was also low in the saturated FA 24:0. In contrast, unsaturated FAs 18:1 and 18:2 were upregulated in psoriasis skin (Figure 9B). In terms of ceramides, the NS ceramide Cer(d18:1/16:0) was again found to be increased both in psoriasis and AD skin (Figure 9B). Although additional differences were present, the strong expansion of NS ceramide Cer(d18:1/16:0) and Chol-SO4 in psoriasis and AD decreased the percentiles of the less-abundant pie-chart–displayed lipids, making them difficult to see.

**Figure 10:**
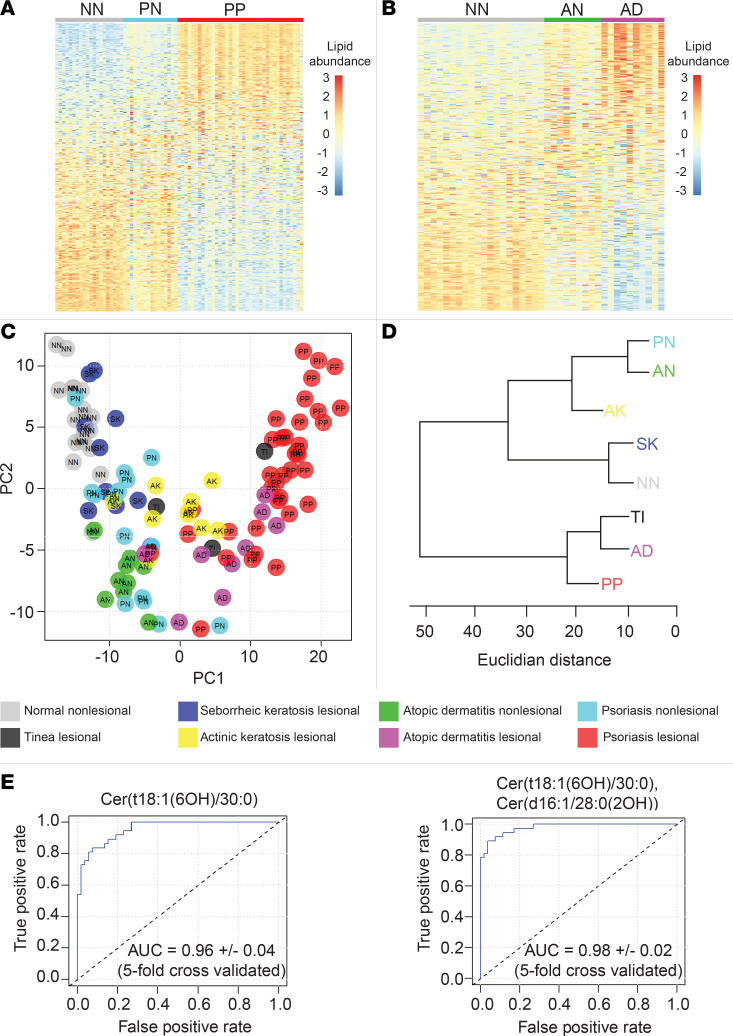
Epidermal lipid expression can diagnose skin diseases. Tape strippings were performed on lesional skin and control healthy skin. Each column represents a stratum corneum tape-stripping sample (grouped by diagnostic category). (**A**) Heatmap of lipid abundance in psoriasis (NN *=* normal healthy skin [gray, *n =* 20], PN = nonlesional psoriasis skin [blue, *n =* 16], and *P =* lesional psoriasis skin [red, *n =* 37]). Each row represents a monitored lipid, with red representing increased expression and blue representing decreased expression. Rows were sorted by standard mean difference (SMD). To construct heat maps to compare lipid abundances, lipid peak intensity values were preprocessed using the “scale and center” function in R, which subtracts the mean value of the analyte and divides the result by the standard deviation for that analyte. (**B**) Heatmap of lipid abundance in atopic dermatitis (AN *=* nonlesional atopic dermatitis skin [green, *n =* 10] and AD = lesional atopic dermatitis skin [purple, *n =* 9]). (**C**) Principal component analysis of relative lipid abundance data. Each dot represents 1 epidermal sample. Each color represents a diagnostic group (normal healthy skin [light gray], psoriasis lesional [red], psoriasis nonlesional [light blue], atopic dermatitis lesional [purple], atopic dermatitis nonlesional [green], actinic keratosis lesional [yellow], seborrheic keratosis lesional [dark blue], and tinea lesional [dark gray]). (**D**) Cluster dendrogram with Euclidian distance represented on the horizontal axis (AD, atopic dermatitis lesional; AK, actinic keratosis lesional; AN*,* atopic dermatitis nonlesional; NN*,* normal healthy skin; PN*,* psoriasis nonlesional; PP*,* psoriasis lesional; TI, tinea lesional). (**E**) Left: Receiver operating characteristic curve (ROC) for the single analyte classifier, NH ceramide Cer(t18:1[6OH]/30:0), demonstrates its ability to distinguish psoriatic lesional skin (PP) from all other diagnostic groups (NN, PN, PP, AK, SK, TI, and AD) combined (AUC, 0.96). Right: ROC for the 2-analyte classifier, NH ceramide Cer(t18:1[6OH]/30:0) + AS ceramide Cer(d16:1/28:0[2OH]), capable of distinguishing psoriatic lesional skin from all other diagnostic groups combined. The AUC for the 2-analyte classifier was 0.98 ± 0.02 (5-fold cross-validated).

To better appreciate alterations in lower abundance lipids, we next constructed heatmaps displaying the relative expression of all 351 monitored lipids in AD and psoriasis compared with healthy controls (Figure 10, A and B, respectively). Although skin affected by psoriasis, AD, AK, and TI all differed dramatically from healthy control skin, there was considerable overlap among these diagnostic groups when their lipid compositions were represented as a 2-dimensional PCA biplot (Figure 10C). SK and normal healthy skin (NN) were also highly overlapping in the PCA plot (Figure 10C).

To further visualize the lipid similarities between diagnostic groups, a cluster dendrogram was constructed (Figure 10D). The resulting diagram clearly demonstrates that some diagnostic groups clustered together and, thus, have similar epidermal lipid expression profiles. For example, normal skin (NN) clustered with SK skin, while inflamed skin (e.g., PP, AD, and TI) formed a separate cluster (Figure 10D).

### Disease-associated alterations in epidermal lipid genes.

To investigate why the expression profile of epidermal lipids in lesional psoriasis and AD skin differed from that of healthy controls, we next analyzed AD and psoriasis RNA-Seq data sets (NCBI GEO accession no. GSE121212, AD [*n =* 27], PSO [*n =* 28], control [*n =* 38]) to search for transcriptome alterations that could account for these changes (Figure 9A and Supplemental Figure 8). Predictions as to how the psoriasis-associated alterations in *CERS* gene expression affects ceramide synthesis are shown in Figure 8D. As expected, there was a clear link between psoriasis-associated lipid gene expression and psoriasis-associated alterations in lipid expression. For example, the ceramide abundances in psoriasis lesional skin matched the increased expression of *CERS3* and *SPTLC2* and the decreased expression *CERS6* (Figures 8D and 9A). A similar analysis linking AD lipid gene expression to the AD epidermal lipid expression is shown in Supplemental Figure 10.

Finally, to determine how the inflammatory milieu in psoriasis might contribute to the observed changes in epidermal lipid expression, we cultured 50 primary keratinocyte cell lines in parallel with the psoriasis-associated cytokines IFN-γ, IL-17A, and TNF and then characterized the expression of their lipid-associated metabolic genes by RNA-Seq. Results demonstrate that psoriasis-associated cytokines can alter the expression of lipid-associated genes, mimicking their expression pattern in psoriasis lesional skin. For example, TNF and IL-17A increased the expression of *SPTLC2*. Also, TNF in combination with IL-17A or IFN-γ alone decreased the expression of *CERS6* (Supplemental Figure 9B). These patterns match the expression of these lipid genes seen in psoriatic skin (Figure 9A).

### Lipid diagnostic classifiers.

For the primary care practitioner, it can be difficult to distinguish psoriasis from other papulosquamous skin disorders. Thus, having identified hundreds of disease-associated epidermal lipids, we next sought to characterize their performance as single analyte diagnostic classifiers by constructing receiver operating characteristic (ROC) curves for each of them. This analysis revealed that the NH ceramide Cer(t18:1[6OH]/30:0) was an accurate single analyte classifier of psoriasis (AUC = 0.96 ± 0.04, 5-fold cross-validation) (Figure 10E). Specifically, it could accurately distinguish psoriasis tape strippings from those taken from normal, AD, AK, SK, TI, and nonlesional psoriasis skin (Supplemental Figure 11A). Cer(t18:1[6OH]/30:0) was also differentially expressed when psoriasis lesional skin was compared with anatomically paired nonlesional skin from the same patients (Supplemental Figure 7A), and its abundance did not significantly vary across different body sites (Supplemental Table 2). The NH ceramide Cer(t16:1[6OH]/26:0) demonstrated similar utility as AD-specific diagnostic classifiers (AUC = 0.90 ± 0.09) (Supplemental Figure 11B). Other single analyte ceramides with diagnostic utility included the NDS ceramide Cer(d17:0/26:0), the AH ceramide Cer(t17:1[6OH]/26:0[2OH]), and the NH ceramide Cer(t18:1[6OH]/25:0), which could identify skin tape strippings from AK, SK, and TI, respectively (AUC = 0.86 ± 0.04, 0.92 ± 0.08, and 0.93 ± 0.12, respectively) (Supplemental Figure 11B).

We next used a step-forward algorithm to construct a multianalyte classifier specific for lesional psoriasis (*n =* 37, PP) against all other diagnostic groups (*n =* 77, total for AD, AK, AN, NN, PN, SK, TI). This model was composed of 2 lipid analytes, NH ceramide Cer(t18:1[6OH]/30:0) and AS ceramide Cer(d16:1/28:0[2OH]),(5.45*[NH ceramide Cer(t18:1(6OH)/30:0)]-1.74*[AS ceramide Cer(d16:1/28:0[2OH])]-12.16) (Figure 10E). Interrogation of the 2-analyte psoriasis prediction model revealed low collinearity among its analytes (variance inflation factor [VIF] = 1.49). The model was then validated using the K-fold cross-validation method (AUC = 0.98 ± 0.02, 5-fold cross-validated; Figure 10E).

### Interperson differences in epidermal lipid expression.

Environmental factors and genetics undoubtedly affect lipid composition, contributing to interperson variations in epidermal lipid expression. To characterize interperson differences in epidermal lipid expression, we used the body site epidermal lipid data set to construct a linear mixed-effects model, with body site and sex set as fixed effects, and person as a random effect. This model identified 97 lipids whose expression was interperson dependent. The lipids most associated with interperson variation were the structurally related AH ceramides Cer(t22:1[6OH]/26:0[2OH]), Cer(t20:1[6OH]/24:0[2OH]), Cer(t19:1[6OH]/24:0[2OH]) (FDR = 9.29 × 10^–7^, 1.3 × 10^–5^, 1.3 × 10^–5^, respectively) (Supplemental Table 2).

### Highly characteristic patterns of epidermal lipid expression.

**Figure 11:**
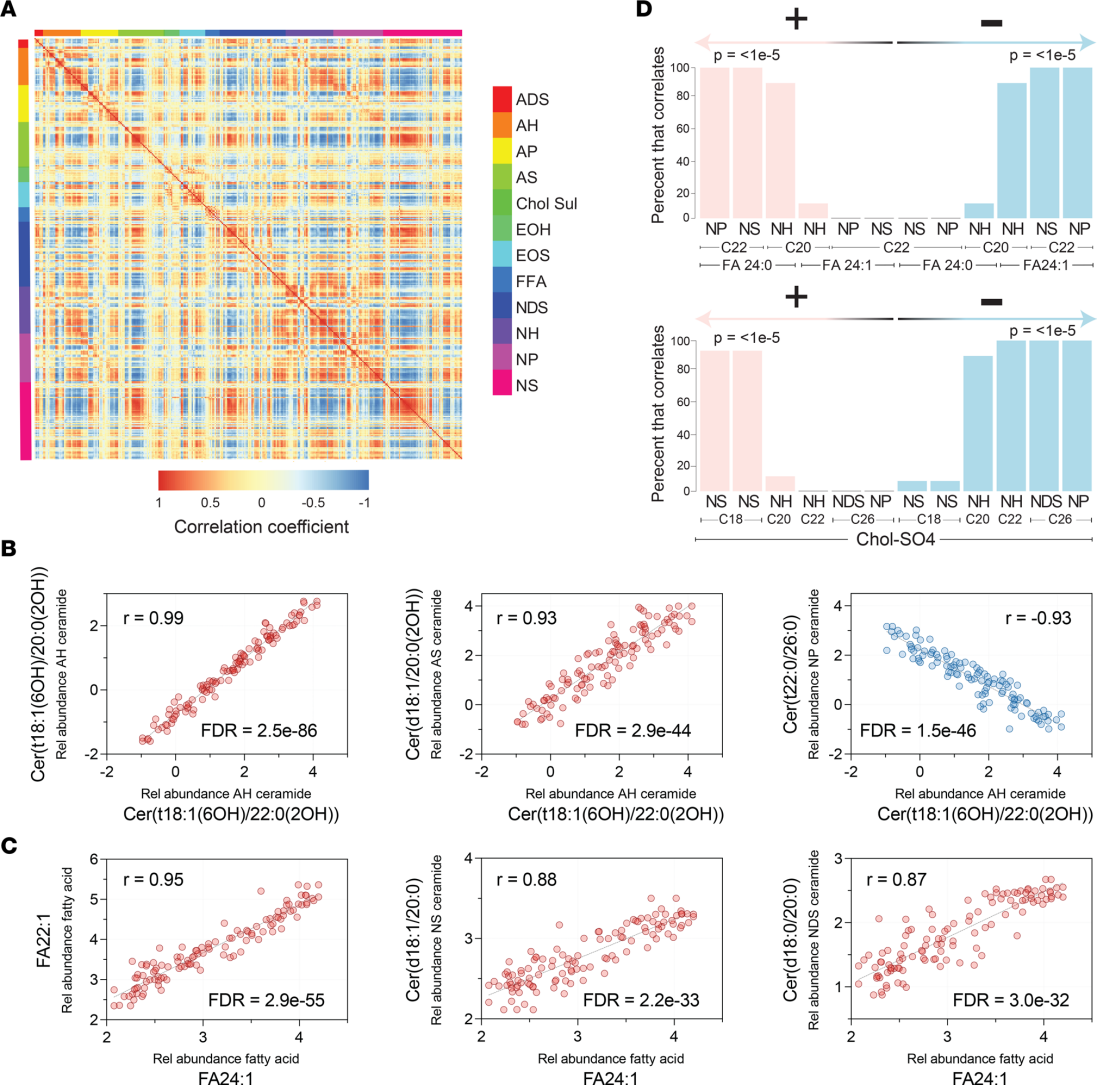
Analysis of epidermal lipid expression uncovers highly significant lipid-lipid expression patterns. (**A**) Correlation matrix depicting every possible pairwise lipid-lipid correlation among the 351 monitored lipids, with 123,201 combinations in total. The 351 columns and rows represent the monitored lipid analytes. The intensity of the color at the intersect between a column and row represents the strength of the correlation for that particular lipid-lipid combination (positive correlation*,* red; negative correlation*,* blue; no correlation, yellow). The checkerboard pattern indicates a consistent pattern of intraclass and interclass lipid correlations. (**B**) Scatter plots of representative lipid-lipid correlations. Intrasubclass ceramides with the same sphingoid base and similar fatty acid moieties positively correlated with one another. For example, the AH ceramide Cer(t18:1[6OH]/20:0[2OH]) positive correlated with AH ceramide Cer(t18:1[6OH]/22:0[2OH]) (*r* = 0.99, FDR = 2.5 × 10^–86^). Likewise, interclass ceramides with the same length sphingoid base and the same or similar fatty acid moieties positively correlated with one another. Shown here, the AS ceramide Cer(d18:1/20:0[2OH]) positively correlated with AH ceramide Cer(t18:1[6OH]/22:0[2OH]) (*r* = 0.93, FDR = 2.9 × 10^–44^). Also, 18-carbon sphingoid base ceramides negatively correlated with 20- and 22-carbon sphingoid base ceramides, usually with dissimilar length fatty acids. Also shown, the NP ceramide Cer(t22:0/26:0) negatively correlated with the AH ceramide Cer(t18:1[6OH]/22:0[2OH]) (*r* = –0.93, FDR = 1.5 × 10^–46^). (**C**) Unsaturated fatty acids of similar length tended to positively correlate with one another. Shown here, FA 22:1 positively correlated with FA 24:1 (*r* = 0.95, FDR = 2.9 × 10^–55^). FA 24:1 (and to a lesser extent FA 22:1 and sometimes FA 20:1) positively correlated with NS(C18) and NDS(C18) ceramides. Also shown, FA 24:1 positively correlated with the NS ceramide Cer(d18:1/20:0) (*r* = 0.88, FDR = 2.2 × 10^–33^) and the NDS ceramide Cer(d18:0/20:0) (*r* = 0.87, FDR = 3.0 × 10^–32^). (**D**) Bar graphs illustrate the percent of ceramides that follow the patterns described in **B**.

**Figure 12:**
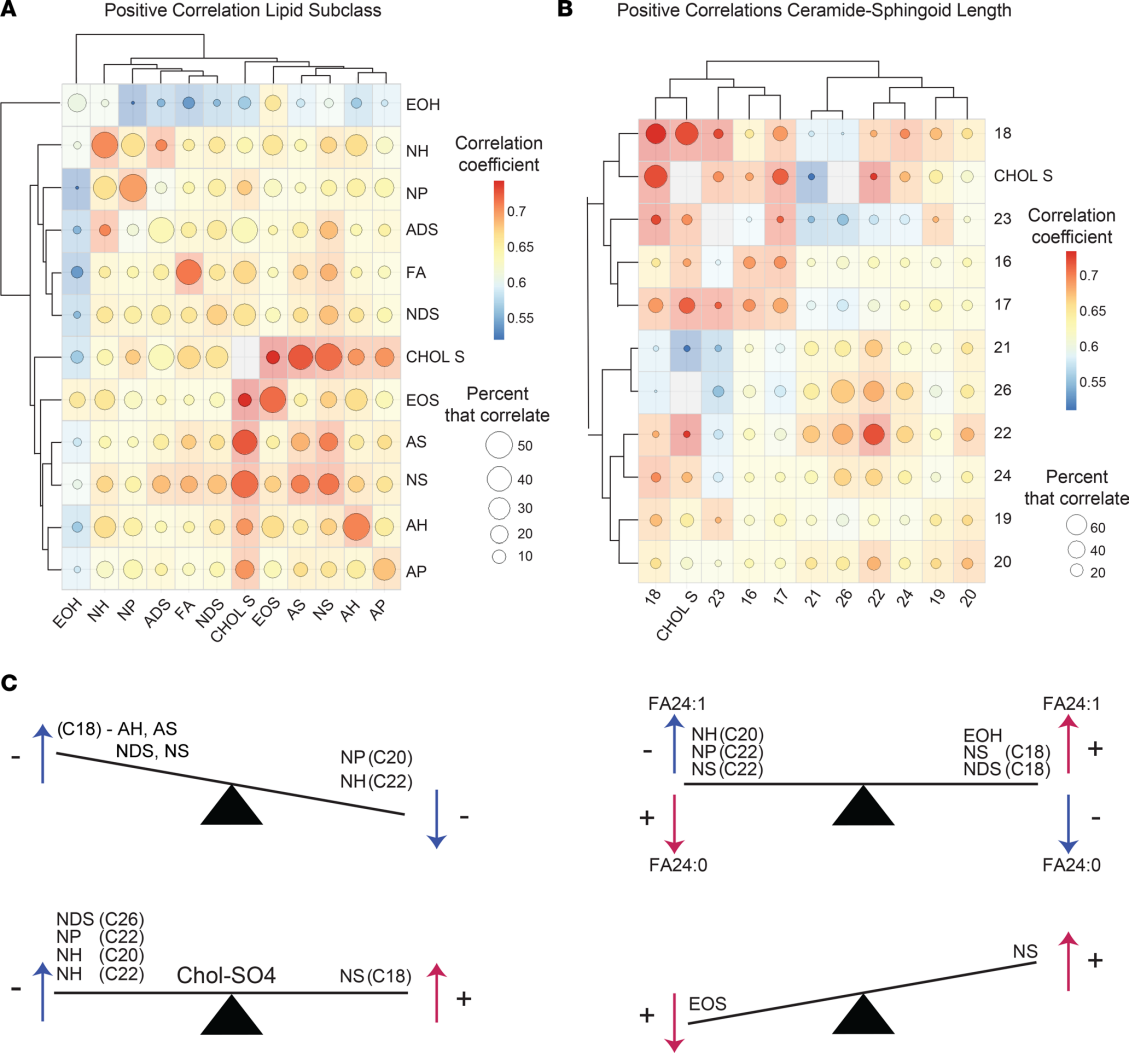
Lipid expression patterns. (**A**) Correlation matrix representing the patterns of positive correlations among different lipid subclasses. The intensity of the color at the intersect between a column and row represents the average correlation coefficient for that particular lipid subclass combination. The size of the circle within each colored box represents the percent of lipids that positively correlated. Hierarchical clustering was used to order the lipid subclasses based on the similarities between their patterns of correlation. (**B**) Ceramides were grouped by the length of their sphingoid bases. Lipid expression across different groups was then assessed, and a correlation matrix was constructed. The intensity of color at the intersect between a column and row represents the average positive correlation coefficient for that particular group comparison. The size of the circle within each colored box represents the percent of lipids that correlated. (**C**) Schematic seesaw diagrams depicting common patterns of lipid-lipid correlations.

After characterizing the biogeography of epidermal lipid expression and its disease-associated alterations, we next sought to determine how the expression of individual lipids related to one another. For this, we first pooled data from each diagnostic group and then calculated lipid correlation coefficients for every possible lipid-lipid comparison, 123,201 in total (Supplemental Table 9). This identified over 22,663 significant (FDR < 0.05) lipid-lipid correlations (Supplemental Table 9). To visualize the resulting 123,201 correlations, we constructed a correlation matrix (Figure 11A), which utilized a color scale to visualize the strength of each lipid-lipid correlation. A repeating quadrangle color pattern is seen upon visual inspection of the matrix, indicating that structurally related lipids tended to correlate with one another. This was also evident when correlation matrices were constructed to compare the different lipid classes and subclasses (Figure 12A and Supplemental Figure 12C, E, and F). From these diagrams, the following generalizations could be made: (a) the strongest positive correlations were usually between lipids of the same subclass; (b) Chol-SO4 tended to positively correlate with ADS, NS, and AS ceramides and to negatively correlate with AH, NH, and NP ceramides; (c) EOH ceramides tended to correlate with EOS ceramides; (d) NH ceramides tended to positively correlate with NP ceramides; and (e) NS ceramides tended to positively correlate with AS ceramides. Ceramide sphingoid base carbon length matrices (Figure 12B and Supplemental Figure 12 B and D) revealed that (a) ceramides with similar length sphingoid bases tended to correlate well with one another, (b) Chol-SO4 correlated positively with 17- or 18-carbon sphingoid base ceramides and negatively with 26-carbon sphingoid base ceramides, and (c) 22-carbon sphingoid base ceramides positively correlated with 26-carbon sphingoid base ceramides, which both negatively correlated with 28-carbon sphingoid base ceramides (Figure 12B and Supplemental Figure 12D).

As another means to visualize all lipid-lipid correlations, we next applied the t-distributed stochastic neighbor embedding (t-SNE) dimensionality reduction strategy, using the pairwise distance formula, 1 – *r*^2^, where *r* represents the Pearson’s correlation coefficient of each lipid-lipid comparison. From the resulting image, a variety of strong intra- and intersubclass lipid-lipid correlations were apparent. The t-SNE plot also clearly demonstrated that there are groups of lipids from within each subclass that have similar correlation patterns, but not all lipids from within the same subclass behave similarly (Supplemental Figure 12A).

Following these visualization strategies, scatter plots for every lipid-lipid comparison were constructed to allow for a more in-depth analysis of the lipid-lipid coexpression profiles (Figure 11, B and C, and Supplemental Figure 13). For example, intrasubclass ceramides with similar length sphingoid bases and the same or similar length FA moieties positively correlated with one another — e.g., AH ceramide Cer(t18:1[6OH]/20:0[2OH]) positively correlated with AH ceramide Cer(t18:1[6OH]/22:0[2OH]) (*r* = 0.99, FDR = 2.5 × 10^–86^) (Figure 11B). Likewise, interclass ceramides with similar length sphingoid bases that share a common FA moiety tended to correlate with one another — e.g., NH ceramide Cer(t18:1[6OH]/30:0) positively correlated with NS ceramide Cer(d18:1/30:0) (*r* = 0.94, FDR = 3.0 × 10^–49^) (Supplemental Figure 13A) and AH ceramide Cer(t18:1[6OH]/22:0[2OH]) positively correlated with AS ceramide Cer(d18:1/22:0[2OH]) (*r* = 0.93, FDR = 2.9 × 10^–44^) (Figure 11B). Negative lipid-lipid correlations also followed distinct patterns. For example, ceramides with 18-carbon sphingoid bases tended to negatively correlate with ceramides containing 20 or 22 carbon sphingoid bases, usually with dissimilar length FAs. The strongest negative correlations were between the AH(C18), AS(C18), and NDS(C18) ceramides and the NP(C20 or C22) and NH(C20 or C22) ceramides. For example, AH ceramide Cer(t18:1[6OH]/22:0[2OH]) negatively correlated with NP ceramide Cer(t22:0/26:0) (*r* = –0.93, FDR = 1.5 × 10^–46^) (Figure 11B), and AH ceramide Cer(t18:1[6OH]/20:0[2OH]) negatively correlated with NP ceramide Cer(t22:0/26:0) (*r* = –0.93, FDR = 9.2 × 10^–45^) (Supplemental Figure 13B). NS(C18) ceramides also negatively correlated with NP(C22) ceramides (e.g., NS ceramide Cer[d18:1/23:0] negatively correlated with NP ceramide Cer[t22:0/25:0]; *r* = –0.88, FDR = 6.7 × 10^–34^) (Supplemental Figure 13B).

In addition to the aforementioned ceramide-ceramide correlative patterns, lipid correlations involving FAs also followed specific rules. The strongest positive correlations were seen among unsaturated FAs of similar length — e.g., FA 22:1 positively correlated with FA 24:1 (*r* = 0.95, FDR = 2.9 × 10^–55^) (Figure 11C). Likewise, saturated FAs of similar length also tended to positively correlate with one another — e.g., FA 16:0 positively correlated with FA 18:0; *r* = 0.94, FDR = 4.9 × 10^–49^) (Supplemental Figure 13C). FA 24:1 tended to have strong negative correlations with NH(C20), NP(C22), and NS(C22) ceramides (Figure 11D)— e.g., FA 24:1 negatively correlated with NH ceramide Cer(t20:1[6OH]/27:0) (*r* = –0.81, FDR = 2.97 × 10^–23^) and NP ceramide Cer(t22:0/26:0) (*r* = –0.80, FDR = 3.96 × 10^–22^) (Supplemental Figure 13D). In contrast, FA 24:1 (and, to a lesser extent, FA 22:1 and FA 20:1) tended to positively correlate with NS(C18) and NDS(C18) ceramides — e.g., FA 24:1 positively correlated with NS ceramide Cer(d18:1/20:0) (*r* = 0.88, FDR = 2.2 × 10^–33^) and NDS ceramide Cer(d18:0/20:0) (*r* = 0.87, FDR = 3.0 × 10^–32^) (Figure 11C). In contrast, saturated FA 24:0 tended to have strong positive correlations with NDS(C24) and NDS(C26) ceramides, as well as NP(C22) and NS(C22) ceramides (Figure 11D), and it tended to have negative correlations with NS(C18) ceramides (Supplemental Table 9). Finally, there was a strong inverse correlative relationship between FA 24:1 and FA 24:0, presented graphically as a seesaw diagram (Figure 12C).

Correlative patterns were also noted for Chol-SO4. Specifically, Chol-SO4 tended to positively correlate with 18-carbon sphingoid base ceramides, especially NS(C18) ceramides — e.g., Chol-SO4 positively correlates with NS ceramide Cer(d18:1/23:0) (*r* = 0.80, FDR = 3.7 × 10^–22^) (Figure 11D and Supplemental Figure 13E). In contrast, Chol-SO4 tended to negatively correlate with 20, 22, and 26 carbon sphingoid base ceramides, especially NH(C20 and C22) ceramides, NDS(C26) ceramides, and NP(C26) ceramides (Figure 11D) — e.g., Chol-SO4 negatively correlated with NH ceramide Cer(t20:1[6OH]/27:0) (*r* = –0.78, FDR = 1.4 × 10^–19^) (Supplemental Figure 13E and Supplemental Table 9). Finally, EOH ceramides positively correlated with their counterpart EOS ceramides. For example, EOH ceramide ω-linoleoyloxy-Cer(t20:1[6OH]/32:0) strongly correlated with EOS ceramide ω-linoleoyloxy-Cer(d20:1/32:0) (*r* = 0.83, FDR = 7.5 × 10^–26^) (Supplemental Figure 13F). They also correlated with ceramides of similar structure within their own subclass — e.g., EOS ceramide ω-linoleoyloxy-Cer(d20:1/29:0) positively correlated with EOS ceramide ω-linoleoyloxy-Cer(d20:1/31:0) (*r* = 0.93, FDR = 2.1 × 10^–45^) (Supplemental Figure 13F). However, in contrast to EOH ceramides, EOS ceramides more strongly correlated with NS ceramides of similar structure — e.g., EOS ceramide ω-linoleoyloxy-Cer(d22:1/31:0) positively correlated with NS ceramide Cer(d22:1/27:0) (*r* = 0.88, FDR = 2.8 × 10^–33^) (Supplemental Figure 13F). As a general rule, correlations between EOS and NS ceramides were stronger than corresponding EOH and NS correlations (Supplemental Table 9).

### Coexpression of epidermal lipid gene with skin barrier and immune genes.

**Figure 13:**
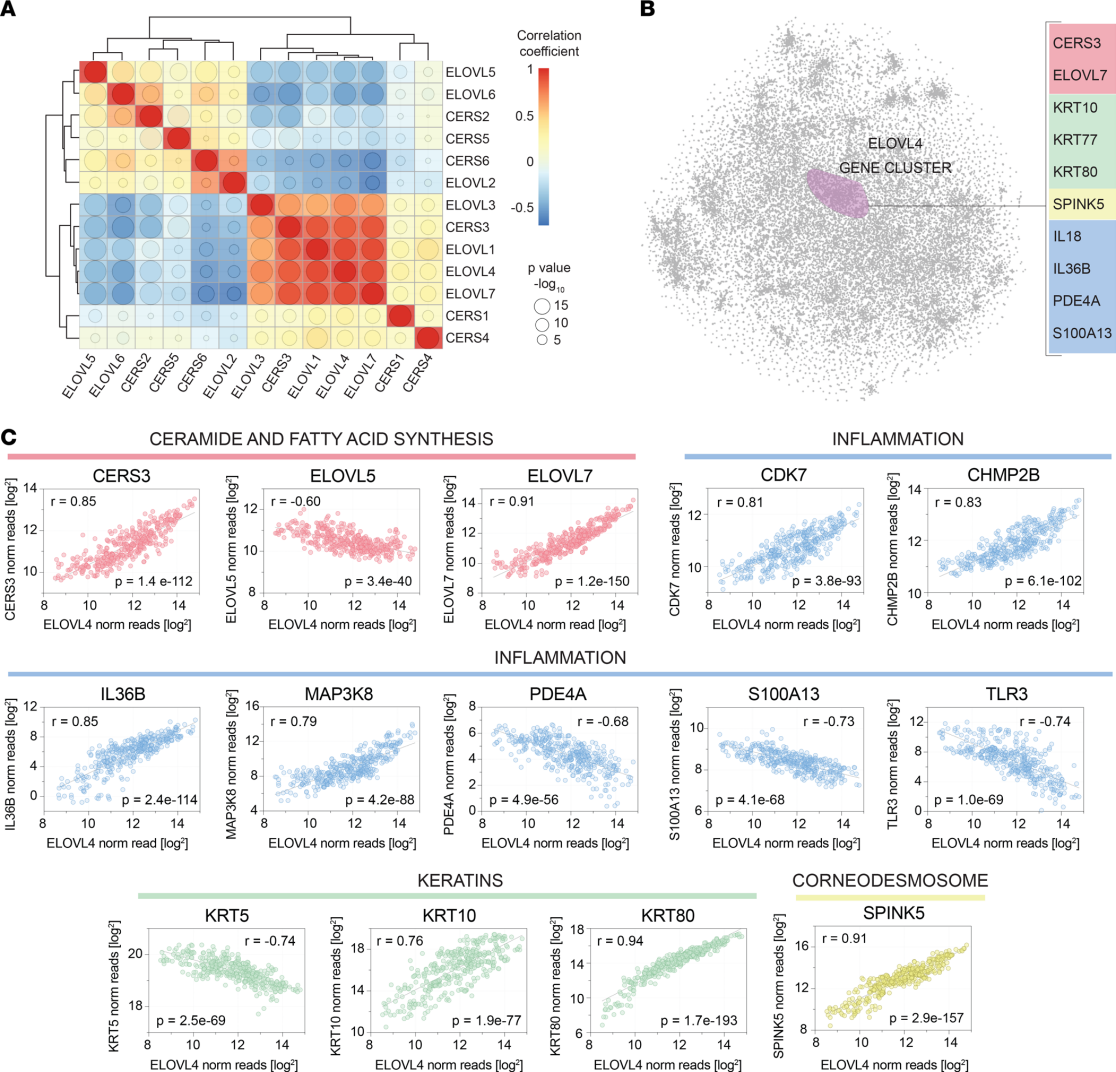
The *ELOVL4* expression correlates with immune and skin barrier genes. (**A**) Hierarchical clustering of lipid genes by their expression yields 2 clear clusters, one centered on *ELOVL4* and the other on *ELOVL6*. The lipid genes coexpressed with *ELOVL4* include *ELOVL1*, *ELOVL3*, *ELOVL7*, and *CERS3*. These genes negatively correlated with the lipid-genes within the *ELOVL6* cluster (*ELOVL5, ELOVL6,* *CERS2*, *CERS5, CERS6,* and *ELOVL2)*. The size of the circle within each box is proportional to the significance of the intersecting lipid-lipid correlation, while the color represents the correlation coefficient of the comparison. (**B**) The t-SNE nonlinear dimensionality reduction method was used to create a 2-dimensional plot of the keratinocyte transcriptome from RNA-Seq data obtained from 50 primary human keratinocytes cell lines. Within this plot, each point represents a keratinocyte-expressed gene, and the distance between the points is inversely related to how strongly the genes correlated with one another. Representative genes that cocluster with *ELOVL4* are listed on the right, and they include various inflammatory mediators (e.g., *IL36B*, in blue). (**C**) Individual gene expression scatter plots reveal strong correlations between the expression of *ELOVL4* (*x* axis) and representative coclustering genes (*y* axis). In these plots, each dot represents a unique in vitro cultured primary human keratinocyte cell line and culture condition (50 unique primary human keratinocyte cell lines were each cultured under 8 different conditions; see Supplemental Methods). *ELOVL4* strongly correlated with lipid genes involved in ceramide and fatty acid synthesis (pink), as well as select keratin (green) and corneodesmosone-related (yellow) genes. Note the strong correlation between *ELOVL4* and various immune-related genes (blue).

**Figure 14:**
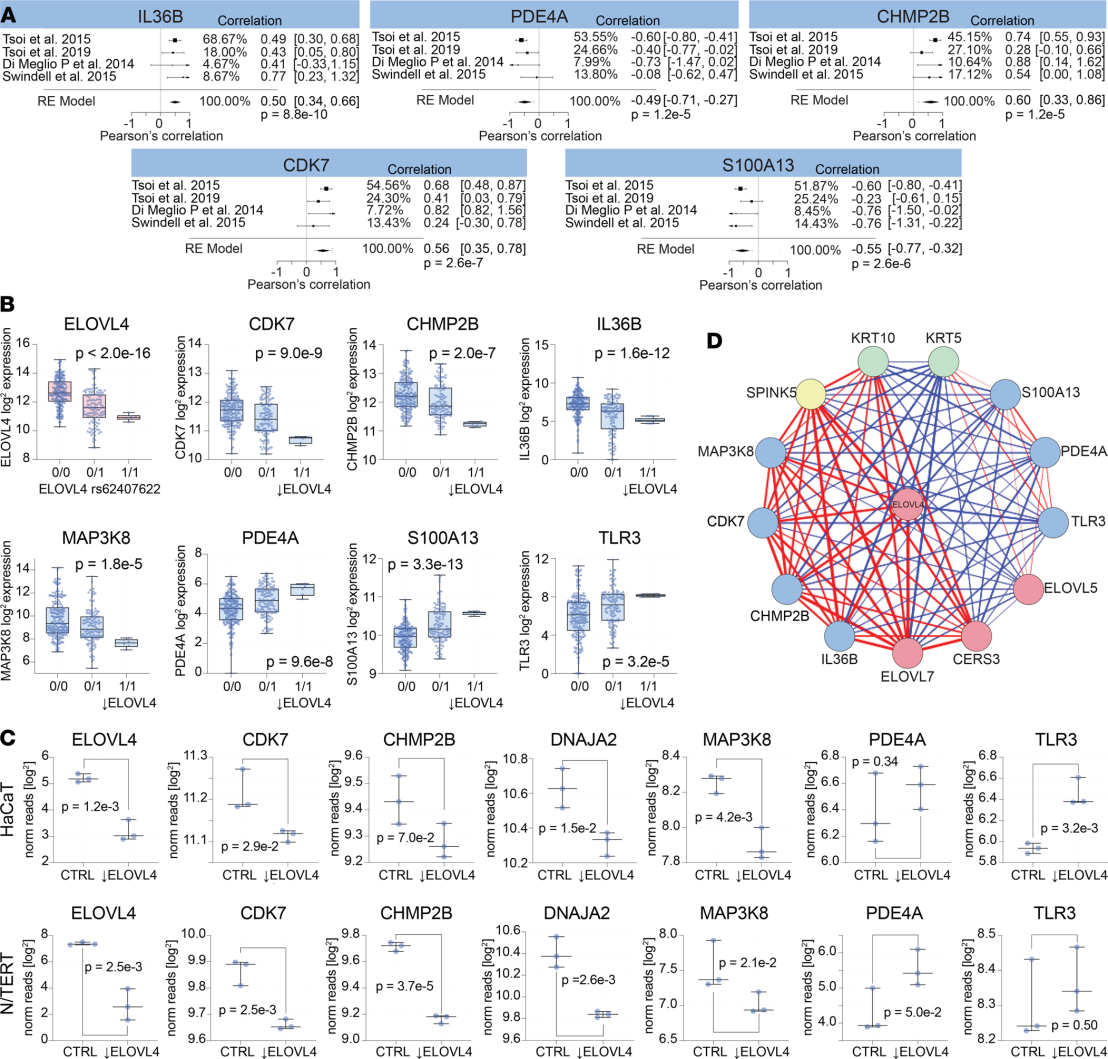
Altered *ELOVL4* expression impacts expression of immune genes in keratinocytes. (**A**) RNA-Seq data sets of psoriasis lesional skin were mined for gene expression of *ELOVL4*, *IL36B*, *PDE4A*, *CHMP2B*, *CDK7*, and *S100A13*. These genes were selected because they were highly coexpressed with *ELOVL4* in primary human keratinocytes and are known inflammatory mediators that are differentially expressed in psoriasis lesional skin. A metaanalysis was performed to combine the *ELOVL4* coexpression results across 4 independently acquired RNA-Seq data sets. Forest plots are presented, and *P* values for the final model are shown. (**B**) Keratinocyte RNA-Seq data sets were parsed into 3 groups based on the *ELOVL4* allele they expressed (0/0 representing the *ELOVL4* reference allele, and 0/1 and 1/1 representing heterozygosity and homozygosity for the *ELOVL4* variant, rs62407622). Box plots of ELOVL4 expression revealed that keratinocytes homozygous for rs62407622 expressed significantly reduced levels of *ELOVL4*. Keratinocytes homozygous for the ELOVL4^lo^ variant also expressed significantly lower levels of *CDK7*, *CHMP2B*, *IL36B*, and *MAP3K8* and significantly increased levels of *PDE4A*, *S100A13*, and *TLR3*. (**C**) HaCat and N/TERT immortalized keratinocyte cell lines were treated with *ELOVL4* siRNA or control scrambled RNA. *ELOVL4* siRNA knockdown significantly downregulated expression of *CDK7*, *CHMP2B*, *DNAJA2*, and *MAP3K8*. It also increased expression of PDE4A in N/TERT cells and TLR3 in HaCaT cells. (**D**) *ELOVL4* coexpression network. Line thickness is directly proportional the correlation coefficient for the coexpression of the connecting genes. Red lines indicate a positive correlation, and blue lines indicate a negative correlation.

Since various highly significant lipid-lipid correlations were identified, we next sought to determine if the expression of respective lipid genes were also highly correlative. First, we assessed the expression of the *ELOVL* and *CERS* lipid-gene family members in 50 primary keratinocyte cell lines. This analysis revealed that the ELOVL and CERS genes formed 2 clusters based on their strong correlative relationships, the *ELOVL4* cluster (*ELOVL1*, *ELOVL4*, *ELOVL7*, and *CERS3*) and the *ELOVL6* cluster (*ELOVL2*, *ELOVL5*, *ELOVL6*, *CERS2*, and *CERS5*) (Figure 13A). A 2D plot of the keratinocyte transcriptome demonstrated that *ELOVL4* also clustered with various genes involved in autoinflammatory immune responses — e.g., *IL36B* and *IL18* (Figure 13B). A scatter plot of *ELOVL4* versus *IL36B* expression in cultured keratinocytes supported the relationship between these 2 genes (Figure 13C). Several other genes were also highly correlative with *ELOVL4* (Figure 13C), including a few keratin genes (*KRT5*, *KRT10*, and *KRT80*) and various genes known to be involved in inflammation (*CDK7*, *CHMP2B*, *MAP3K8*, *TLR3*, and *S100A13*). The strong link between *ELOVL4* and *IL36B, CDK7, CHMP2B*, and *S100A13* was also evident in RNA-Seq data sets of psoriasis lesional skin (Figure 14A). To determine if these genes were simply coregulated with one another or if *ELOVL4* expression had a direct effect on the expression of the other genes, we searched for an *ELOVL4* variant associated with decreased *ELOVL4* expression. This identified a single nucleotide polymorphism (SNP), rs62407622, located within the ELOVL4 gene that was associated with a strong reduction in *ELOVL4*, both in primary keratinocytes (*P <* 2.0 × 10^–16^, Figure 14B) and lesional psoriasis skin (*P =* 2.1 × 10^–2^, Supplemental Figure 14). Keratinocytes were then parsed by their sequence at this locus (reference allele [0/0], heterozygous for rs62407622 [0/1], and homozygous for rs62407622 [1/1]). *CDK7*, *CHMP2B*, *PDE4A*, *IL18*, *IL36B*, *MAP3K8*, and various other lipid genes (*CERS3*, *SPTLC3*) and the barrier genes (*CSTA*, *KLK7*, *PDAI1*) were all decreased in the *ELOVL4* ^lo^ keratinocytes homozygous for rs62407622. In contrast, keratinocytes homozygous for rs62407622 had increased expression of *S100A13* and *TLR3* (Figure 14B). Similarly, when small interfering RNA (siRNA) was used to knock down the expression of *ELOVL4* in immortalized HaCaT and N/TERT keratinocyte cell lines, expression of *CDK7*, *CHMP2B*, and *MAP3K8* was decreased (Figure 14C), and in *ELOVL4* siRNA–knockdown HaCaT cells, *TLR3* expression was increased. A summary of the *ELOVL4* coexpression network, including its positive and negative associations, is shown in Figure 14D. This figure highlights the close relationship of *ELOVL4* with genes important for skin barrier, lipid synthesis, and inflammation.

## Discussion

In this study, we monitored 351 of the most abundant SC lipid structures using targeted mass spectrometry. The main advantage of using a targeted approach is the reproducibility and high-throughput nature of the technique (13), which makes it possible to accurately characterize biogeographic patterns of epidermal lipid expression and their disease-associated alterations. Our study builds on previous reports that have used shotgun mass spectrometry to characterize epidermal lipids (18) and those that have characterized the biogeographic distribution of sebum lipids (18).

Our results demonstrate that epidermal lipid composition is uniquely tuned to each body site. We hypothesize that these body site–specific differences optimize the skin for the biological insults encountered at each particular location. They help explain how the epidermis of the face is thin and flexible to meet the demands of facial expression and how the epidermis of the heel is thick and compact to resist trauma caused by punctures and friction. In total, we identified 272 lipid analytes that had differential expression across different body sites (FDR < 0.05, mixed-effects model) (Supplemental Table 2). These geographic patterns of lipid expression were more apparent for some ceramide classes than others. For example, ADS ceramides were more uniformly expressed across all anatomic locations. In contrast, NH, AH, and NP ceramides differed dramatically from one body site to the next (Figure 1). Thus, while epidermal lipid composition appears to be biogeographically tuned, certain lipids are more stably expressed across body sites than others. Likewise, some anatomic sites had more extreme lipid alterations than others, with the face (i.e., GB, AL, CK) and heel occupying opposite extremes of this natural variation.

Like ceramides, free FAs are also abundant in the epidermal lipidome, especially saturated FAs. Albeit less abundant, unsaturated FAs also make important contributions to the structure of the lipid matrix or “mortar.” This is of particular relevance because we found that unsaturated FAs had a high degree of biogeographic variation (Figure 2). For example, the unsaturated FAs 18:1 and 18:2 were strongly upregulated in PH skin (Figure 2). Likewise, the abundance of Chol-SO4 was also found to be highly upregulated in GB, AL, and CK skin (Figure 2).

Integrating site-specific lipid profiling with whole tissue and single-cell transcriptomics revealed a molecular basis for the observed biogeographic variation in epidermal lipid composition. For example, the differential expression of genes encoding the components of the serine palmitoyltranferase complex matched the biogeographic variation in ceramide sphingoid bases. Additionally, the differential expression of the ceramide synthases matched the biogeographic variation in ceramide FA moieties. Briefly, the serine palmitoyltranferase complex (either SPTLC1-SPTLC2-SPTSSA, SPTLC1-SPTLC3-SPTSSA, or SPTLC1-SPTLC3-SPTSSB) catalyzed the rate-limiting step in sphingolipid biosynthesis, with SPTLC2 favoring generation of 18-carbon sphingoid bases and SPTLC3 favoring generation of 16-carbon sphingoid bases; SPTLC3 also supported synthesis of longer carbon sphingolipids when present within SPTLC1-SPTLC3-SPTSSB. Single-cell RNA-Seq revealed that *SPTLC2* was upregulated and *SPTLC3* was downregulated in acral skin granular layer keratinocytes (Figure 5C). This expression pattern should favor synthesis of 18-carbon over 16-carbon sphingolipid base ceramides, which is what was observed. The specific 18-carbon sphingoid base ceramides that had the strongest upregulation in acral skin were synthesized with FA moieties 20–22 carbons in length (e.g., AH ceramide Cer[t18:1(6OH)/22:0(2OH)], Figure 4A). The preference for 18-carbon ceramides in acral skin to be synthesized with FAs 20–22 carbons in length is supported by the upregulation of *CERS4* in acral granular layer keratinocytes (Figure 5C). CERS4 synthesizes ceramides with medium length (C20–C22) FAs (Figure 5B). Also, the downregulation of *ABHD5* in acral skin (Supplemental Figure 4B) was consistent with the low expression of EOS ceramides in PH, especially the usually well-expressed EOS ceramides ω-linoleoyloxy-Cer(d18:1/30:0) and ω-linoleoyloxy-Cer(d18:1/32:0) (Figure 4B), as *ABHD5* has been shown to stimulate PNPLA1-mediated acyl-ceramide biosynthesis (19, 20). Furthermore, mice with epidermal deficiency in *UGCG* have reduced expression of EOS-GlcCers(d18:1) ceramides but increased expression of NS Cer(d18:1/18:0) (21). Therefore, the downregulation of *UGCG* in acral skin (Figure 5C) might be another reason for the relatively high expression of NS Cer(d18:1/18:0) in PH. These links between epidermal lipid-gene expression patterns and epidermal lipid composition would have been difficult to appreciate without single-cell sequencing. For example, *SPTLC2* was only significantly upregulated in granular layer acral keratinocytes. Finally, the low expression of cholesterol in PH matched the downregulation of cholersterol-associated genes, including the cholesterol-synthesizing enzyme DHCR24 and the cholesterol-binding protein apolipoprotein E (APOE) (Supplemental Figure 4A and Supplemental Figure 5A, respectively).

The biogeographic alterations in epidermal lipids just described will undoubtedly manifest as physical changes to the lipid “mortar” located between the corneocyte “bricks.” For example, the lipid envelope is a monolayer of FAs and acyl-ceramides esterified to the cornified envelope (22–24). It is the starting template for organizing the overlying lamellar sheets (24, 25), which are arranged vertically in either long periodicity phases (LPP) or short periodicity phases (SPP). In addition to being a major component of the lipid envelope, acyl-ceramides are also critical in the formation of LPPs. Epidermal lipids are also organized laterally in orthorhombic, hexagonal, or liquid arrangements. These lateral arrangements differ from one another in how densely packed their lipids are. The tendency for a particular lateral arrangement to be favored depends on (a) the abundance of acyl-ceramides (26), (b) the FA/ceramide/cholesterol ratio (27), (c) the chain length of the free FAs and ceramides (24, 28), and (d) the degree of FA saturation (24). We observed biogeographic alterations in each of these key parameters. Thus, epidermal lipids will be organized differently at each anatomic location. We speculate that the low abundance of EOS ceramides in PH skin, and the decrease in other long-chain ceramides, will favor shorter repeat distances and/or a reduced presence of LPP; this should make PH epidermis more compact and less susceptible to frictional forces, which would be ideal for this surface.

The end product of keratinocyte terminal differentiation is the corneocyte, which has a unique appearance at acral surfaces. The stratum lucidum, a hallmark of acral surfaces, is a translucent layer visible on microscopy located directly above the granular layer. The gene expression profiling presented here provides some insight into how acral corneocytes might form this structure. For one, we found that genes encoding filaggrin and keratin citrullination enzymes (*PADI1* and *PADI3*) were strongly downregulated in acral skin (Figure 6B). The differential expression of these filaggrin-altering enzymes will undoubtedly manifest as alterations to the intracellular processing of filaggrin and keratin. We speculate that the accentuation of the stratum lucidum at acral surfaces is a result of these alterations in filaggrin and keratin processing.

The ultra-thick SC is perhaps the most characteristic feature of acral skin. Acral skin, thus, requires a mechanism to delay corneocyte desquamation, thus allowing for additional layers of corneocytes to form. We used single-cell RNA-Seq to shed insight into this process. This analysis revealed that cornified envelope-associated genes (e.g., *SPRR1B* and *TGM5*) were upregulated in granular layer acral keratinocytes (Figure 6B and Supplemental Figure 5A). The cysteine protease inhibitors (e.g., *CSTA* and *CST3*; Figure 6B) were also strongly upregulated in granular layer acral keratinocytes. These changes are especially noteworthy because deficiencies of *TGM5* and *CSTA* have been associated with acral peeling skin syndrome. Thus, their upregulation in acral skin should help maintain acral corneocytes from desquamating. Genes encoding connexins, aquaporins, and intercellular disulfide-linked gap junction proteins and those encoding tight junction proteins were also upregulated in acral skin (Figure 6B). Increased expression of these proteins is another mechanism by which corneocytes are retained at acral surfaces. However, of all the corneocyte connections, the most important adhesive structures are the desmosome proteins, which ultimately give rise to corneodesmosomes. Single-cell RNA-Seq revealed that desmosome genes (e.g., *DSG1* and *DSC3*) are highly upregulated in granular layer acral keratinocytes (Figure 6A). Desquamation can only occur after these corneodesmosomes are broken down. It is, therefore, relevant that acral granular layer keratinocytes expressed low levels of *KLK1* and *KLK7*, which encode corneodesmosome-degrading kallikreins. Similarly, although *KLK5* was not found to be downregulated in acral keratinocytes (Figure 6A), the KLK5-specific inhibitor, *SPINK9,* was highly upregulated and its expression appeared to be specific for acral skin (Figure 6C). These alterations in gene expression suggest that acral skin corneocytes are held together by stronger or more numerous corneodesmosomes that are degraded by kallikreins at a slower rate.

Importantly, several of the genes reported here to be differentially expressed in spinous and granular layer acral keratinocytes have also been previously associated with epidermal skin diseases, especially the ichthyoses, a heterogeneous group of cornification disorders. For example, *KRT1* and *KRT10* have been associated with epidermolytic hyperkeratosis (EHK), an ichthyosis that is hallmarked by thick hyperkeratotic skin. Interestingly, *KRT1*-associated EHK has an accompanying palmoplantar keratoderma that is generally absent in *KRT10*-associated EHK. Palmoplantar keratoderma also happens to be the clinical manifestation of *KRT9* deficiency (16, 17). In our current report, we demonstrate that *KRT1* is strongly upregulated in acral keratinocytes. Furthermore, within the individual acral keratinocytes, this *KRT1* upregulation almost perfectly correlates with *KRT9* expression (Figure 7D), a keratin that is nearly exclusively expressed by acral suprabasal keratinocytes (Figure 7B). Thus, deficiencies in *KRT10* likely do not manifest as palmoplantar keratoderma because, at acral sites, *KRT9* is the main binding partner of *KRT1*. In acral keratinocytes, the expression of *KRT9* was also found to be 1.7-fold higher than *KRT10* (Figure 7D). Additionally, the basal layer–specific *KRT15* and the granular layer–specific *KRT77* were strongly downregulated in acral skin (Figure 7A and B). Thus, the composition of keratins at acral sites is vastly different from those at nonacral locations.

Apart from characterizing the biogeographic variations in the skin transcriptome and lipidome, our targeted lipid profiling strategy identified several other features of epidermal lipid expression. Specifically, it identified 22,663 statistically significant (FDR < 0.05) lipid-lipid correlations, which followed specific rules (Figure 12C). For example, EOS ceramides and NS ceramides of similar structure positively correlated with one another (e.g., EOS ceramide ω-linoleoyloxy-Cer[d22:1/31:0] positively correlated with NS ceramide Cer[d22:1/27:0]) (Supplemental Figure 13F). We speculate that these correlations, which occur across ceramide subclasses, are likely due to commonalities in ceramide synthetic pathways or precursor analytes. For example, EOS ceramides might be synthesized from NS ceramide–derived metabolites (29), which would explain why they highly correlated with one another. Commonalities in synthetic pathways would also explain why sphingoid base length and FA moiety length are important factors dictating the likelihood that 2 ceramides will correlate with each other (Figure 12B and Supplemental Figure 12D). Indeed, closely related anabolic pathways are likely the reason so many lipid-lipid correlations were observed. These correlative patterns, which have been extensively described here, can help us better understand ceramide synthesis.

However, some structurally dissimilar lipids were also highly correlative. A prime example of this is Chol-SO4, whose expression positively correlated with NS ceramide Cer(d18:1/23:0) (and similar ceramides) and negatively correlated with NH ceramide Cer(t20:1[6OH]/27:0) (and similar ceramides) (Supplemental Figure 13E). Another example is FA 24:1, which tended to negatively correlated with NH(C20), NP(C22) and NS(C22) ceramides and tended to positively correlated with NS(C18) and NDS(C18) ceramides (Figure 12C, Supplemental Figure 13D, and Supplemental Table 9). We created Epilipids (epilipids.com) as an online tool to allow for easy comparison of epidermal lipids, either across different anatomic locations or between different skin diseases.

Given the highly significant correlative relationships among the different epidermal lipids, it is not surprising that strong correlations also existed among ceramide synthase and ELOVL family member genes (Figure 13A), which encode proteins governing key steps in lipid anabolism (Figure 5B and Supplemental Figure 3). Specifically, 2 lipid gene clusters were identified (*ELOVL1*, *ELOVL3*, *ELOVL4*, *ELOVL7*, *CERS3*) and (*ELOVL2*, *ELOVL5*, *ELOVL6*, *CERS2*, *CERS5, CERS6*) (Figure 13A). Interestingly, the expression of the *ELOVL4* was also closely linked to other genes important for skin barrier function and linked to various immune genes (Figure 13, B and C, and Figure 14, A–D). Immune genes associated with *ELOVL4* included *IL18*, *IL36B*, *CDK7*, *PDE4A*, *TLR3*, and *MAP3K8* (Figure 13, Band C, and Figure 14, A–D). It is likely that these genes were not simply coregulated with *ELOVL4*, as the expression of *CDK7*, *PDE4A*, *TLR3*, and *MAP3K8* was altered following *ELOVL4* siRNA knockdown. Also, the expression levels of *IL18*, *IL36B*, *CDK7*, *PDE4A*, *TLR3*, and *MAP3K8* were altered in naturally occurring *ELOVL4* ^lo^ keratinocytes (Figure 14B). Thus, the lipid expression profile of keratinocytes may influence their ability to express autoinflammatory cytokines, including IL-36, an IL-1 family member thought to play a critical role in several autoinflammatory conditions, such as pustular psoriasis (30). This is also relevant because individuals with missense mutations in *ELOLV4* can develop severe erythrokeratoderma (31), a psoriasis-like eruption.

Various disease-associated alterations in epidermal ceramide composition have been previously reported (8, 24). Traditionally, alterations in lipid expression have been reported at the level of ceramide classes. For example, investigators studying psoriasis have demonstrated an increase in AS and NS ceramides and a decrease in EOS, AP, and NP ceramides (8, 24). A separate study positively correlated *staphylococcus* colonization in AD with AS, ADS, NS, and NDS ceramide abundance (11). In contrast to these prior studies, we demonstrate that ceramide sphingoid base length and FA chain length have the strongest influence on the likelihood of a particular ceramide being increased or decreased in psoriasis. For example, there was a marked increase in NS ceramide Cer(d18:1/22:0) and a strong decrease in EOS ceramide ω-linoleoyloxy-Cer(d22:1/31:0) in psoriasis lesional skin (Figure 8, B and C). These findings are consistent with previous reports on NS and EOS ceramides in psoriasis. However, other NS and EOS ceramides did not follow this general rule; NS ceramide Cer(d22:1/26:0) was strongly decreased and EOS ceramide ω-linoleoyloxy-Cer(d18:1/32:0) was strongly increased in psoriasis (Figure 8, B and C). Thus, although a particular subclass of ceramides may appear increased or decreased, the individual constituents of the subclass can behave very differently from one another.

In AD, previous studies have reported increases in AH and AP ceramides and decreases in EOS and EOH ceramides (24). Of the AS ceramides monitored here, AS ceramide Cer(d18:1/22:0[2OH]) expression was noted to be increased in AD (Supplemental Figure 10E), but not all AS ceramides behaved similarly. In fact, various AS, AH, and AP ceramides with 22-carbon sphingoid bases were actually downregulated in AD (Supplemental Figure 10F).

In all cases, altered patterns of lipid expression reported herein were also supported by corresponding transcriptome alterations in lipid gene expression. For example, in psoriasis lesional skin, *SPTLC2* was upregulated (Figure 9A) and *SPTLC3* was downregulated (Supplemental Figure 9A), which would increase the synthesis of ceramides with 18-carbon sphingoid bases and decrease the synthesis of ceramides with long-chain sphingoid bases. This was the observed ceramide profile in psoriasis (Figure 8B). Finally, the increase in expression of *SCD* in lesional psoriasis skin (Supplemental Figure 8) likely accounts for the upregulation of unsaturated FAs (e.g., FA 18:1 and FA 18:2) in psoriasis (Figure 9B).

Given that lipid-associated gene expression appeared to drive the composition of epidermal lipids in psoriasis, we sought to determine how the psoriasis microenvironment might contribute to the changes in lipid-associated gene expression. By characterizing the gene expression of 50 primary human keratinocyte cell lines under different culture conditions, it was apparent that in vitro culture with psoriasis-associated cytokines (TNF and IL-17A) increased the expression of *SPTLC2* (Supplemental Figure 9), which matched the expression pattern of this gene in psoriasis lesional skin (Figure 9A). Likewise, keratinocyte expression of *CERS6* was decreased after incubation with TNF and IL-17A, which also mimicked the downregulation of *CERS6* in psoriasis lesional skin. Thus, psoriasis-associated cytokines may be driving some of the epidermal lipid alterations noted in psoriasis.

Finally, we explored lipid profiling as a diagnostic tool with real-world clinical applications. The shortage of dermatologists outside of major cities and the ease and low cost of tape stripping compared with skin biopsy makes lipid profiling an attractive diagnostic tool. To this end, we identified 77 lipid analytes with utility as diagnostic biomarkers (Supplemental Table 10). We also constructed and cross-validated a highly accurate multianalyte diagnostic classifier for psoriasis (Figure 10E). Widely applicable diagnostic tools such as these are in great need in dermatology, as they may help soften the disparities in dermatologic care experienced by rural and underserved communities.

Although we focused on dermatologic diseases, expanding our biomarker research endeavors to applications outside of dermatology is clearly possible. Serum ceramides are being investigated as potential predictive biomarkers for aging, diabetes, and cardiovascular disease (32–34). Given the correlations between epidermal ceramides and other types of lipids, including cholesterol, we predict that epidermal ceramide profiling will have clinical utility in other fields of medicine, including cardiology. The research presented here also has other implications, especially with respect to designing anatomically specific lipid barrier restoration strategies. For example, the heel of the foot is a site that is often dry and cracked and in need of barrier repair. Similarly, patients with AD have severe barrier dysfunction, especially at specific sites like the AC. Here, we show that each anatomical location has a unique composition of epidermal lipids. Targeted lipid monitoring could serve as an objective measure of skin barrier dysfunction and disease severity, or as a means to categorize patients into disease subtypes. We envision a future in which epidermal profiling will be widely used in dermatologic care.

## Methods

Supplemental Methods are available online with this article.

### Subjects.

All participating subjects were adults recruited from the Department of Dermatology at the UCD. Patients met respective clinical criteria for a diagnosis of psoriasis, AD, AK, SK, or TI. All diagnoses were made by a board-certified dermatologist. The diagnosis of TI was confirmed with potassium hydroxide (KOH) microscopy. Patients were not on any topical or systemic therapy at the time of sampling. For psoriasis and AD samples, lesional and nonlesional anatomically matched paired samples were taken to control for body location. Individuals without underlying dermatologic conditions were used as healthy controls. Normal healthy subjects for acral and nonacral skin sites were recruited from the Department of Dermatology at the University of Michigan. Groups were balanced for sex.

### Data availability.

All transcriptomic data described in this manuscript have been deposited in SRA under the BioProject IDs PRJNA858182, PRJNA858056, and PRJNA742431. All other data and material generated as a part of this work will be made available upon request.

### Statistics.

For full statistical information, see Supplemental Methods.

### Study approval.

The acral biopsy study was approved by the IRB of the University of Michigan. Informed consent was obtained from all participating patients.

## Author contributions

AAM and STL are cofirst authors. Order of authorship was determined by seniority. Conceptualization was contributed by FP and EM. Methodology was contributed by EM, AAM, STL, AT, YX, AIM, SMW, IEA, JW, YI, FTL, BA, LCT, JEG, and CBL. Investigation was contributed by AAM, STL, CA, AT, AIM, SMW, IEA, FP, ACB, YI, A Kumar, RU, JMK, EAW, CM, MYC, HX, A Kirane, GL, LCT, CBL, JEG, and EM. Project administration was contributed by EM. Supervision was contributed by EM, AAM, and SMW. Review and editing of the manuscript was contributed by all authors.

## Supplementary Material

Supplemental data

Supplemental data set 1

Supplemental table 1

Supplemental table 10

Supplemental table 2

Supplemental table 3

Supplemental table 4

Supplemental table 5

Supplemental table 6

Supplemental table 7

Supplemental table 8

Supplemental table 9

## Version 1 07/28/2022

In-Press Preview

## Version 2 08/22/2022

Electronic publication
