# Supplementary material for: Biogeographic and disease-specific alterations in epidermal lipid composition and single-cell analysis of acral keratinocytes
Source: JCI Insight. 2022 Aug 22;7(16):e159762. doi: 10.1172/jci.insight.159762 (PMC9462509; doi:10.1172/jci.insight.159762)
Supplement: Supplemental data [file jciinsight-7-159762-s279.pdf]

SUPPLEMENTAL METHODS

Stratum corneum sampling

Lesional actinic keratosis (AK, n=10), lesional seborrheic keratosis (SK, n=9), lesional tinea corporis (TI, n=3), and healthy control (NN, n=30) skin was obtained. In total, 99 volunteers were recruited for this study and a total of 234 tape stripping samples were analyzed by targeted mass spectrometry. Overall, 351 lipids were monitored in each sample; the subclass structures are shown in Supplemental Figure 1.

Tape strippings of the SC from each patient were taken for lipid analysis. Sample sites for patients with psoriasis included (1) lesional psoriasis skin (PP, n=37) and (2) paired and anatomically matched nonlesional skin (PN, n=16). Sample sites for patients with atopic dermatitis included (1) the lesion with most severe erythema and/or papulation (AD, n=10) and (2) paired anatomically matched (nonlesional) skin (AN, n=9). Generally, patients with actinic keratosis, seborrheic keratosis, and tinea corporis presented with a single lesion, and sample sites were taken from the affected area. Healthy controls had samples from their abdomen (AB), antecubital fossa (AC), alar crease (AL), axilla (AX), cheek (CK), dorsal surface of hand (DH), glabella (GB), popliteal fossa (PF), palmar surface of hand (PH), anterior proximal lower extremity (PLE), upper back (UB), and volar forearm (VF).

Participants were asked to use only mild, unscented soap at least seven days prior to skin sampling. Psoriasis and atopic dermatitis patients that were sampled were not on any systemic immunosuppressant medications and did not apply topical medications for seven days prior to skin tape stripping. For each respective sampling site, adhesive D-SQUAME®

Standard Skin Sampling Discs were placed on the skin with sterile tweezers and gloved hands. Adhesive skin discs were pressed down with gauze for 5 seconds. After another 25 seconds, the tape was gently removed, and transferred to D-SQUAME® Standard Storage Cards. Six discs were applied sequentially to each anatomic site that was sampled. The first two discs were discarded to eliminate superficial corneocytes and sebum, and the subsequent four discs were submitted for analysis. Samples were stored at -80°C.

Lipid extraction and quantification

D-SQUAME disks were extracted sequentially with a polar and a nonpolar organic solvent after addition of a known amount of surrogate standard solution (C16 ceramide-d_{31} (NS ceramideCer(d18:1/16:0))-d_{31}), *cis*-10-heptadecenoic acid (FA17:1n7) and cholesteryl sulfate-d_7). The organic extracts were combined and evaporated to dryness. The dried extract was reconstituted, and an aliquot was analyzed on a Waters UPC2/Sciex QTrap 5500 mass spectrometer SFC-MS/MS system in MRM mode using characteristic parent-fragment mass transitions for each analyte trace. The semi-quantitative determination of individual analytes was based on their peak area compared with the peak areas of their corresponding surrogate standards, for which concentrations were known. C16 ceramide-d_{31} was used as surrogate standard for all ceramides, all fatty acids were referenced to *cis*-10-heptadecenoic acid, and cholesteryl sulfate was referenced to cholesteryl sulfate-d_7. Absolute concentrations of lipids were determined by comparing their peaks to those of relevant internal standards. Concentrations were given in picomol/disk for individual analytes, as well as each lipid class. Additionally, the percent composition of individual ceramide

subtypes of the ceramide fraction was listed for each sample. In total, 351 lipids were simultaneously monitored using this technique.

Nomenclature

Lipid species identified were annotated based on their molecular structure per Motta *et al.*(1) Ceramides were first classified as shown in Supplemental Figure 1. The ceramides were subsequently assigned an analyte designation as follows: [lipid subclass][number of carbons in sphingoid base][number of carbons in fatty acid: number of double bonds in fatty acid]. For example, the analyte AH ceramide Cer(t18:1(6OH)/22:0(2OH)) denotes 6-hydroxysphingosine with an 18 carbon sphingoid base, attached to an α-hydroxy fatty acid with 22 carbons lacking double bonds.

Single-cell RNA-sequencing

Generation of single cell suspensions for single-cell RNA-sequencing (scRNA-seq) was performed as follows: Samples were incubated overnight in 0.4% dispase (Life Technologies) in Hank's Balanced Saline Solution (Gibco) at 4°C. Epidermis and dermis were separated. Epidermis was digested in 0.25% Trypsin-EDTA (Gibco) with 10U/mL DNase I (Thermo Scientific) for 1 hour at 37°C, quenched with FBS (Atlanta Biologicals), and strained through a 70μM mesh. Dermis was minced, digested in 0.2% Collagenase II (Life Technologies) and 0.2% Collagenase V (Sigma) in plain medium for 1.5 hours at 37°C, and strained through a 70μM mesh. Epidermal and dermal cells were recombined and libraries were generated using the 10X Chromium platform. Libraries were then sequenced on the Illumina NovaSeq 6000 sequencer to generate 151-bp paired end reads. Approximately

6,500 median UMI counts per cell were obtained. Data processing including quality control, read alignment, and gene quantification were conducted using 10X Cell Ranger v3.1 using their default parameters. Empty droplets and cells with UMI counts less than a calculated threshold based on the distribution of UMI counts per cell were removed, resulting in elimination of cells with less than approximately 600 UMI. Log normalized data and ANOVA was used to calculate differential expression among cell types. Data normalization and cell library size correction was performed using the median of ratios method implemented in the R package DESeq2. Clustered cells were mapped to corresponding cell types using "singleR" R package and the Human Primary Cell Atlas as a reference data. Keratinocytes were then further subdivided into basal, spinous, and granular layer keratinocytes by matching their cell cluster gene signatures with putative cell-type specific markers (*DST*, *KRT5*, *KRT10*, and *KLK7*).

RNA-sequencing (RNA-seq)

Skin biopsies of 4 mm in diameter were taken, snap frozen in liquid nitrogen, and subsequently stored at –80°C until further processing. RNA isolation and sequencing was performed as previously described by our group (2). The paired-end reads were mapped using STAR (3) to human build GRCh37, and only uniquely mapped reads were utilized for subsequent analysis. Raw whole tissue RNA-sequencing FASTQ files were obtained from previously published studies (4). We obtained 20 million reads per sample on average and approximately 86% of the reads were uniquely mapped and used for the downstream analysis." Gene expression levels were quantified (GENCODE v24 was used as reference) and normalized by HTSeq(5) and DESeq2 (6), respectively. Negative binomial model in

DESeq2 were used to conduct differential expression analysis. The expression of 83 genes (Supplemental Table 3) that have been reported in the literature to be involved in epidermal lipid metabolism was assessed in biopsy specimens obtained from acral and trunk skin. Differentially expressed lipid genes across these two anatomic locations were defined as those with a fold change of 2 or greater and an FDR adjusted p value of less than 0.05.

Statistical analysis

All statistical analysis were performed using R software (7). Prior to statistical analysis, samples with values below Limit of Detection (LOD) were assigned a concentration equal to the LOD divided by the square root of two. There were otherwise no missing data. The assumptions of homoscedasticity and heteroscedasticity were evaluated, and non-normal data was transformed using the BoxCox method. Outliers were identified using R package "extremevalues" (8), and when present, were winsorized from the analysis, so that outliers were set equal to the nearest non-outlier value. Following normalization and transformation, linear mixed-effects model was used to identify lipids with body site-specific expression. FDR-adjusted p-values were calculated using the Benjamini-Hochberg procedure.

Differentially expressed lipids amongst diagnostic groups (AD, PP, SK, AK, and TI) were identified using ANOVA. Heatmaps comparing differential expression of lipids were generated with the R package "pheatmap"(9).

Receiver-operating characteristic (ROC) curves were generated and area under the curves (AUC) were calculated for individual SC lipids that demonstrated diagnostic accuracy as

single analyte classifiers of psoriasis or atopic dermatitis using the R package "ROCR"(10). Step forward logistic regression models were fitted using Firth's bias reduction method with the R package "logistf"(11). Models were validated using the K-fold cross validation method. ROC curves and AUC values for these models were calculated as just described. Linear regression assumptions about the normality of residuals were examined by use of the Shapiro-Wilk test.

Relatedness between different body groups and between diagnostic groups was determined by calculating the Euclidian Distance as follows: simple matching distance (SMD) was calculated for each respective analyte across the two diagnostic groups and separately for two body groups (for example, between PP and PN or between PF and VF, respectively). The process was repeated for all 351 analytes and across each pair of diagnostic groups and pair of body groups. SMD of each of the analytes were squared and summed, and the square root was obtained. A cluster dendrogram was created based on these values. With this data, a PCA was also created, and data for the PCA was scaled and centered.

Product-moment correlation coefficients (PMCCs) were calculated and graphed for each lipid-lipid combination to show relationships of co-expression between specific lipids. A 2-dimensional visual representation of all lipid-lipid correlations was created using a dimensionality reduction technique, t-distributed stochastic neighbor embedding (t-SNE), and calculated with the R package "Rtsne"(12), using the pairwise distance formula, $1-r^2$, where r represents the correlation coefficient.

References for Supplemental Methods

1. Motta S, Monti M, Sesana S, Caputo R, Carelli S, and Ghidoni R. Ceramide composition of the psoriatic scale. *Biochim Biophys Acta.* 1993;1182(2):147-51.
2. Liang Y, Tsoi LC, Xing X, Beamer MA, Swindell WR, Sarkar MK, et al. A gene network regulated by the transcription factor VGLL3 as a promoter of sex-biased autoimmune diseases. *Nat Immunol.* 2017;18(2):152-60.
3. Dobin A, Davis CA, Schlesinger F, Drenkow J, Zaleski C, Jha S, et al. STAR: ultrafast universal RNA-seq aligner. *Bioinformatics.* 2013;29(1):15-21.
4. Tsoi LC, Rodriguez E, Degenhardt F, Baurecht H, Wehkamp U, Volks N, et al. Atopic dermatitis is an IL-13 dominant disease with greater molecular heterogeneity compared to psoriasis. *J Invest Dermatol.* 2019.
5. Anders S, Pyl PT, and Huber W. HTSeq--a Python framework to work with high-throughput sequencing data. *Bioinformatics.* 2015;31(2):166-9.
6. Love MI, Huber W, and Anders S. Moderated estimation of fold change and dispersion for RNA-seq data with DESeq2. *Genome Biol.* 2014;15(12):550.
7. Team RC. A language and environment for statistical computing. R Foundation for Statistical Computing, Vienna, Austria. Available online at https://www.R-project.org/. 2018.
8. van der Loo MPJ. Extremevalues, an R package for outlier detection in univariate data. R package version 2.1. . http://CRAN.R-project.org/package=extremevalues.
9. Kolde R. pheatmap: Pretty Heatmaps. R package version 1.0.10. https://CRAN.R-project.org/package=pheatmap. 2018.
10. Sing T, Sander O, Beerenwinkel N, and Lengauer T. ROCR: visualizing classifier performance in R. *Bioinformatics.* 2005;21(20):3940-1.
11. Heinze G, and Puhr R. Bias-reduced and separation-proof conditional logistic regression with small or sparse data sets. *Stat Med.* 2010;29(7-8):770-7.
12. JH. K. Rtsne: T-Distributed Stochastic Neighbor Embedding using a Barnes-Hut Implementation. URL: https://github.com/jkrijthe/Rtsne. 2015.

Supplemental Figure Legends

Supplemental Figure 1. Ceramide structures. Ceramides are composed of an amino alcohol (e.g. sphingosine) and a fatty acid, which may have an alpha hydroxyl group. Shown here are the epidermal ceramide classes that were monitored by targeted mass spectrometry. An "A" (ADS, AH, AP, and AS) specifies an alpha-hydroxy fatty acid.

Supplemental Figure 2. Plantar heel skin has a unique pattern of epidermal lipid expression. (**A**) Box-and-whisker plots of the relative abundance of representative epidermal ceramides that are differentially expressed in PH (red arrow) when compared to other anatomical locations. The upper and lower bars connected to each box indicate the boundaries of the normal distribution, and the box edges mark the first and third quartile boundaries within each distribution. The dark vertical line represents the median. (**B**) As a general rule, ceramides comprised of an 18 carbon sphingoid base and a fatty acid 22 carbons in length are increased in PH epidermis. Also shown are ceramides with 23 carbon fatty acids. This pattern of ceramide expression matches the differential expression of lipid genes in PH skin (Figure 3).

Supplemental Figure 3. PH-associated changes in lipid metabolism. (**A**) Metabolic pathways to synthesize new lipids from smaller constituent molecules are shown, as well as some mitochondrial lipid metabolic enzymes. Red arrows indicate increased expression in PH epidermis, based on RNA-seq and single-cell RNA-seq data. Blue arrows indicate decreased expression. C18 ceramides appear to be favored over C16 ceramides due to upregulation of *SPTLC2* and downregulation of *SPTLC3* in PH skin. PH skin is also associated with a large increase in *CERS3* and *CERS4* (**B**) Ceramide salvage pathway.

Supplemental Figure 4. Single-cell RNA-sequencing of palm and trunk epidermis reveals altered expression of lipid-associated metabolic genes. Punch biopsies were obtained from the palm and trunk skin. Cell suspensions of the epidermis were then prepared, and single cells were sequenced using the 10X Genomics platform. (**A**) Granular layer cells were identified by expression of granular layer-specific genes (e.g. KLK7). Results are presented as box-and-whisker plots, where the upper and lower bars connected to each box indicate the boundaries of the normal distribution, and the box edges mark the first and third quartile boundaries within each distribution. Each dot represents an individual single keratinocyte cell. (**B**) Single cell keratinocyte transcriptome data is presented using the uniform manifold approximation and projection (UMAP) method. Each dot represents an individual keratinocyte. Note that keratinocytes originating from the same anatomic location and epidermal layer cluster together (basal [palm=green, trunk=yellow], spinous [palm=blue, trunk=pink], and granular [palm=red, trunk=orange] layers). Cells expressing the noted lipid-associated metabolic gene are depicted in purple. P-values are provided for genes differentially expressed in palm versus trunk epidermis. (**C**) Bulk RNA-seq was performed on plantar foot and trunk skin biopsies, and differentially expressed genes (fold change > 2 and FDR-adjusted p values < 0.05) were identified. Representative genes are plotted as box and whisker plots.

Supplemental Figure 5. Transcriptome alterations in acral keratinocytes. (**A**) Single-cell sequencing was performed on keratinocytes isolated from paired palm and trunk skin biopsies. Results are presented as box-and-whisker plots. Each individual data point represents the number of reads that mapped to the indicated gene in a single keratinocyte at the specified layer of the epidermis. The upper and lower bars connected to each box indicate the boundaries of the normal distribution, and the box edges mark the first and third quartile boundaries within each distribution. The dark vertical line represents the median. (**B**) Heatmap summarizing expression of keratin genes in trunk and acral skin, which demonstrates that plantar and palmar skin have similar expression of keratin genes. (**C**) Paired biopsies obtained from palm and trunk skin were evaluated by whole tissue RNA-seq. Differentially expressed keratin genes are presented as box-and-whisker plots.

Supplemental Figure 6. Transcriptome and lipid alterations in acral skin.

Supplemental Figure 7. Alterations of epidermal lipid expression in psoriasis. Tape stripping was performed to sample epidermal lipids of psoriasis lesional (PP), paired non-lesional (PN), and healthy control (NN) skin. Lipids were then quantified by targeted mass spectrometry. Upper row: Box-and-whisker plots of the epidermal ceramides that are differentially expressed in PP skin compared to PN skin. Lower row: Box-and-whisker plots of the epidermal ceramides that are differentially expressed in PP skin compared to NN skin. (**A**) As a general rule, ceramides with an 18-carbon sphingoid base and a fatty acid moiety 22 or 24 carbons in length were significantly upregulated in PP skin. (**B**) Psoriasis skin is also hallmarked by downregulation of ceramides with 22 carbon sphingoid base and fatty acids 26 carbons in length. (**C**) Acyl-ceramides are variably expressed in psoriasis, with some being consistently upregulated and others being downregulated. EOS ceramides with 18 carbon sphingoid bases were more likely to be upregulated. (**D**) Additional examples of epidermal lipids differentially expressed in different dermatologic diseases as determined by targeted mass spectrometry. Representative lipids were chosen to highlight the characteristic patterns of lipid expression in lesional psoriasis epidermis. The upper and lower bars connected to each box indicate the boundaries of the normal distribution, and the box edges mark the first and third quartile boundaries within each distribution. The dark vertical line represents the median.

Supplemental Figure 8. Differential expression of lipid-associated metabolic genes in psoriasis. RNA-seq was performed on RNA extracted from biopsies obtained from psoriasis lesional skin and healthy controls. Expression of lipid-associated metabolic genes is shown as box-and-whisker plots. The upper and lower bars connected to each box indicate the boundaries of the normal distribution, and the box edges mark the first and third quartile boundaries within each distribution. The dark vertical line represents the median. The unpaired Student's t-test was used to calculate significance.

Supplemental Figure 9. Cytokine-induced differential expression of lipid-associated metabolic genes predict lipid alterations in psoriasis. (**A**) RNA-seq analysis was performed on biopsies obtained from psoriasis lesional (PP), atopic dermatitis lesional (AD) and healthy control skin (NN). Differential expression of representative genes involved in

ceramide synthesis are shown. (**B**) 50 human primary keratinocyte cell lines were cultured with indicated cytokines (including the psoriasis-associated cytokines IFN-γ, IL-17A, TNF)(10). RNA was then extracted and gene expression was evaluated with RNA-seq. Results demonstrate that *in vitro* culture with TNF increases the expression of *SPTLC1*, *SPTLC2* and decreases the expression of *SPTLC3*. Also, the expression of *CERS6* is decreased by IFN-γ and TNF. These cytokine-induced alterations in gene expression mimic the altered gene expression in psoriasis lesional skin.

Supplemental Figure 10. Transcriptome and lipid alterations in atopic dermatitis lesional skin. (**A**) Principal component analysis of lipid-associated metabolic gene expression data reveals near complete separation of samples by diagnostic group (red=atopic dermatitis lesional skin, grey=healthy control skin). (**B**) Box-and-whisker plots of lipid-associated metabolic genes expression in atopic dermatitis versus healthy control skin. (**C**) CERS proteins synthesize ceramides. Predicted alterations in ceramide expression based on the differential expression of *CERS* genes in atopic dermatitis lesional skin. Red indicates upregulation of *CERS* gene expression in atopic dermatitis and blue indicates downregulation of *CERS* expression. (**D**) When compared to psoriasis lesional skin, AD lesional skin has higher expression of 22 carbon sphingoid base ceramides and fatty acid moieties 26 carbons in length. A finding that was expected from the upregulation of *CERS2* in AD lesional skin but not psoriasis lesional skin. Furthermore, while the differential expression of *SPTLC2* and *SPTLC3* in AD has the same directionality as in psoriasis lesional skin, the fold change in gene expression is lower, which would favor a higher expression of ceramides with 22 carbon sphingoid bases in AD versus psoriasis lesional skin. (**E**) As a general rule, when compared to non-lesional atopic dermatitis skin (AN) and healthy control skin (NN), AD lesional skin has higher expression of ceramides with 18 carbon sphingoid bases and fatty acid moieties 22 carbons in length. (**F**) As a general rule, when compared to AN or NN skin, 22 carbon sphingoid base ceramides are decreased in AD skin.

Supplemental Figure 11. Single analyte lipid classifiers can distinguish dermatologic diseases from one another. (**A**) Receiver-operating characteristic (ROC) curves are presented for single analyte diagnostic lipid classifiers capable of distinguishing psoriasis lesional skin (PP) from all other diagnostic groups (NN, PN, PP, AK, SK, TI, and AD) combined. Areas under the receiver operator curves (AUC) are shown. Psoriasis lipid classifiers were chosen from different ceramide subclasses to demonstrate the broad array of different lipids that have diagnostic utility in psoriasis. (**B**) ROC curves of single analyte diagnostic lipid classifiers are presented for normal skin and lesional skin of atopic dermatitis, actinic keratosis, seborrheic keratosis, and tinea corporis. Diagnostic classifiers can distinguish the respective diagnostic group from all other diagnostic groups combined. AUCs are shown.

Supplemental Figure 12. General rules governing lipid-lipid correlations among epidermal lipids. (**A**) The t-distributed stochastic neighbor embedding (t-SNE) dimensionality reduction technique was used to visualize the 123,201 lipid-lipid correlations as a 2-dimensional image. Each dot represents a monitored lipid analyte. The color of the dot represents the lipid class that the analyte belongs to: ADS (red), AH (orange), AP (yellow), AS (lime), cholesterol sulfate (green), EOH (light green), EOS (light blue), free

fatty acid (blue), NDS (dark blue), NH (purple), NP (magenta), NS (fuchsia) (lipid structures are shown in Figure S1). The resulting t-SNE clusters demonstrate that 1) not all lipids of the same class or subclass correlate with one another, and 2) lipids of different classes and different subclasses can correlate with one another. (**B**) Bar graphs illustrate the percent of ceramides that correlated with each other. Upper graph illustrates the negative correlative relationship between 18 carbon sphingoid base ceramides and 22 carbon sphingoid base ceramides. Lower graph illustrates the positive correlative relationship between the NS and NDS ceramides with unsaturated FA24:1 and the negative correlative relationship these ceramides have with the saturated FA24:0. (**C**) Correlation matrix representing the patterns of negative correlations among different lipid subclasses. The intensity of the color at the intersect between a column and row represents the average correlation coefficient for that particular lipid subclass combination. The size of the circle within each colored box represents the percent of lipids that negatively correlated. Hierarchical clustering is used to order the lipid subclasses based on their patterns of correlation. From this correlation matrix, it is evident that cholesterol sulfate has a strong negative correlation with many AH and NH ceramides. (**D**) Ceramides were grouped by the length of their sphingoid bases. Lipid expression across different groups was then assessed and a correlation matrix constructed. The intensity of the color at the intersect between a column and row represents the average negative correlation coefficient for that particular group comparison. The size of the circle within each colored box represents the percent of lipids that correlated. From this correlation matrix, it is evident that 18 carbon sphingoid base ceramides tended to negatively correlate with 22 carbon sphingoid base ceramides. Furthermore, cholesterol sulfate tends to negatively correlate with 26 carbon sphingoid base ceramides (**E**) Correlation matrix displaying positive correlations between lipid classes. The strongest positive correlations are between lipids of the same class. Also, cholesterol sulfate tends to positively correlate with AS, EOS, and NS ceramides. (**F**) Correlation matrix displaying negative correlations between lipid classes.

Supplemental Figure 13. Patterns of lipid expression in the epidermis. Epidermal lipids were sampled using the tape stripping technique and individual lipids were quantified by targeted mass spectrometry. Scatter plots were constructed to compare the abundance of different lipids to one another. Several general overarching trends in lipid expression could be observed. (**A**) Inter-class ceramides with sphingoid base and fatty acid moieties of identical length correlate strongly with one another; shown here, NH ceramide Cer(t18:1(6OH)/30:0) positively correlated with NS ceramide Cer(d18:1/30:0) (r=0.94, FDR=3.0e-49). (**B**) 18 carbon sphingoid base ceramides (C18) negatively correlate with 20 and 22 carbon sphingoid base ceramides, usually with dissimilar length fatty acids. The strongest negative correlations are between AH and AS C18 ceramides and NP and NH C20 and C22 ceramides. Shown here, AH ceramide Cer(t18:1(6OH)/20:0(2OH)) negatively correlated with NP ceramide Cer(t22:0/26:0) (r=-0.93, FDR=1.5e-46). Additionally, NS C18 ceramides negatively correlated with NP(C22) ceramides; shown here, NS ceramide Cer(d18:1/23:0) negatively correlated with NP ceramide Cer(t22:0/25:0); r=-0.88, FDR=6.7e-34). (**C**) Saturated fatty acids of similar length positively correlated with one another; shown here, FA16:0 positively correlated with FA18:0 (r=0.94, FDR=4.9e-49). (**D**) FA24:1 negatively correlated with 20 and 22 carbon sphingoid base NH and NP ceramides, shown here FA24:1 negatively correlated with NH ceramide Cer(t20:1(6OH)/27:0); (r= 0.81,

FDR=2.97e-23) and NP ceramide Cer(t22:0/26:0) (r=-0.80, FDR=3.96e-22). (**E**) Chol-SO4 positively correlated with C18 ceramides, especially 18 carbon sphingoid base NS ceramides, shown here Chol-SO4 positively correlated with NS ceramide Cer(d18:1/23:0) (r=0.80, FDR=3.7e-22). In contrast, Chol-SO4 negatively correlated with NH C20 and C22 ceramides, C26 NDS ceramides, and C22 NP ceramides; shown here, Chol-SO4 negatively correlated with NH ceramide Cer(t20:1(6OH)/27:0) (r=-0.78, FDR=1.4e-19). (**F**) EOH ceramides positively correlated with their counterpart EOS ceramides. Shown here, EOH ceramide ω-linoleoyloxy-Cer(t20:1(6OH)/32:0) positively correlated with EOS ceramide ω-linoleoyloxy-Cer(d20:1/32:0) (r=0.83, FDR=7.5e-26). They also correlated with ceramides of similar structure within their own subclass; shown here, EOS ceramide ω-linoleoyloxy-Cer(d20:1/29:0) positively correlated with EOS ceramide ω-linoleoyloxy-Cer(d20:1/31:0) (r=0.93, FDR=2.1e-45). However, in comparison to EOH ceramides, EOS ceramides more strongly correlated with NS ceramides of similar structure; shown here, EOS ceramide ω-linoleoyloxy-Cer(d22:1/31:0) positively correlated with NS ceramide Cer(d22:1/27:0); r=0.88, FDR=2.8e-33).

Supplemental Figure 14. Altered gene expression in *ELOVL4*^{low}-expressing keratinocytes. Keratinocyte RNA-seq datasets were parsed into three groups based on the *ELOVL4* allele they expressed (0/0 representing the *ELOVL4* reference allele and 0/1 and 1/1 representing heterozygosity and homozygosity for the *ELOVL4* variant, rs62407622). Keratinocytes homozygous for rs62407622 expressed significantly lower levels of *ELOVL4*. The same observation was noted when psoriasis patients were parsed for the same allele; rs62407622 homozygosity was associated with low *ELOLV4* expression in psoriasis lesional skin. Keratinocytes homozygous for the ELOVL4^{low} variant also expressed significantly lower levels of the lipid genes, *CERS3* and *SPTLC3*; the keratin genes, *KRT77* and *KRT10*; the skin barrier genes, *CSTA*, *KLK7*, *PDAI1*, and *TGM5*; and lower levels of *IL18*, an IL1-family member cytokine. Also shown are scatter plots of keratinocyte gene expression (primary keratinocyte cell lines). There was a significant positive correlation between *ELOVL4* and each of the aforementioned genes.

Supplemental Tables

Supplemental Table 1. Targeted quantification of epidermal lipids across different biogeographic regions.

Supplemental Table 2. Mixed-effects model of epidermal lipid expression.

Supplemental Table 3. RNA-seq analysis of lipid associated metabolic genes in palm versus trunk skin.

Supplemental Table 4. Targeted quantification of epidermal lipids in psoriasis, atopic dermatitis, and other skin diseases.

Supplemental Table 5. Lipid expression in lesional versus non-lesional psoriasis skin.

Supplemental Table 6. Lipid expression in psoriasis lesional skin compared to other skin diseases.

Supplemental Table 7. Lipid expression in atopic dermatitis lesional skin compared to atopic dermatitis non-lesional skin.

Supplemental Table 8. Differential lipid expression between atopic dermatitis and psoriasis lesional skin.

Supplemental Table 9. Lipid-lipid expression comparisons- Pearson correlation coefficients.

Supplemental Table 10. Performance of lipid analytes as diagnostic classifiers.

Dihydroceramide ADS

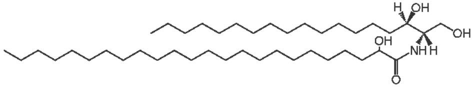

Cer(d18:0/24:0(2OH))

Cholesterol Sulfate

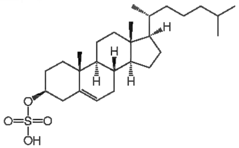

Dihydroceramide NDS

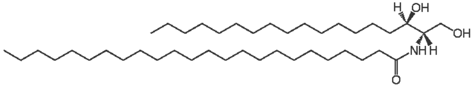

Cer(d18:0/24:0)

6-hydroxyceramide AH

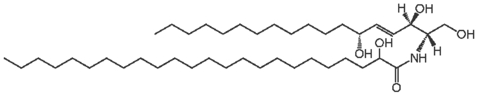

Cer(t18:1(6OH)/22:0(2OH))

6-hydroxyceramide EOH

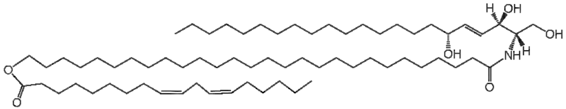

ω-linoleoyloxy-Cer(t18:1(6OH)/31:0)

6-hydroxyceramide NH

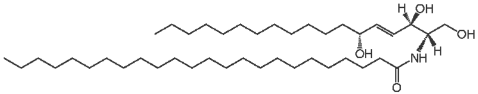

Cer(t18:1(6OH)/24:0)

Phytoceramide AP

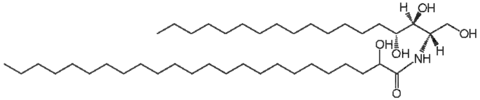

Cer(t18:0/24:0(2OH))

Free Fatty Acids

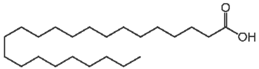

C23:0
Tricosylic acid

Phytoceramide NP

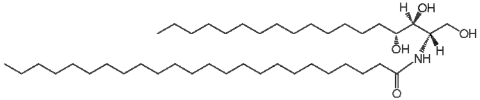

Cer(t18:0/24:0)

Ceramide AS

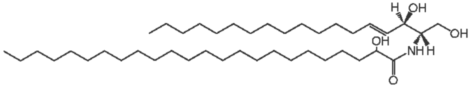

Cer(d18:1(4E)/24:0(2OH))

Ceramide EOS

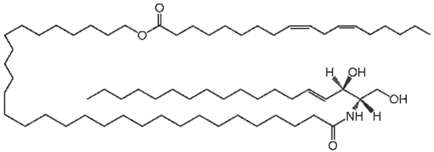

ω-linoleoyloxy-Cer(d18:1/30:0)

Ceramide NS

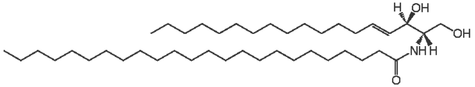

Cer(d18:1/24:0)

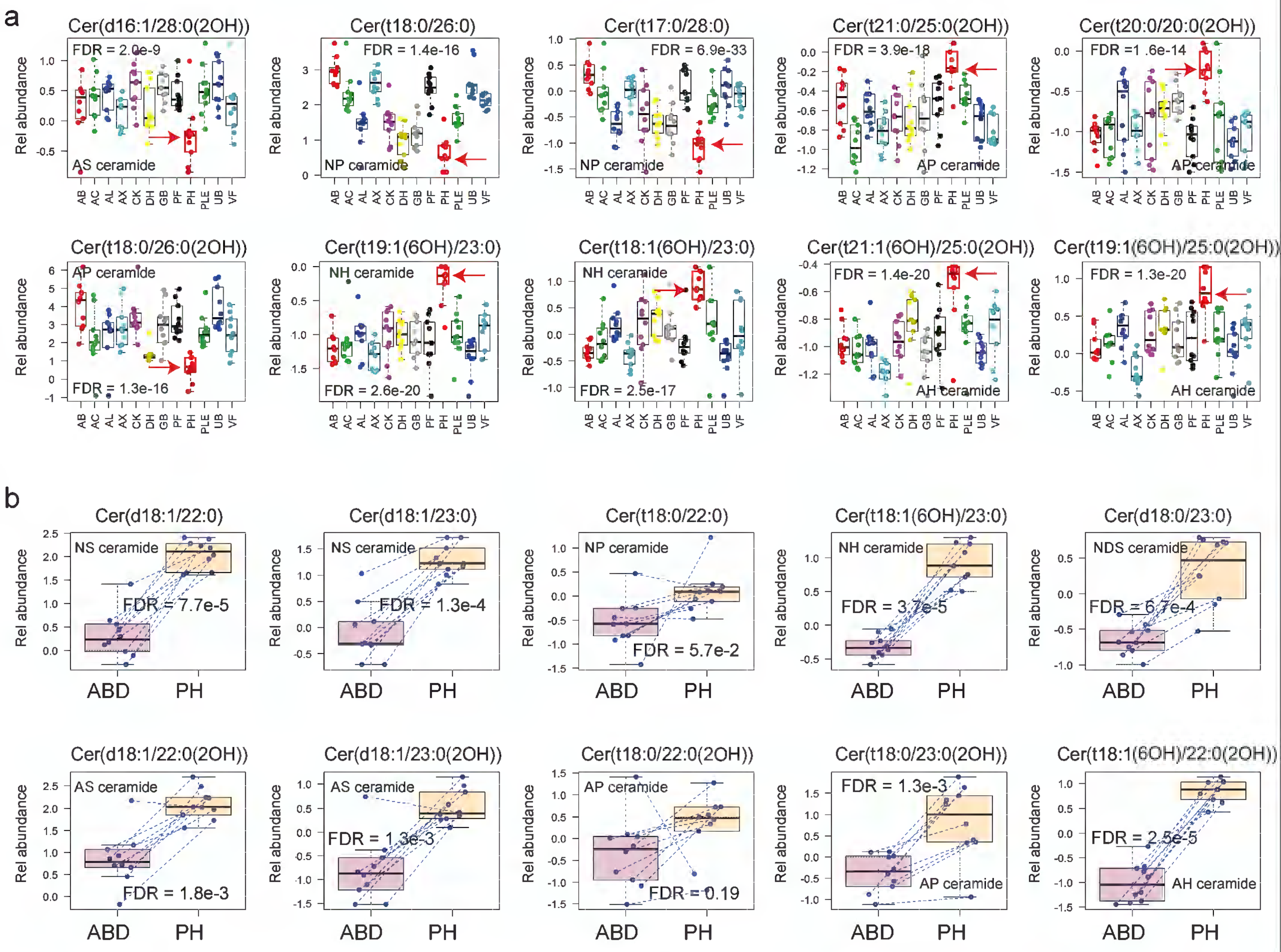
a
Cer(d16:1/28:0(2OH))
FDR = 2.0e-9
AS ceramide
Cer(t18:0/26:0)
FDR = 1.4e-16
NP ceramide
Cer(t17:0/28:0)
FDR = 6.9e-33
NP ceramide
Cer(t21:0/25:0(2OH))
FDR = 3.9e-18
AP ceramide
Cer(t20:0/20:0(2OH))
FDR =1 .6e-14
AP ceramide
Cer(t18:0/26:0(2OH))
AP ceramide
FDR = 1.3e-16
Cer(t19:1(6OH)/23:0)
NH ceramide
FDR = 2.6e-20
Cer(t18:1(6OH)/23:0)
NH ceramide
FDR = 2.5e-17
Cer(t21:1(6OH)/25:0(2OH))
FDR = 1.4e-20
AH ceramide
Cer(t19:1(6OH)/25:0(2OH))
FDR = 1.3e-20
AH ceramide
Rel abundance
AB AC AL AX CK DH GB PF PH PLE UB VF
b
Cer(d18:1/22:0)
NS ceramide
FDR = 7.7e-5
Cer(d18:1/23:0)
NS ceramide
FDR = 1.3e-4
Cer(t18:0/22:0)
NP ceramide
FDR = 5.7e-2
Cer(t18:1(6OH)/23:0)
NH ceramide
FDR = 3.7e-5
Cer(d18:0/23:0)
NDS ceramide
FDR = 6.7e-4
Cer(d18:1/22:0(2OH))
AS ceramide
FDR = 1.8e-3
Cer(d18:1/23:0(2OH))
AS ceramide
FDR = 1.3e-3
Cer(t18:0/22:0(2OH))
AP ceramide
FDR = 0.19
Cer(t18:0/23:0(2OH))
FDR = 1.3e-3
AP ceramide
Cer(t18:1(6OH)/22:0(2OH))
FDR = 2.5e-5
AH ceramide
Rel abundance
ABD
PH

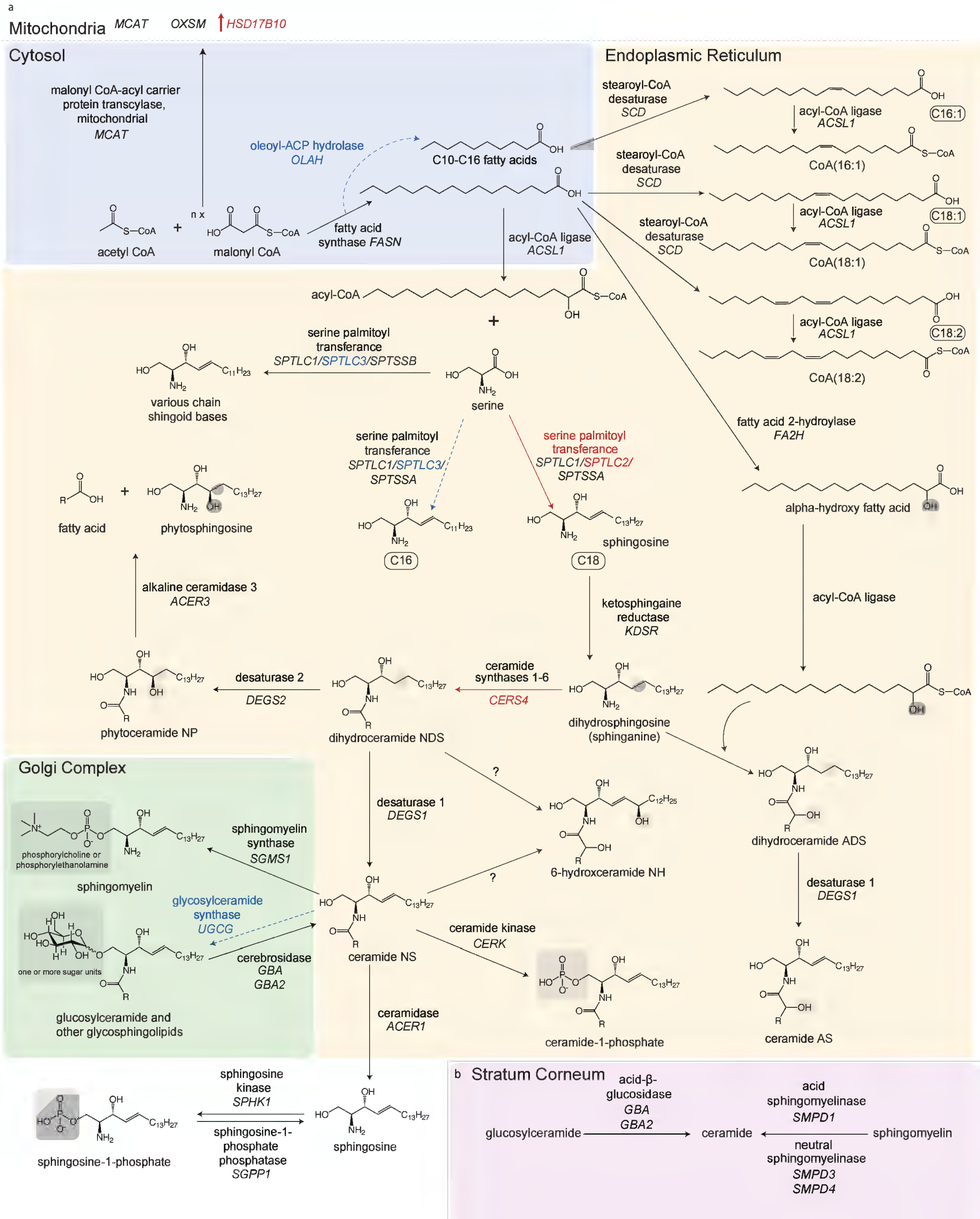
a
Mitochondria MCAT OXSM HSD17B10
Cytosol
malonyl CoA-acyl carrier protein transcylase, mitochondrial MCAT
oleoyl-ACP hydrolase OLAH
C10-C16 fatty acids
acetyl CoA
malonyl CoA
fatty acid synthase FASN
acyl-CoA ligase ACSL1
Endoplasmic Reticulum
stearoyl-CoA desaturase SCD
acyl-CoA ligase ACSL1
C16:1
CoA(16:1)
C18:1
CoA(18:1)
C18:2
CoA(18:2)
acyl-CoA
serine palmitoyl transferance SPTLC1/SPTLC3/SPTSSB
various chain shingoid bases
serine
serine palmitoyl transferance SPTLC1/SPTLC3/ SPTSSA
serine palmitoyl transferance SPTLC1/SPTLC2/ SPTSSA
fatty acid 2-hydroylase FA2H
alpha-hydroxy fatty acid
fatty acid
phytosphingosine
C16
C18
sphingosine
alkaline ceramidase 3 ACER3
ketosphingaine reductase KDSR
acyl-CoA ligase
desaturase 2 DEGS2
ceramide synthases 1-6 CERS4
phytoceramide NP
dihydroceramide NDS
dihydrosphingosine (sphinganine)
Golgi Complex
desaturase 1 DEGS1
dihydroceramide ADS
6-hydroxceramide NH
sphingomyelin synthase SGMS1
phosphorylcholine or phosphorylethanolamine
sphingomyelin
glycosylceramide synthase UGCG
cerebrosidase GBA GBA2
one or more sugar units
glucosylceramide and other glycosphingolipids
ceramide NS
ceramide kinase CERK
ceramide-1-phosphate
ceramide AS
ceramidase ACER1
sphingosine kinase SPHK1
sphingosine-1-phosphate phosphatase SGPP1
sphingosine-1-phosphate
sphingosine
b Stratum Corneum
acid-β-glucosidase GBA GBA2
glucosylceramide
ceramide
acid sphingomyelinase SMPD1
neutral sphingomyelinase SMPD3 SMPD4
sphingomyelin

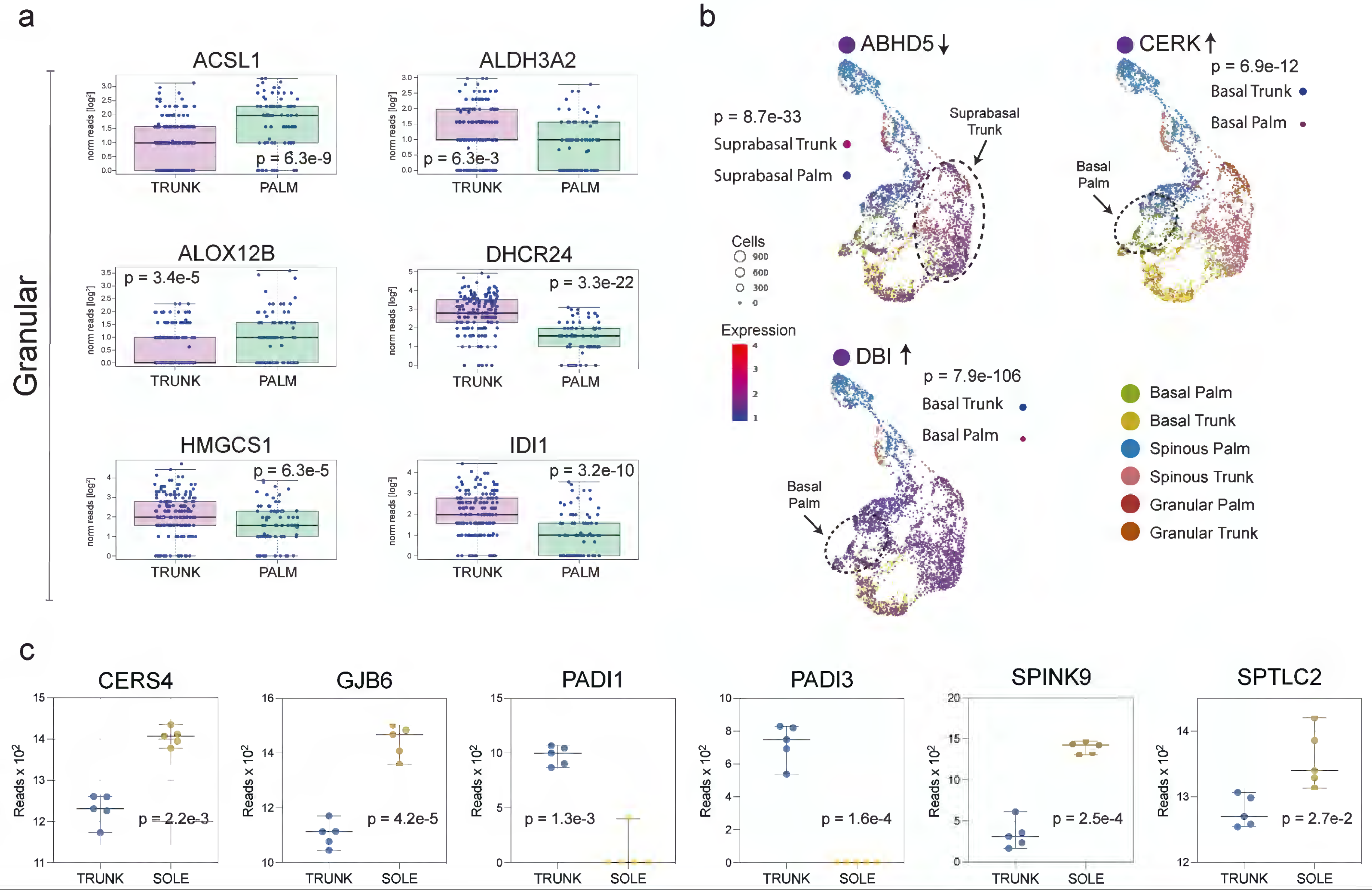

a
Granular
ACSL1
p = 6.3e-9
ALDH3A2
p = 6.3e-3
ALOX12B
p = 3.4e-5
DHCR24
p = 3.3e-22
HMGCS1
p = 6.3e-5
IDI1
p = 3.2e-10
norm reads [log2]
TRUNK
PALM
b
ABHD5 ↓
p = 8.7e-33
Suprabasal Trunk
Suprabasal Palm
Suprabasal Trunk
CERK ↑
p = 6.9e-12
Basal Trunk
Basal Palm
Basal Palm
Cells
900
600
300
0
Expression
DBI ↑
p = 7.9e-106
Basal Trunk
Basal Palm
Basal Palm
Basal Palm
Basal Trunk
Spinous Palm
Spinous Trunk
Granular Palm
Granular Trunk
c
CERS4
p = 2.2e-3
GJB6
p = 4.2e-5
PADI1
p = 1.3e-3
PADI3
p = 1.6e-4
SPINK9
p = 2.5e-4
SPTLC2
p = 2.7e-2
Reads x 10²
TRUNK
SOLE

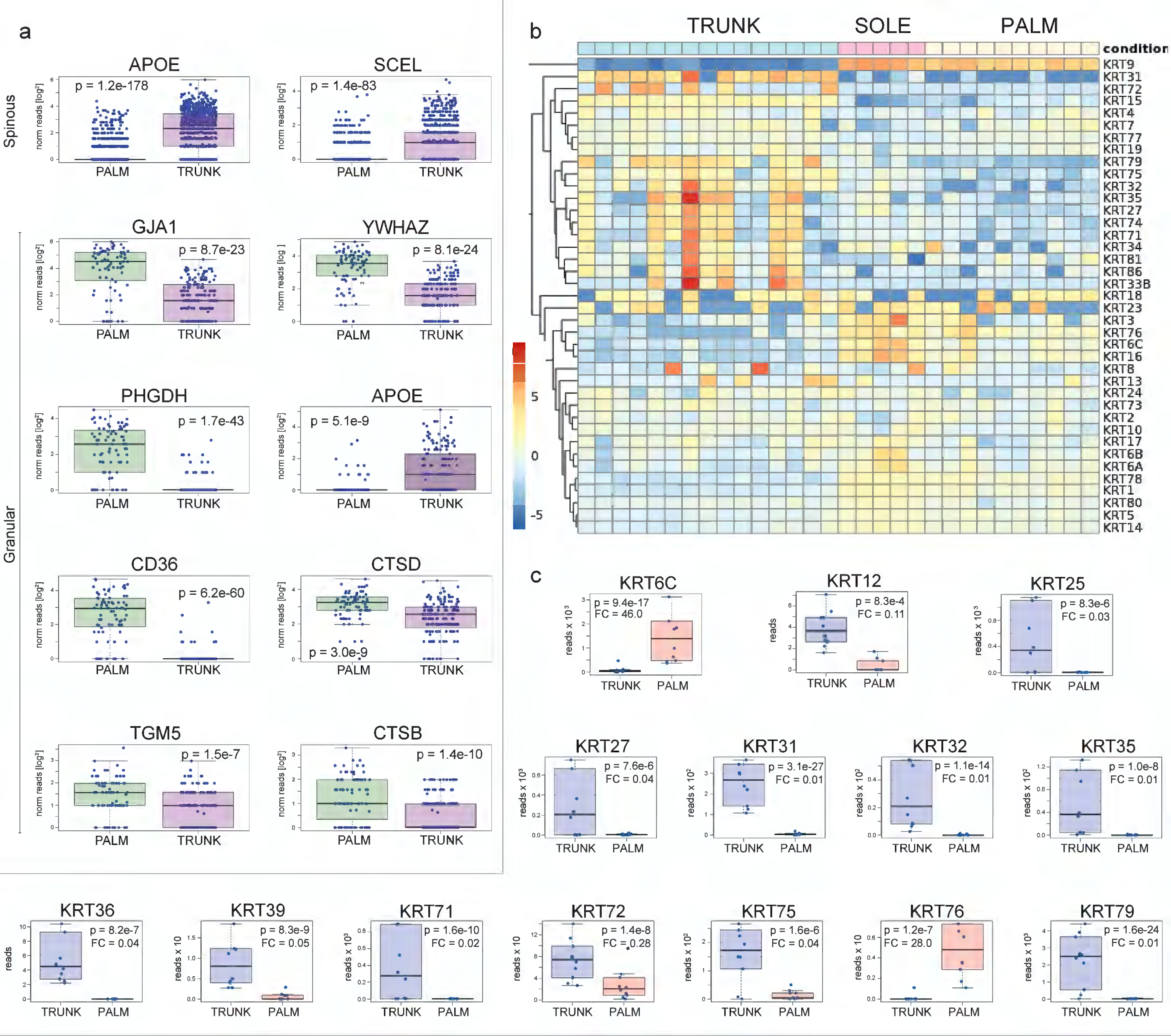
a
Spinous
Granular
APOE
p = 1.2e-178
SCEL
p = 1.4e-83
GJA1
p = 8.7e-23
YWHAZ
p = 8.1e-24
PHGDH
p = 1.7e-43
APOE
p = 5.1e-9
CD36
p = 6.2e-60
CTSD
p = 3.0e-9
TGM5
p = 1.5e-7
CTSB
p = 1.4e-10
norm reads [log²]
PALM
TRUNK
b
TRUNK
SOLE
PALM
condition
KRT9
KRT31
KRT72
KRT15
KRT4
KRT7
KRT77
KRT19
KRT79
KRT75
KRT32
KRT35
KRT27
KRT74
KRT71
KRT34
KRT81
KRT86
KRT33B
KRT18
KRT23
KRT3
KRT76
KRT6C
KRT16
KRT8
KRT13
KRT24
KRT73
KRT2
KRT10
KRT17
KRT6B
KRT6A
KRT78
KRT1
KRT80
KRT5
KRT14
5
0
-5
c
KRT6C
p = 9.4e-17
FC = 46.0
KRT12
p = 8.3e-4
FC = 0.11
KRT25
p = 8.3e-6
FC = 0.03
KRT27
p = 7.6e-6
FC = 0.04
KRT31
p = 3.1e-27
FC = 0.01
KRT32
p = 1.1e-14
FC = 0.01
KRT35
p = 1.0e-8
FC = 0.01
KRT36
p = 8.2e-7
FC = 0.04
KRT39
p = 8.3e-9
FC = 0.05
KRT71
p = 1.6e-10
FC = 0.02
KRT72
p = 1.4e-8
FC = 0.28
KRT75
p = 1.6e-6
FC = 0.04
KRT76
p = 1.2e-7
FC = 28.0
KRT79
p = 1.6e-24
FC = 0.01
reads
reads x 10
reads x 10²
reads x 10³
TRUNK
PALM

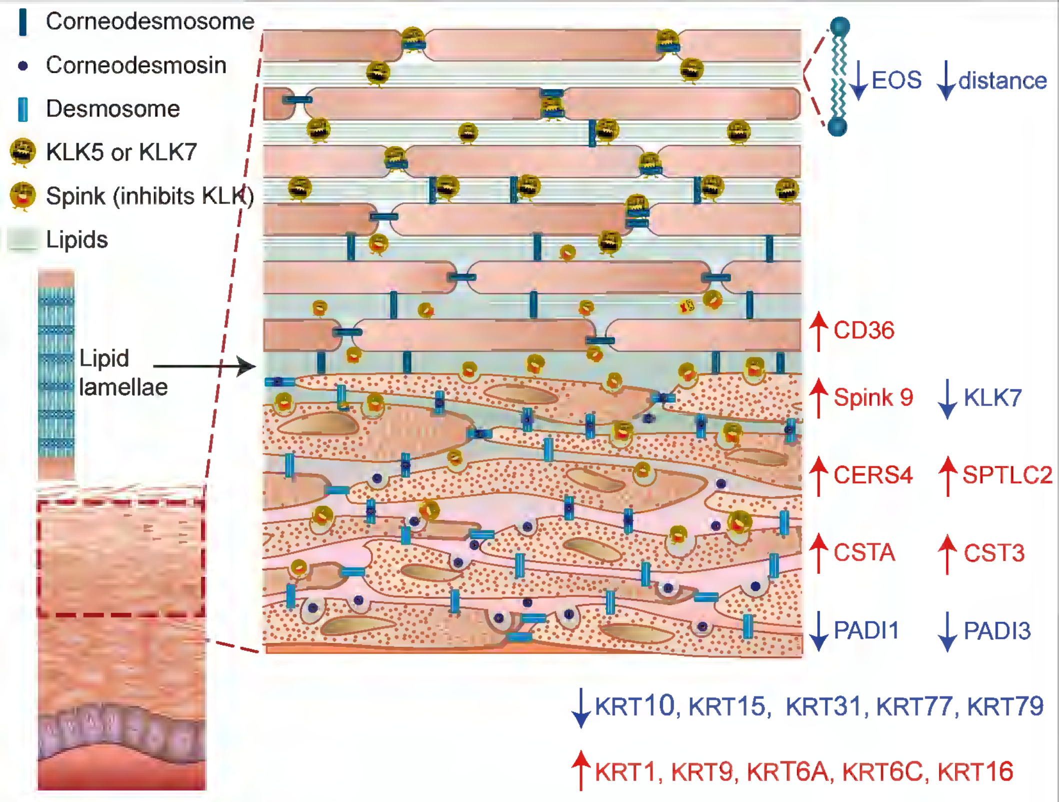

Corneodesmosome
Corneodesmosin
Desmosome
KLK5 or KLK7
Spink (inhibits KLK)
Lipids
Lipid lamellae
↓EOS ↓distance
↑CD36
↑Spink 9 ↓KLK7
↑CERS4 ↑SPTLC2
↑CSTA ↑CST3
↓PADI1 ↓PADI3
↓KRT10, KRT15, KRT31, KRT77, KRT79
↑KRT1, KRT9, KRT6A, KRT6C, KRT16

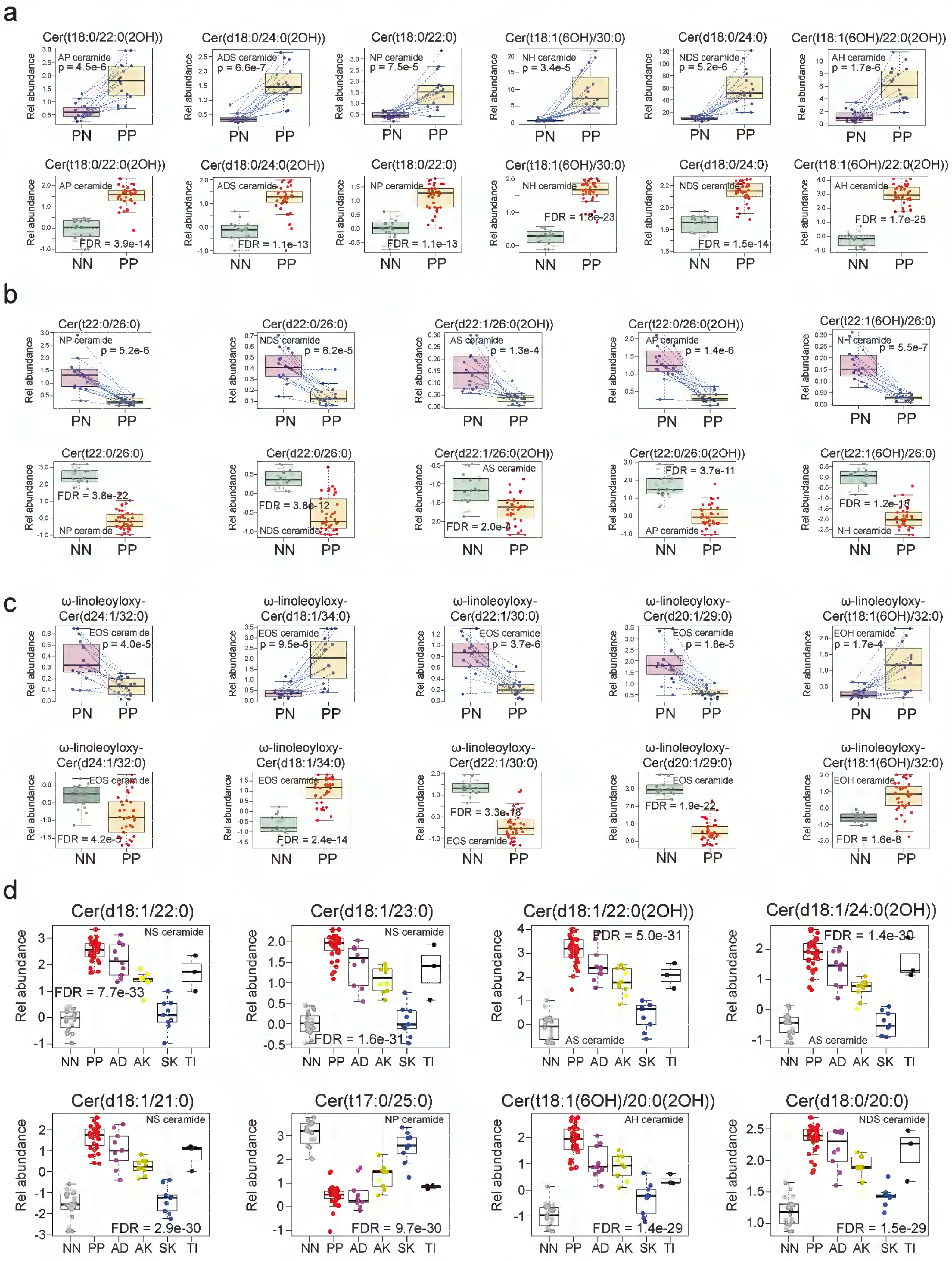
a
Cer(t18:0/22:0(2OH))
AP ceramide
p = 4.5e-6
Cer(d18:0/24:0(2OH))
ADS ceramide
p = 6.6e-7
Cer(t18:0/22:0)
NP ceramide
p = 7.5e-5
Cer(t18:1(6OH)/30:0)
NH ceramide
p = 3.4e-5
Cer(d18:0/24:0)
NDS ceramide
p = 5.2e-6
Cer(t18:1(6OH)/22:0(2OH))
AH ceramide
p = 1.7e-6
Rel abundance
PN
PP
Cer(t18:0/22:0(2OH))
AP ceramide
FDR = 3.9e-14
Cer(d18:0/24:0(2OH))
ADS ceramide
FDR = 1.1e-13
Cer(t18:0/22:0)
NP ceramide
FDR = 1.1e-13
Cer(t18:1(6OH)/30:0)
NH ceramide
FDR = 1.8e-23
Cer(d18:0/24:0)
NDS ceramide
FDR = 1.5e-14
Cer(t18:1(6OH)/22:0(2OH))
AH ceramide
FDR = 1.7e-25
NN
PP
b
Cer(t22:0/26:0)
NP ceramide
p = 5.2e-6
Cer(d22:0/26:0)
NDS ceramide
p = 8.2e-5
Cer(d22:1/26:0(2OH))
AS ceramide
p = 1.3e-4
Cer(t22:0/26:0(2OH))
AP ceramide
p = 1.4e-6
Cer(t22:1(6OH)/26:0)
NH ceramide
p = 5.5e-7
Cer(t22:0/26:0)
FDR = 3.8e-22
NP ceramide
Cer(d22:0/26:0)
FDR = 3.8e-12
NDS ceramide
Cer(d22:1/26:0(2OH))
AS ceramide
FDR = 2.0e-4
Cer(t22:0/26:0(2OH))
FDR = 3.7e-11
AP ceramide
Cer(t22:1(6OH)/26:0)
FDR = 1.2e-18
NH ceramide
c
ω-linoleoyloxy-Cer(d24:1/32:0)
EOS ceramide
p = 4.0e-5
ω-linoleoyloxy-Cer(d18:1/34:0)
EOS ceramide
p = 9.5e-6
ω-linoleoyloxy-Cer(d22:1/30:0)
EOS ceramide
p = 3.7e-6
ω-linoleoyloxy-Cer(d20:1/29:0)
EOS ceramide
p = 1.8e-5
ω-linoleoyloxy-Cer(t18:1(6OH)/32:0)
EOH ceramide
p = 1.7e-4
ω-linoleoyloxy-Cer(d24:1/32:0)
EOS ceramide
FDR = 4.2e-5
ω-linoleoyloxy-Cer(d18:1/34:0)
EOS ceramide
FDR = 2.4e-14
ω-linoleoyloxy-Cer(d22:1/30:0)
FDR = 3.3e-18
EOS ceramide
ω-linoleoyloxy-Cer(d20:1/29:0)
EOS ceramide
FDR = 1.9e-22
ω-linoleoyloxy-Cer(t18:1(6OH)/32:0)
EOH ceramide
FDR = 1.6e-8
d
Cer(d18:1/22:0)
NS ceramide
FDR = 7.7e-33
Cer(d18:1/23:0)
NS ceramide
FDR = 1.6e-31
Cer(d18:1/22:0(2OH))
FDR = 5.0e-31
AS ceramide
Cer(d18:1/24:0(2OH))
FDR = 1.4e-30
AS ceramide
NN PP AD AK SK TI
Cer(d18:1/21:0)
NS ceramide
FDR = 2.9e-30
Cer(t17:0/25:0)
NP ceramide
FDR = 9.7e-30
Cer(t18:1(6OH)/20:0(2OH))
AH ceramide
FDR = 1.4e-29
Cer(d18:0/20:0)
NDS ceramide
FDR = 1.5e-29

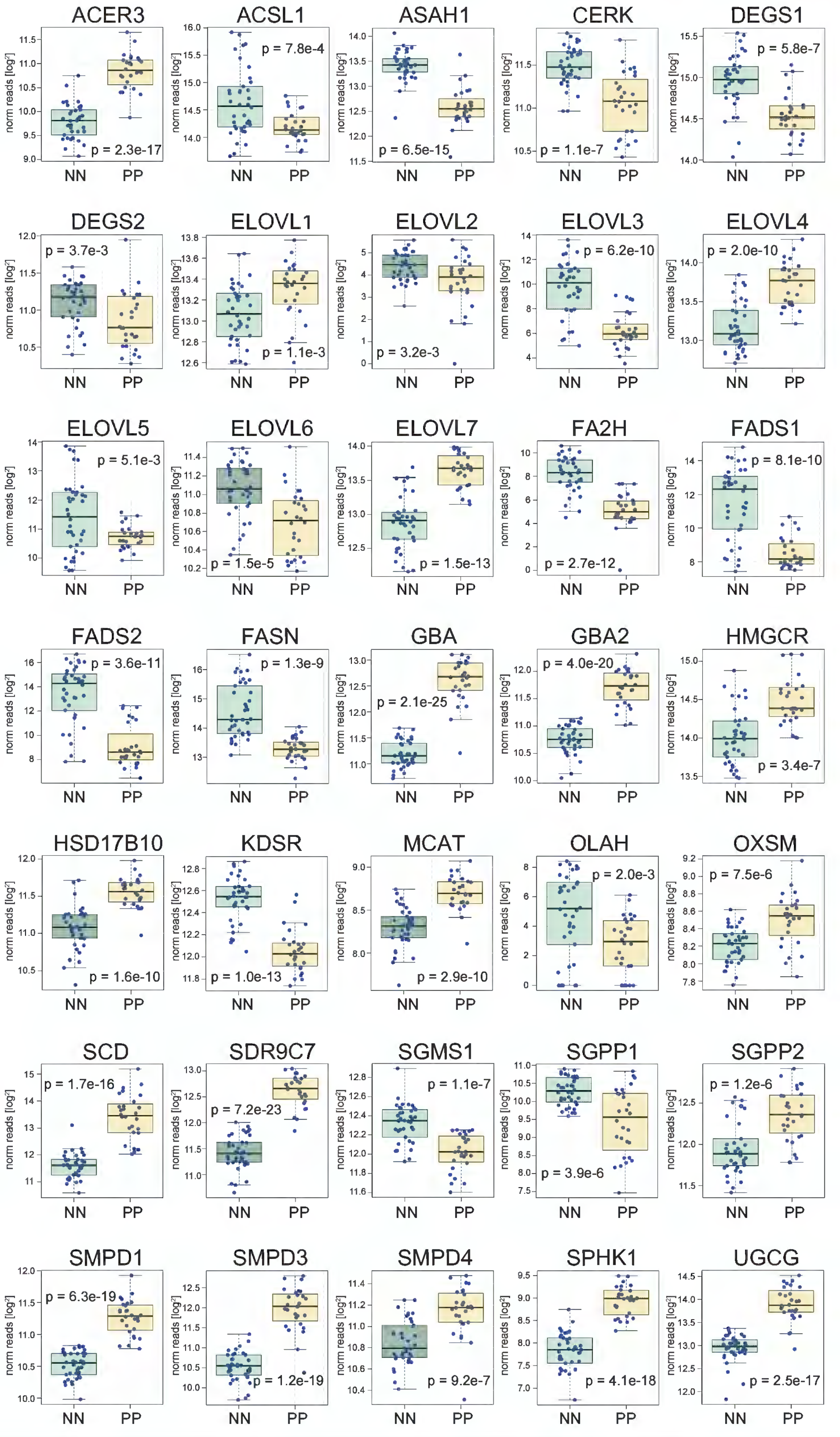
ACER3
p = 2.3e-17
ACSL1
p = 7.8e-4
ASAH1
p = 6.5e-15
CERK
p = 1.1e-7
DEGS1
p = 5.8e-7
DEGS2
p = 3.7e-3
ELOVL1
p = 1.1e-3
ELOVL2
p = 3.2e-3
ELOVL3
p = 6.2e-10
ELOVL4
p = 2.0e-10
ELOVL5
p = 5.1e-3
ELOVL6
p = 1.5e-5
ELOVL7
p = 1.5e-13
FA2H
p = 2.7e-12
FADS1
p = 8.1e-10
FADS2
p = 3.6e-11
FASN
p = 1.3e-9
GBA
p = 2.1e-25
GBA2
p = 4.0e-20
HMGCR
p = 3.4e-7
HSD17B10
p = 1.6e-10
KDSR
p = 1.0e-13
MCAT
p = 2.9e-10
OLAH
p = 2.0e-3
OXSM
p = 7.5e-6
SCD
p = 1.7e-16
SDR9C7
p = 7.2e-23
SGMS1
p = 1.1e-7
SGPP1
p = 3.9e-6
SGPP2
p = 1.2e-6
SMPD1
p = 6.3e-19
SMPD3
p = 1.2e-19
SMPD4
p = 9.2e-7
SPHK1
p = 4.1e-18
UGCG
p = 2.5e-17
norm reads [log2]
NN
PP

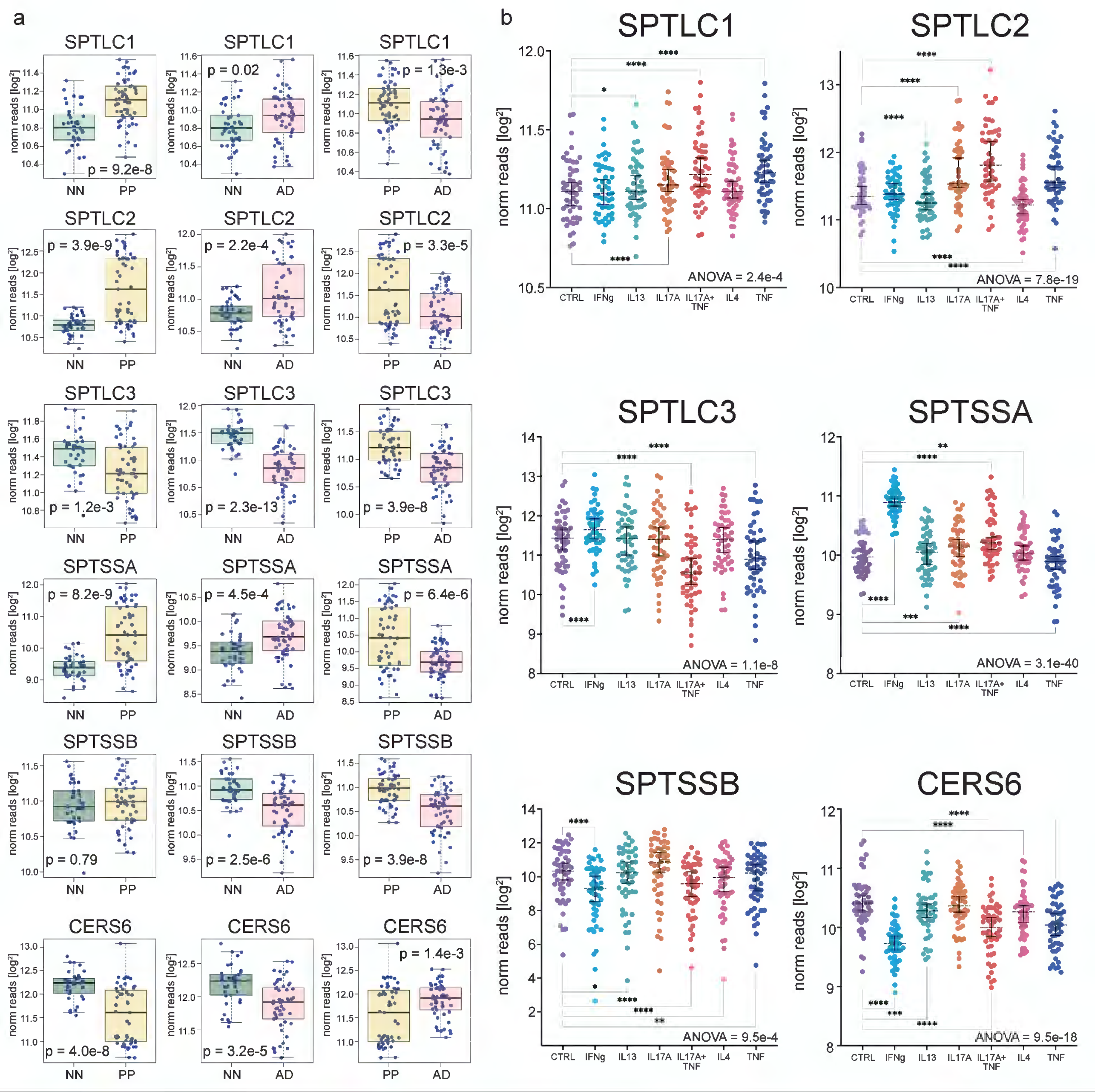
a
SPTLC1
p = 9.2e-8
p = 0.02
p = 1.3e-3
SPTLC2
p = 3.9e-9
p = 2.2e-4
p = 3.3e-5
SPTLC3
p = 1.2e-3
p = 2.3e-13
p = 3.9e-8
SPTSSA
p = 8.2e-9
p = 4.5e-4
p = 6.4e-6
SPTSSB
p = 0.79
p = 2.5e-6
p = 3.9e-8
CERS6
p = 4.0e-8
p = 3.2e-5
p = 1.4e-3
NN
PP
AD
norm reads [log²]
b
SPTLC1
ANOVA = 2.4e-4
SPTLC2
ANOVA = 7.8e-19
SPTLC3
ANOVA = 1.1e-8
SPTSSA
ANOVA = 3.1e-40
SPTSSB
ANOVA = 9.5e-4
CERS6
ANOVA = 9.5e-18
CTRL
IFNg
IL13
IL17A
IL17A+ TNF
IL4
TNF
norm reads [log²]

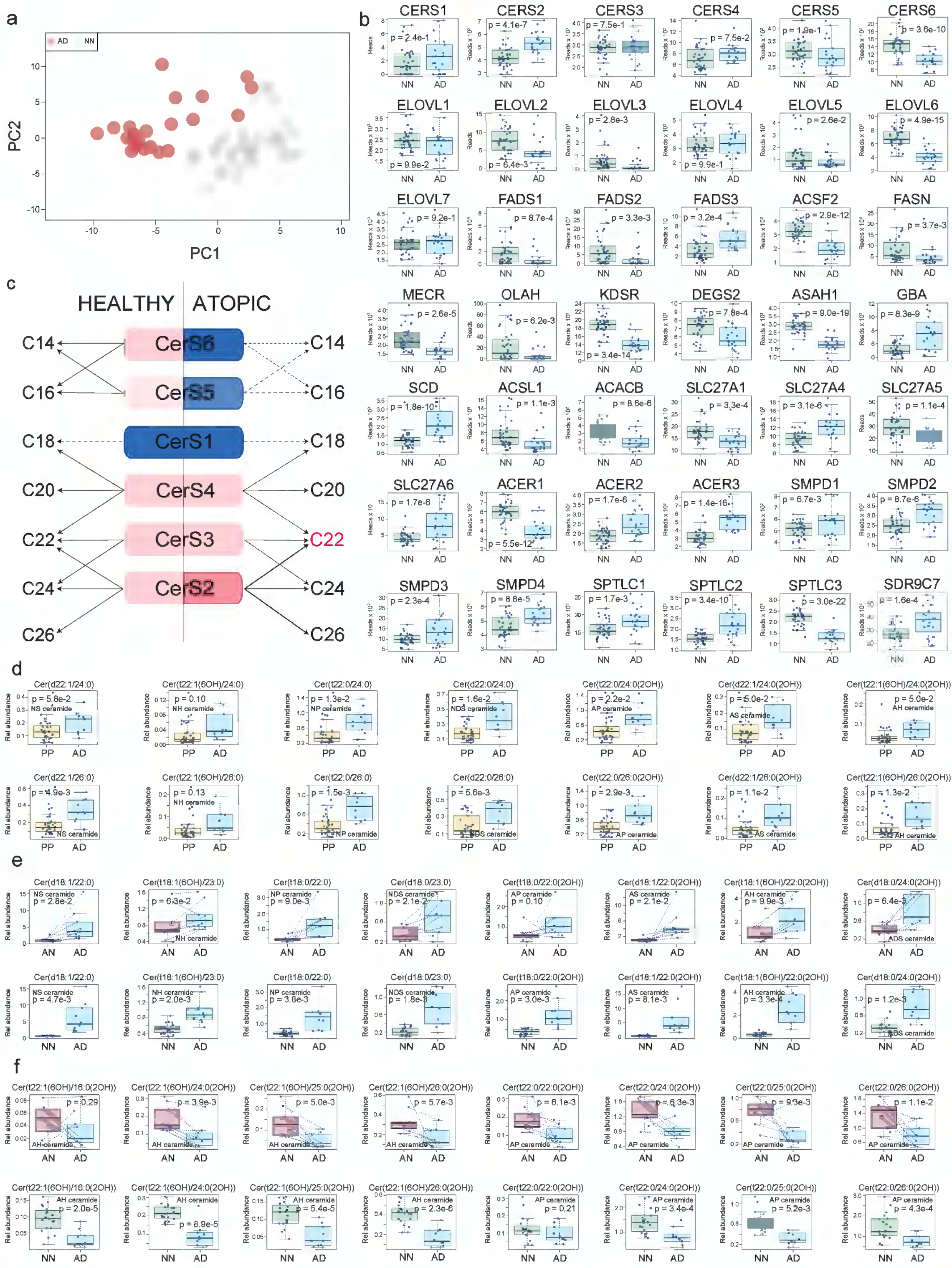

a
AD
NN
PC1
PC2
b
CERS1 p = 2.4e-1
CERS2 p = 4.1e-7
CERS3 p = 7.5e-1
CERS4 p = 7.5e-2
CERS5 p = 1.9e-1
CERS6 p = 3.6e-10
ELOVL1 p = 9.9e-2
ELOVL2 p = 6.4e-3
ELOVL3 p = 2.8e-3
ELOVL4 p = 9.9e-1
ELOVL5 p = 2.6e-2
ELOVL6 p = 4.9e-15
ELOVL7 p = 9.2e-1
FADS1 p = 8.7e-4
FADS2 p = 3.3e-3
FADS3 p = 3.2e-4
ACSF2 p = 2.9e-12
FASN p = 3.7e-3
MECR p = 2.6e-5
OLAH p = 6.2e-3
KDSR p = 3.4e-14
DEGS2 p = 7.8e-4
ASAH1 p = 9.0e-19
GBA p = 8.3e-9
SCD p = 1.8e-10
ACSL1 p = 1.1e-3
ACACB p = 8.6e-6
SLC27A1 p = 3.3e-4
SLC27A4 p = 3.1e-6
SLC27A5 p = 1.1e-4
SLC27A6 p = 1.7e-6
ACER1 p = 5.5e-12
ACER2 p = 1.7e-6
ACER3 p = 1.4e-16
SMPD1 p = 6.7e-3
SMPD2 p = 8.7e-6
SMPD3 p = 2.3e-4
SMPD4 p = 8.8e-5
SPTLC1 p = 1.7e-3
SPTLC2 p = 3.4e-10
SPTLC3 p = 3.0e-22
SDR9C7 p = 1.6e-4
NN AD
Reads
c
HEALTHY
ATOPIC
CerS6
CerS5
CerS1
CerS4
CerS3
CerS2
C14
C16
C18
C20
C22
C24
C26
d
Cer(d22:1/24:0) p = 5.8e-2 NS ceramide
Cer(t22:1(6OH)/24:0) p = 0.10 NH ceramide
Cer(t22:0/24:0) p = 1.3e-2 NP ceramide
Cer(d22:0/24:0) p = 1.6e-2 NDS ceramide
Cer(t22:0/24:0(2OH)) p = 2.2e-2 AP ceramide
Cer(d22:1/24:0(2OH)) p = 5.0e-2 AS ceramide
Cer(t22:1(6OH)/24:0(2OH)) p = 5.0e-2 AH ceramide
Cer(d22:1/26:0) p = 4.9e-3 NS ceramide
Cer(t22:1(6OH)/26:0) p = 0.13 NH ceramide
Cer(t22:0/26:0) p = 1.5e-3 NP ceramide
Cer(d22:0/26:0) p = 5.6e-3 NDS ceramide
Cer(t22:0/26:0(2OH)) p = 2.9e-3 AP ceramide
Cer(d22:1/26:0(2OH)) p = 1.1e-2 AS ceramide
Cer(t22:1(6OH)/26:0(2OH)) p = 1.3e-2 AH ceramide
PP AD
Rel abundance
e
Cer(d18:1/22:0) NS ceramide p = 2.8e-2
Cer(t18:1(6OH)/23:0) p = 6.3e-2 NH ceramide
Cer(t18:0/22:0) NP ceramide p = 9.0e-3
Cer(d18:0/23:0) NDS ceramide p = 2.1e-2
Cer(t18:0/22:0(2OH)) AP ceramide p = 0.10
Cer(d18:1/22:0(2OH)) AS ceramide p = 2.1e-2
Cer(t18:1(6OH)/22:0(2OH)) AH ceramide p = 9.9e-3
Cer(d18:0/24:0(2OH)) p = 6.4e-3 ADS ceramide
AN AD
Cer(d18:1/22:0) NS ceramide p = 4.7e-3
Cer(t18:1(6OH)/23:0) NH ceramide p = 2.0e-3
Cer(t18:0/22:0) NP ceramide p = 3.8e-3
Cer(d18:0/23:0) NDS ceramide p = 1.8e-3
Cer(t18:0/22:0(2OH)) AP ceramide p = 3.0e-3
Cer(d18:1/22:0(2OH)) AS ceramide p = 8.1e-3
Cer(t18:1(6OH)/22:0(2OH)) AH ceramide p = 3.3e-4
Cer(d18:0/24:0(2OH)) p = 1.2e-3 ADS ceramide
NN AD
f
Cer(t22:1(6OH)/16:0(2OH)) p = 0.29 AH ceramide
Cer(t22:1(6OH)/24:0(2OH)) p = 3.9e-3 AH ceramide
Cer(t22:1(6OH)/25:0(2OH)) p = 5.0e-3 AH ceramide
Cer(t22:1(6OH)/26:0(2OH)) p = 5.7e-3 AH ceramide
Cer(t22:0/22:0(2OH)) p = 6.1e-3 AP ceramide
Cer(t22:0/24:0(2OH)) p = 6.3e-3 AP ceramide
Cer(t22:0/25:0(2OH)) p = 9.3e-3 AP ceramide
Cer(t22:0/26:0(2OH)) p = 1.1e-2 AP ceramide
AN AD
Cer(t22:1(6OH)/16:0(2OH)) AH ceramide p = 2.0e-5
Cer(t22:1(6OH)/24:0(2OH)) AH ceramide p = 8.9e-5
Cer(t22:1(6OH)/25:0(2OH)) AH ceramide p = 5.4e-5
Cer(t22:1(6OH)/26:0(2OH)) AH ceramide p = 2.3e-6
Cer(t22:0/22:0(2OH)) AP ceramide p = 0.21
Cer(t22:0/24:0(2OH)) AP ceramide p = 3.4e-4
Cer(t22:0/25:0(2OH)) AP ceramide p = 5.2e-3
Cer(t22:0/26:0(2OH)) AP ceramide p = 4.3e-4
NN AD

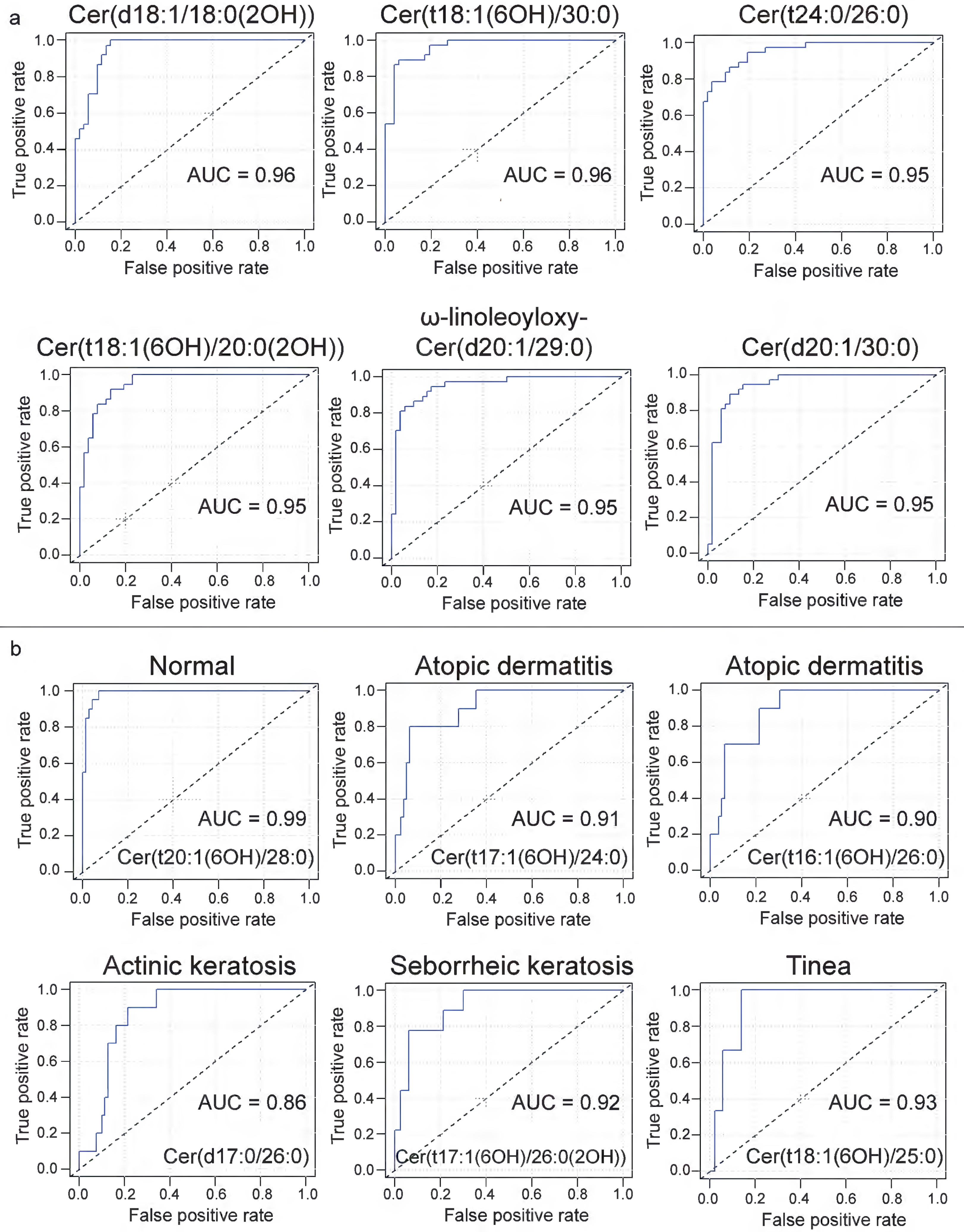
a
Cer(d18:1/18:0(2OH))
AUC = 0.96
Cer(t18:1(6OH)/30:0)
AUC = 0.96
Cer(t24:0/26:0)
AUC = 0.95
Cer(t18:1(6OH)/20:0(2OH))
AUC = 0.95
ω-linoleoyloxy-Cer(d20:1/29:0)
AUC = 0.95
Cer(d20:1/30:0)
AUC = 0.95
b
Normal
AUC = 0.99
Cer(t20:1(6OH)/28:0)
Atopic dermatitis
AUC = 0.91
Cer(t17:1(6OH)/24:0)
Atopic dermatitis
AUC = 0.90
Cer(t16:1(6OH)/26:0)
Actinic keratosis
AUC = 0.86
Cer(d17:0/26:0)
Seborrheic keratosis
AUC = 0.92
Cer(t17:1(6OH)/26:0(2OH))
Tinea
AUC = 0.93
Cer(t18:1(6OH)/25:0)
True positive rate
False positive rate

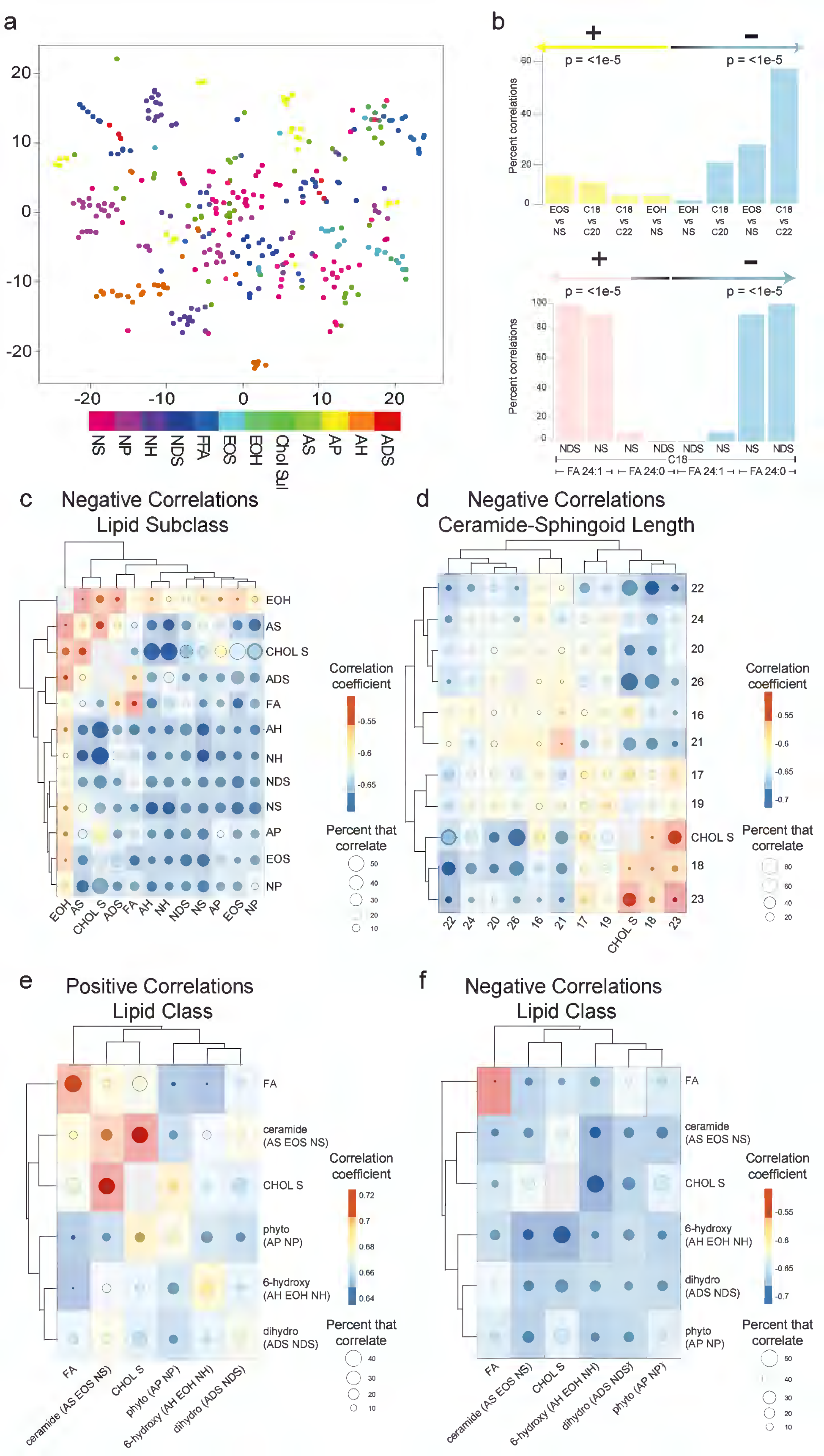

a
b
p = <1e-5
p = <1e-5
Percent correlations
EOS vs NS
C18 vs C20
C18 vs C22
EOH vs NS
EOH vs NS
C18 vs C20
EOS vs NS
C18 vs C22
NS
NP
NH
NDS
FFA
EOS
EOH
Chol Sul
AS
AP
AH
ADS
NDS
NS
NS
NDS
NDS
NS
NS
NDS
C18
FA 24:1
FA 24:0
FA 24:1
FA 24:0
c
Negative Correlations
Lipid Subclass
EOH
AS
CHOL S
ADS
FA
AH
NH
NDS
NS
AP
EOS
NP
Correlation coefficient
Percent that correlate
d
Negative Correlations
Ceramide-Sphingoid Length
CHOL S
Correlation coefficient
Percent that correlate
e
Positive Correlations
Lipid Class
FA
ceramide (AS EOS NS)
CHOL S
phyto (AP NP)
6-hydroxy (AH EOH NH)
dihydro (ADS NDS)
Correlation coefficient
Percent that correlate
f
Negative Correlations
Lipid Class
FA
ceramide (AS EOS NS)
CHOL S
6-hydroxy (AH EOH NH)
dihydro (ADS NDS)
phyto (AP NP)
Correlation coefficient
Percent that correlate

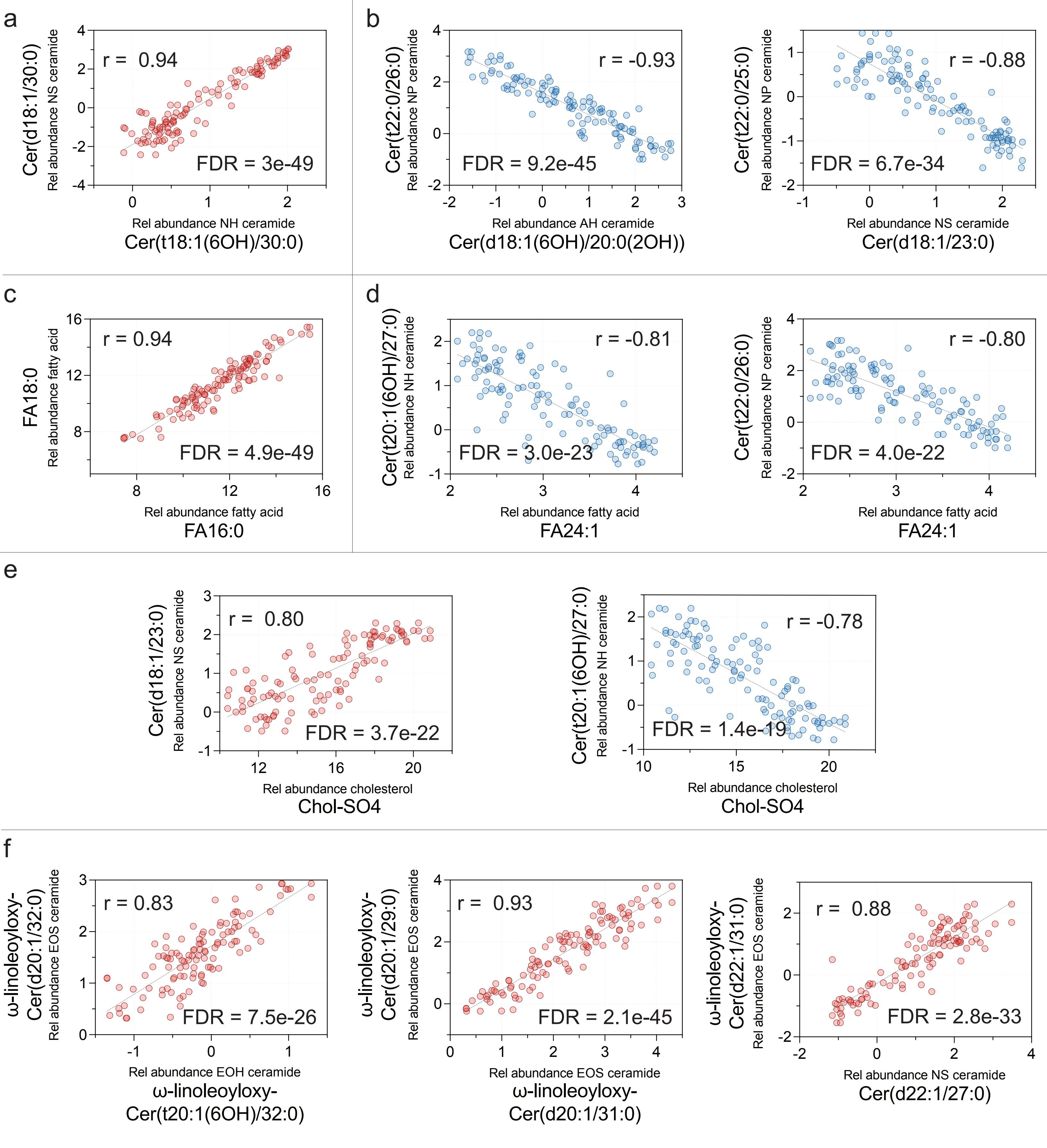
a
r = 0.94
FDR = 3e-49
Cer(d18:1/30:0)
Rel abundance NS ceramide
Rel abundance NH ceramide
Cer(t18:1(6OH)/30:0)
b
r = -0.93
FDR = 9.2e-45
Cer(t22:0/26:0)
Rel abundance NP ceramide
Rel abundance AH ceramide
Cer(d18:1(6OH)/20:0(2OH))
r = -0.88
FDR = 6.7e-34
Cer(t22:0/25:0)
Rel abundance NP ceramide
Rel abundance NS ceramide
Cer(d18:1/23:0)
c
r = 0.94
FDR = 4.9e-49
FA18:0
Rel abundance fatty acid
Rel abundance fatty acid
FA16:0
d
r = -0.81
FDR = 3.0e-23
Cer(t20:1(6OH)/27:0)
Rel abundance NH ceramide
Rel abundance fatty acid
FA24:1
r = -0.80
FDR = 4.0e-22
Cer(t22:0/26:0)
Rel abundance NP ceramide
Rel abundance fatty acid
FA24:1
e
r = 0.80
FDR = 3.7e-22
Cer(d18:1/23:0)
Rel abundance NS ceramide
Rel abundance cholesterol
Chol-SO4
r = -0.78
FDR = 1.4e-19
Cer(t20:1(6OH)/27:0)
Rel abundance NH ceramide
Rel abundance cholesterol
Chol-SO4
f
r = 0.83
FDR = 7.5e-26
ω-linoleoyloxy-Cer(d20:1/32:0)
Rel abundance EOS ceramide
Rel abundance EOH ceramide
ω-linoleoyloxy-Cer(t20:1(6OH)/32:0)
r = 0.93
FDR = 2.1e-45
ω-linoleoyloxy-Cer(d20:1/29:0)
Rel abundance EOS ceramide
Rel abundance EOS ceramide
ω-linoleoyloxy-Cer(d20:1/31:0)
r = 0.88
FDR = 2.8e-33
ω-linoleoyloxy-Cer(d22:1/31:0)
Rel abundance EOS ceramide
Rel abundance NS ceramide
Cer(d22:1/27:0)

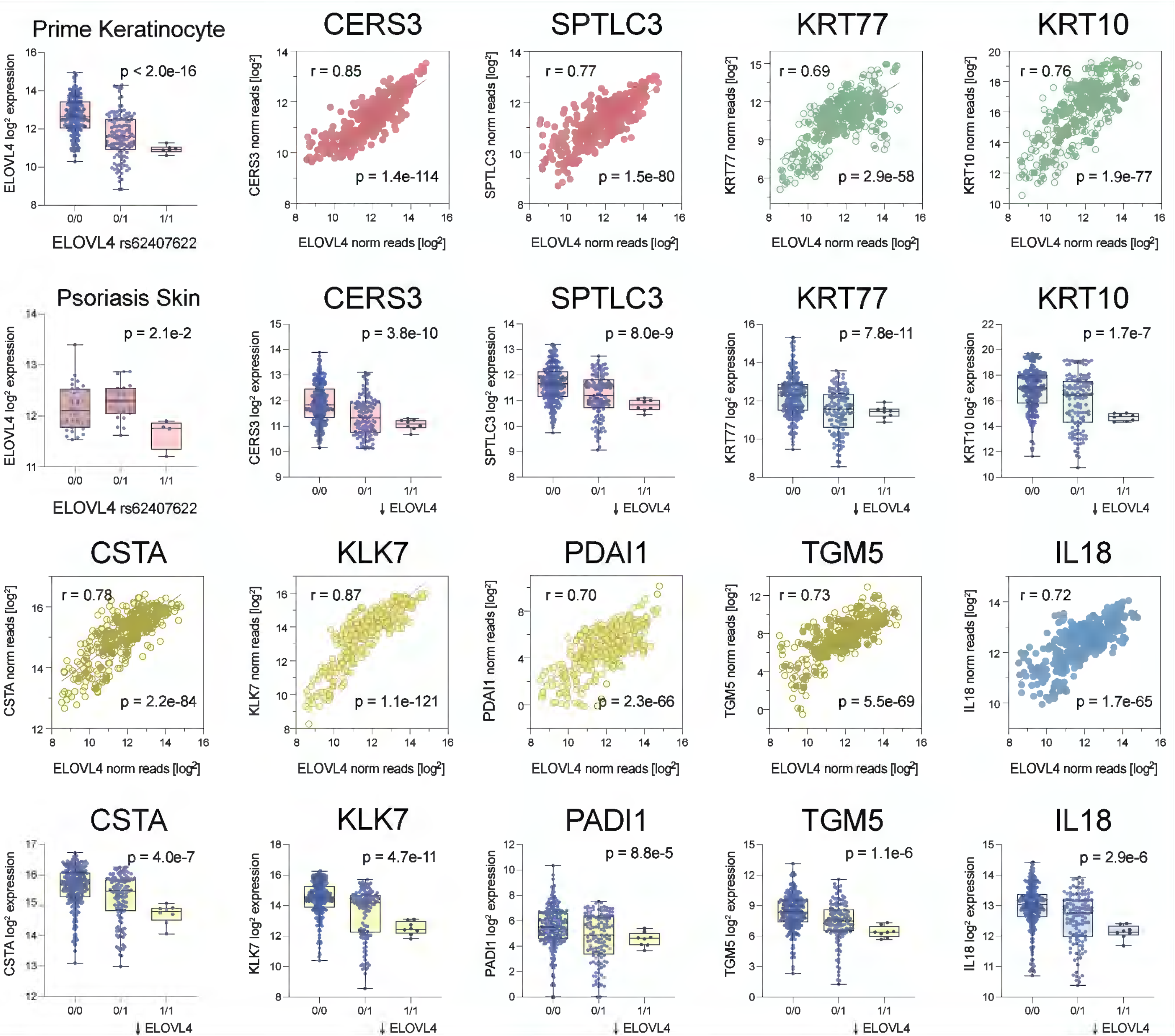

Prime Keratinocyte
ELOVL4 log² expression
p < 2.0e-16
0/0
0/1
1/1
ELOVL4 rs62407622
CERS3
CERS3 norm reads [log²]
r = 0.85
p = 1.4e-114
ELOVL4 norm reads [log²]
SPTLC3
SPTLC3 norm reads [log²]
r = 0.77
p = 1.5e-80
KRT77
KRT77 norm reads [log²]
r = 0.69
p = 2.9e-58
KRT10
KRT10 norm reads [log²]
r = 0.76
p = 1.9e-77
Psoriasis Skin
p = 2.1e-2
CERS3 log² expression
p = 3.8e-10
↓ ELOVL4
SPTLC3 log² expression
p = 8.0e-9
KRT77 log² expression
p = 7.8e-11
KRT10 log² expression
p = 1.7e-7
CSTA
CSTA norm reads [log²]
r = 0.78
p = 2.2e-84
KLK7
KLK7 norm reads [log²]
r = 0.87
p = 1.1e-121
PDAI1
PDAI1 norm reads [log²]
r = 0.70
p = 2.3e-66
TGM5
TGM5 norm reads [log²]
r = 0.73
p = 5.5e-69
IL18
IL18 norm reads [log²]
r = 0.72
p = 1.7e-65
CSTA log² expression
p = 4.0e-7
KLK7 log² expression
p = 4.7e-11
PADI1
PADI1 log² expression
p = 8.8e-5
TGM5 log² expression
p = 1.1e-6
IL18 log² expression
p = 2.9e-6
