# Supplementary material for: Biogeographic and disease-specific alterations in epidermal lipid composition and single-cell analysis of acral keratinocytes
Source: JCI Insight. 2022 Aug 22;7(16):e159762. doi: 10.1172/jci.insight.159762 (PMC9462509; doi:10.1172/jci.insight.159762)
Supplement: Supplemental data set 1 [file jciinsight-7-159762-s280.pdf]

# SOP TITLE: *D-Squame Disc Extraction for Stratum Corneum Ceramide Panel*

## 1 PURPOSE

- 1.1 Provides detailed instructions on how to prepare lipids from D-Squame disc matrix to run on the Stratum Corneum Lipid Panel (SFC/5500 QTRAP).

## 2 DEFINITIONS

- 2.1 SFC - Supercritical Fluid Chromatography (Waters UPC2)
- 2.2 5500 QTRAP - SCIEX 5500 QTRAP Mass Spec

## 3 MATERIALS AND EQUIPMENT

- 3.1 ***Materials:***
  - Methanol, Optima (Fisher)
  - Hexane, Optima (Fisher)
  - Chloroform, Optima (Fisher)
  - Ethanol
  - Water, Low TOC, HPLC grade
  - Potassium chloride
  - 8ml clear glass vials with PTFE lined caps (Fisher)
  - 16ml glass extraction tubes (Fisher)
  - 2ml glass vials (Fisher)
  - 0.2µm PTFE centrifuge filters (Millipore)
  - Glass inserts, 250ul (Agilent)
  - 5" or 9" glass Pasteur pipettes
  - Rubber bulb
  - Blue caps (Agilent)
  - D-Squame Discs, standard size (CuDerm)
  - Ammonium acetate, LC/MS grade
  - OPTIONAL: Repeater pipette tips
- 3.2 ***Equipment:***
  - Metal tweezers
  - 200ul and 1ml pipettes with filtered tips (Rainin)
  - Vortex
  - Sonicator
  - Centrifuge
  - Nitrogen manifold
  - TurboVap (45 °C)
  - OPTIONAL: Bottle top dispensettes
  - OPTIONAL: Repeater pipette

## 4 PROCEDURE

- 4.1 **PROCEDURE PART I: Extraction preparation**
  - The PPE required for this SOP includes a lab coat, nitrile gloves and eye protection. Work in a properly ventilated environment.
  - Solution preparation:
    - NOTE: The volumes can be scaled by adjusting the constituents proportionately.
    - Chloroform:Methanol (2:1, v/v)
      - 600mL Chloroform
      - 300mL Methanol
    - 10mM Ammonium acetate in Chloroform:Methanol (3:1, v/v)
      - 0.309g Ammonium acetate, acceptable range (+/- 1%): 0.306 - 0.312g
      - 300mL Chloroform
      - 100mL Methanol
  - Set up:
    - Prepare a jar of EtOH
      - Metal spatulas and tweezers that come into contact with raw sample must be cleaned in EtOH and wiped with a kimwipe.
    - Thaw the discs for 30 minutes at ambient temperature.
    - Thaw the IN and QC for 30 minutes at 37°C. Do not exceed the 30 minutes for more than 15 minutes.
    - Label the following:
      - 1 set of 8ml glass vials with caps (place the labels along the top edge)
      - 1 set of extraction tubes
      - 2 sets of 2ml vials
      - 1 set of 0.2µm centrifuge filters

- 4.2 **PROCEDURE PART II: Disc Extraction**
  - Thaw IN for 30 minutes at 37 degrees.
  - TISSUE ONLY: Weigh samples
    - Weigh each sample in an 8ml vial. Record the weight in Batch Sheet.
  - Volumetrically add 20ul Internal Standard to each vial. Add 4ml Methanol (5ml for large discs). Cap vial.
  - CONTROLS ONLY: Mix the QC mix by plunging a pipette a few times in the insert. Discard the tip. Volumetrically add 20ul QC mix to QC### samples.
  - Transfer one disc to the vial, sticky side facing in for standard and large discs, swirl the vial to wet the disc. Repeat with remaining discs.
    - BLANKS: Use the same number of new standard sized D-Squame discs as client samples
    - CONTROLS: Use two new standard sized D-Squame discs
    - Clean tweezers in EtOH and dry with a kimwipe between samples.
  - TISSUE ONLY: Homogenize the tissue sample.
    - Clean a glass homogenizer (soapy water/brush, water, DIUF water, Methanol, then Chloroform)
    - Carefully pour the sample in methanol into the homogenizer. Homogenize sample.
    - Use a glass pipette to transfer the contents from the homogenizer back to the vial.
    - Rinse homogenizer twice with Methanol between samples.
  - Sonicate the vials for 15 minutes.
  - Centrifuge vials for 20 minutes at speed 6.
    - Place 2-2ml vials with caps in the rotor as spacers for the vials.
  - Transfer samples to the extraction tube using glass pipettes.
    - Save the caps.
  - Dispense 4ml of hexane (5ml for large discs) to the 8ml vials and recap using the same caps.
  - Sonicate for 15 minutes.
  - Add 2ml KCl to each vial. Recap using the same caps.
  - Centrifuge the vials for 20 minutes at speed 8.
    - Place 2-2ml vials with caps in the rotor as spacers for the vials.
  - Dry the extraction tubes (with MeOH) at 45°C, 15-20psi (takes about 20 minutes).
  - Transfer top liquid portion of the samples to same set of extraction tubes as the now dry MeOH using glass pipettes.
    - Take as much of the top layer as possible, without disrupting the bi-layer
  - Dry the tubes at 45°C, 15-20psi (takes about 10 minutes).
  - Reconstitute samples in 400ul Chloroform:Methanol (2:1). Vortex briefly.
  - Transfer samples to corresponding 0.2µm centrifuge filter tubes.
  - Centrifuge at 17,000g for 10 minutes at ambient temperature.
    - If there is still unfiltered sample left, re-spin for an additional 5 minutes.
    - If sample still will not filter completely, transfer to a new centrifuge filter and spin for 5 minutes. Pool samples.
  - Transfer samples evenly between the two corresponding 2ml vials.
    - Careful not to disrupt the pellet at the bottom of the vial
    - Cap under nitrogen and store 1 set at -80°C
  - Dry samples under a stream of nitrogen until completely dry.
    - Sample will be dry when there is no liquid movement in the vial. There will likely be some adhesive residue, but it shouldn't move when shaken.
  - Reconstitute samples in 100ul 10mM Ammonium acetate in Chloroform:Methanol (3:1). Vortex and transfer to inserts. Cap with blue caps.

# SOP TITLE: *Daily QC for Stratum Corneum Lipids on SFC-5500*

## 1 PURPOSE

- 1.1 Provides detailed instructions on how to run the daily QC check for Stratum Corneum Lipid samples on the SFC/5500 QTRAP.

## 2 DEFINITIONS

- 2.1SFC - Supercritical Fluid Chromatography (Waters UPC2)
  - SM: Sample Manager (Weak, Strong Needle Wash)
  - BSM: Binary Solvent Manager/Chromatographic Pump (Solvent lines B1-B4, CO2, Seal Wash)
  - ISM: Isocratic Solvent Manager/Regeneration Pump (ISM, Seal Wash)
  - CM: Column Manager (Columns 1-4)
  - CCM: Convergence Chromatography Manager/Convergence Manager (CO2)

- 2.2 5500 QTRAP - SCIEX 5500 QTRAP Mass Spec

## 3 MATERIALS AND EQUIPMENT

- 3.1 ***Assay Components:***
  - SCRT: Retention time standard

- 3.2 ***Materials:***
  - Methanol, LCMS grade
  - Water, LCMS grade
  - Formic acid, LCMS grade
  - Ammoium formate, LCMS grade
  - Ammonium acetate, LCMS grade
  - Isopropanol, LCMS grade
  - Chloroform, HPLC grade
  - (Optional) Tetrahydrofuran (THF), HPLC grade

## 4 PROCEDURE

- 4.1 **PROCEDURE PART I: Prepare Solutions**
  - The PPE required for this SOP includes a lab coat, nitrile gloves and eye protection. Work in a properly ventilated environment.
  - Solution preparation:
    - NOTE: The volumes can be scaled by adjusting the constituents proportionately.
    - Methanol:Water (95:5) + 0.1% Formic acid
      - 1900mL Methanol
      - 100mL Water
      - 2ml Formic acid
    - 10mM Ammonium formate in Methanol:IPA (85:15) + 0.1% Formic acid
      - 1.2612g Ammonium formate, acceptable range (+/- 1%): 1.2599 - 1.2625g
      - 1700mL Methanol
      - 300mL 2-Propanol
      - 2mL Formic acid
    - Methanol:IPA (95:5)
      - 475mL Methanol
      - 25mL Isopropanol
    - 10mM Ammonium acetate in Chloroform:Methanol (3:1)
      - 0.3083g Ammonium acetate, acceptable range (+/- 1%): 0.3052 - 0.3113g
      - 300mL Chloroform
      - 100mL Methanol

- 4.2 **PROCEDURE PART II: Prepare RT Standard Sample, Blank, and SFC solutions**
  - Blank: Fill a 2mL vial with approximately 1mL 10mM Ammonium acetate in Chloroform:Methanol (3:1)
  - Stratum Corneum (SC) RT: Thaw a vial of RT Standard Sample on the bench top for 10-15 minutes
  - Place the Blank in Plate 1, vial position A,1
  - Place the RT SC in Plate 1, vial position F,8
  - The following minimum solution volumes are sufficient for 70 injections, QC checks, and purge
    - (B1) **10mM Ammonium formate in Methanol:IPA (85:15) + 0.1% Formic acid**: 400ml
    - (B2) NA
    - (B3) NA
    - (B4) NA
    - (ISM) **Methanol:Water(95:5) + 0.1% Formic acid**: 500ml
    - (Weak Needle Wash) **Methanol**: 300ml
    - (Strong Needle Wash) **Methanol:IPA (95:5)**: 150ml
    - (Seal Wash) **Methanol**: 300ml
- 4.3 **PROCEDURE PART III: Wipe Down Instrument**
  - Open the door to the Sample Manager, wipe down the foot of the sample needle using a kimwipe dipped in Chloroform, IPA, or an appropriate solvent mix.
    - Take caution when wiping the foot as the puncture needle is sharp.
  - Clean electrode
    - Deactivate the hardware profile in Analyst by double-clicking "Hardware Configuration". Deactivate "SFC-QTRAP".
    - Remove the source from the 5500, use a kimwipe dipped in methanol to gently wipe any residue off the tip of the electrode.
      - Take caution not to puncture your glove when wiping the electrode.
    - Activate the "SFC-QTRAP" hardware profile and close the window.

- 4.4 **PROCEDURE PART IV: Equilibrate SFC**
  - On the Waters computer, open MassLynx and the Acquity Console by clicking on the icons on the desktop or go to Start Menu > MassLynx V4.1 (and) ACQUITY Console
  - In MassLynx, open the Inlet Method window by clicking on "Inlet Method" on the left side of the software.
  - In the ACQUITY Console, go to ACQUITY UPLC System, select the Control drop down menu, then Start Up System.

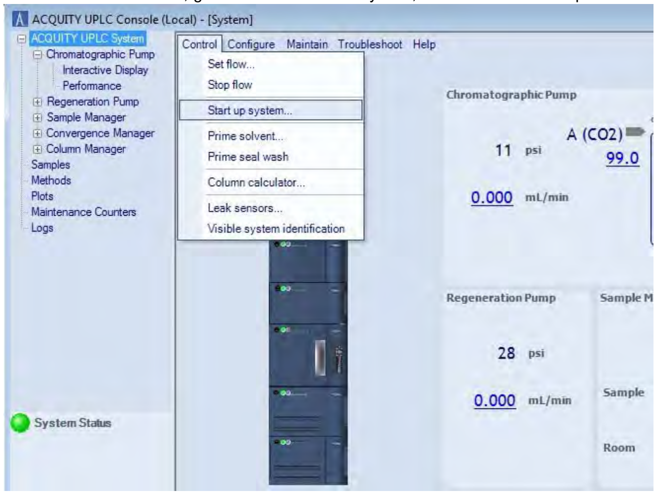


  - Each window should be set accordingly:

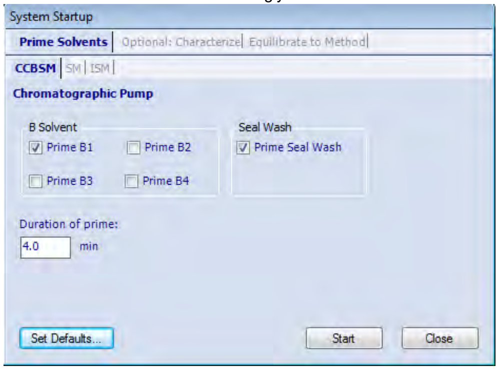


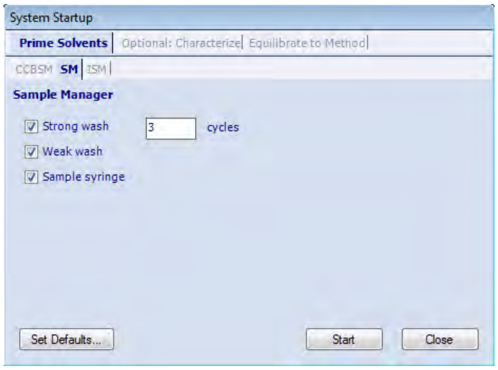


If either the sample needle or sample loop or both have been replaced, click the Optional:Characterize Volume tab, and then select "Characterize seal" and "Characterize needle and loop volumes". Otherwise, leave unchecked.

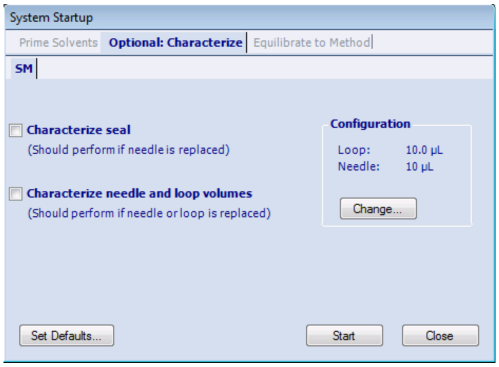

- Select the appropriate column for the day in the CM tab.

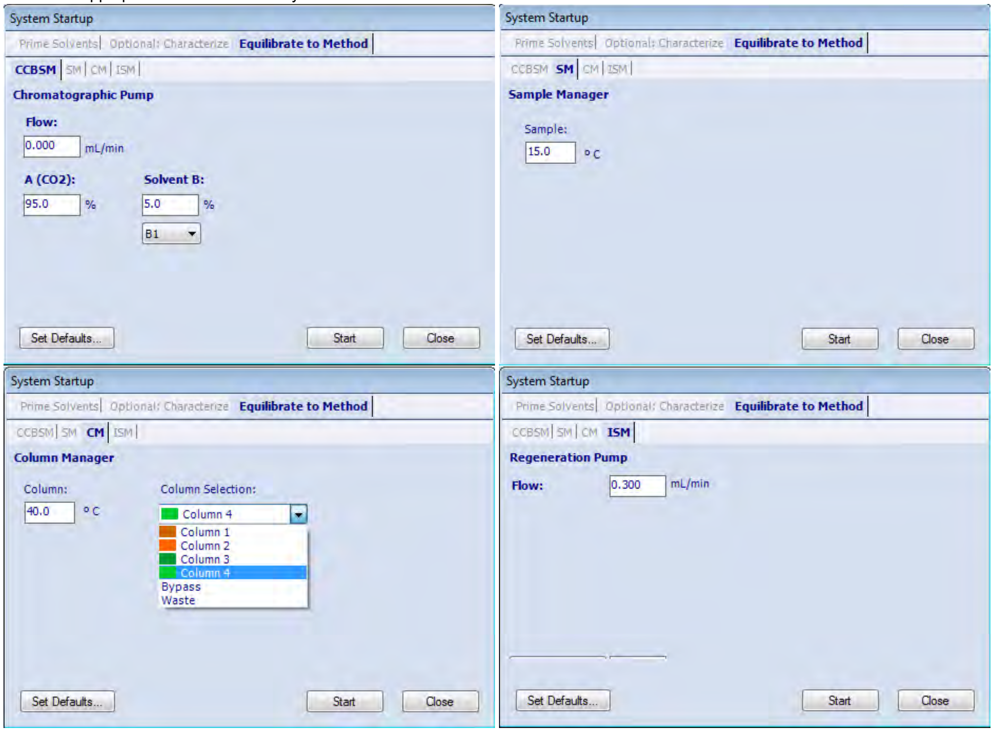


- Click Start.
- The purge will take 8 minutes and then turn on the Regeneration Pump (ISM) to 0.3ml/min. In the mean time, switch the screen to SC5500 to start equilibration on Analyst.

- 4.5 **PROCEDURE PART V: Switch screen to Analyst to operate**
  - On the right side of the monitor, click on the third button to get to **Menu**, then to **Input Source**. Use the Up and Down buttons to access between **VGA** and **DisplayPort** .

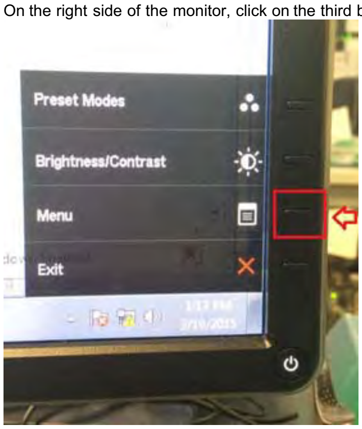

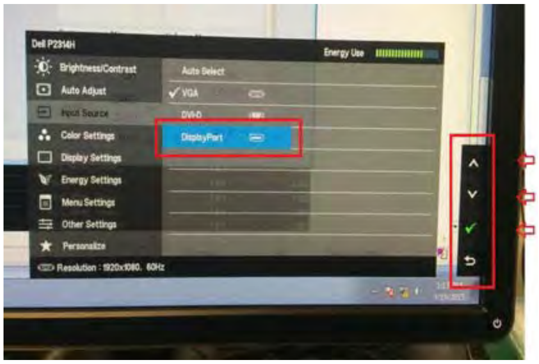


  - Select **DisplayPort** on the screen, then click on the green check mark button. Now the screen would switch to the SCIEX computer, where Analyst can be accessed.
- 4.6 **PROCEDURE PART VI: Set up SCRT standard in Analyst**
  - On the SCIEX computer, open Analyst 1.6.2 by clicking on the icon on the desktop or go to Start Menu > Analyst
  - In the **Configure** navigation, double-click on "Hardware Configuration". Ensure that "SFC-QTRAP" is active (GREEN CHECK). Close the window.
    - If not active, deactivate any other active profiles (YELLOW AND BLUE CHECK), then highlight "SFC-QTRAP" and click the "Activate profile" button. (GREEN CHECK). Close the window.
  - Ensure the QC project folder is assigned in the Root Directory.
  - At the top of the toolbar, choose the correct Project Folder "SCLipids_Date_Month_Year"
  - Click on the Configure tab in the left column.
  - Click on the "Tools" menu in the toolbar
  - Click on "Settings" and "Queue Options"
  - Change the "Max Idle Time" to "999". Click OK.

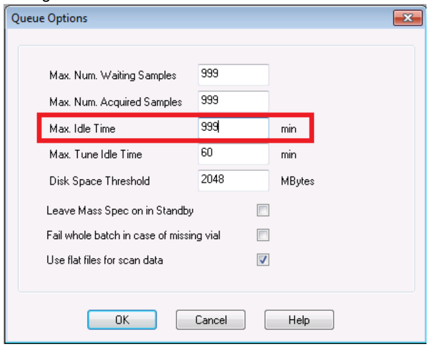


  - Click on the Acquire tab in the left column, click on "View Queue" icon
  - Click open file icon. Change the file type to "Acquisition Batch File (.dab)". click on the file "QC_MonthYear" to highlight and press OK.
  - Once the file opens, click on Sample Tab of the Batch List. Click "Add samples". Make sure the Data file Prefix field is blank and check mark only Set name. In the new samples number field enter "2" and click OK.

- Type the sample names "Blank_ddMMyy"and "SCRT_ddMMyy" where ddMMyy is the day, Month and Year.
  - Rack Code is "NA"
  - Rack Position is "0"
  - Plate Code: "NA"
  - Plate Position: "0"
  - Vial Position: "0"
  - Data File: "QC_MMYY"
  - Inj. Volume(ul): -1.000
- Method for the sample is "00_SCLipids_sMRM.dam".

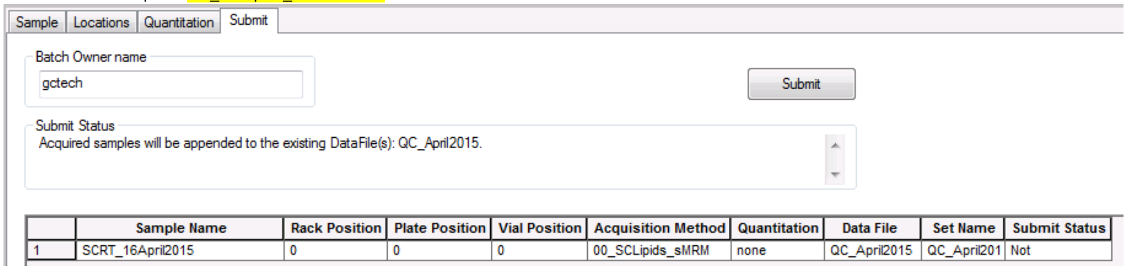


- (OPTIONAL) If a new column or Solution B has been added, a Blank first. Click on the row to highlight "Blank_ddMMYY" sample. Click on submit.
- Click on the submit tab. Click on the row to highlight "SCRT_ddMMYY" sample. Click on submit.
- Click "selected samples" then OK. On the Queue icon, confirm the sample is listed.

- 4.7 **PROCEDURE PART VII: Equilibrate 5500**
  - Click on Equilibrate icon.
  - Select the appropriate Acquisition Method, then set the equilibration time for 1 minute. Click OK. The gases and source temperature will reach operating values. After 1 minute, press the PLAY button.
    - No major changes to the system (no PM, no new column): **00_SCLipids_sMRM.dam**
    - Recent PM, new column, or peaks are shifted too far outside the window: **SCLipids New Column.dam**. Add "_chrom" to the SCRT_ddMMYY Sample Name.

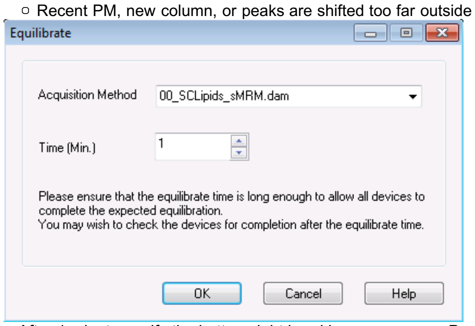


  - After 1 minute, verify the bottom right hand icons are green. Press the PLAY button.

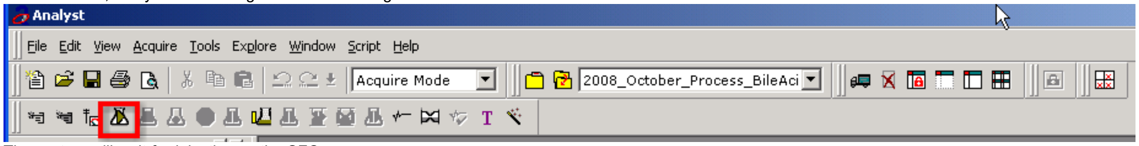


  - The system will wait for injection on the SFC.
  - Use the third button on the right side of the monitor to get to **Menu** then **Input Source**, then select **VGA** . The screen would now switch back to the MassLynx computer, where the Acquity Console can be accessed.

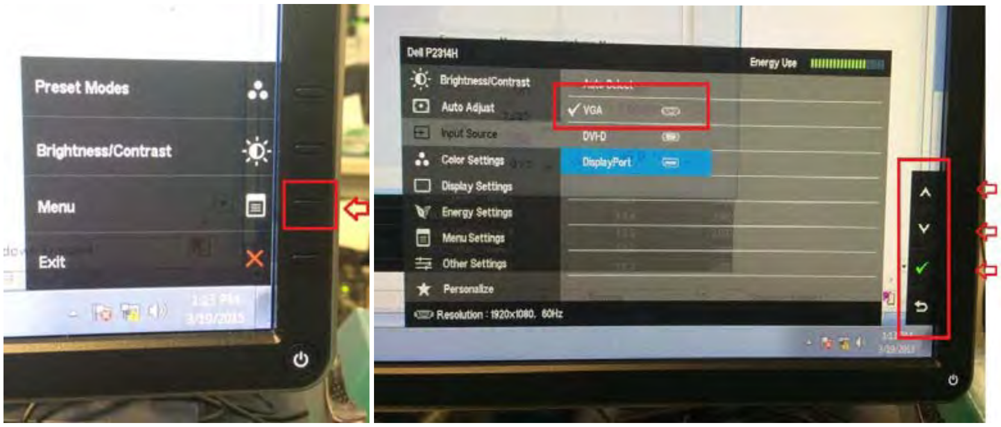


- 4.8 **PROCEDURE PART VIII: Run SCRT**
  - In the Acquity UPLC Console, after the start up system has completed (8min), the Regeneration Pump will turn on.
    - If the Regeneration Pump graph line is fluctuating, there's a pump issue. Troubleshooting is required

- 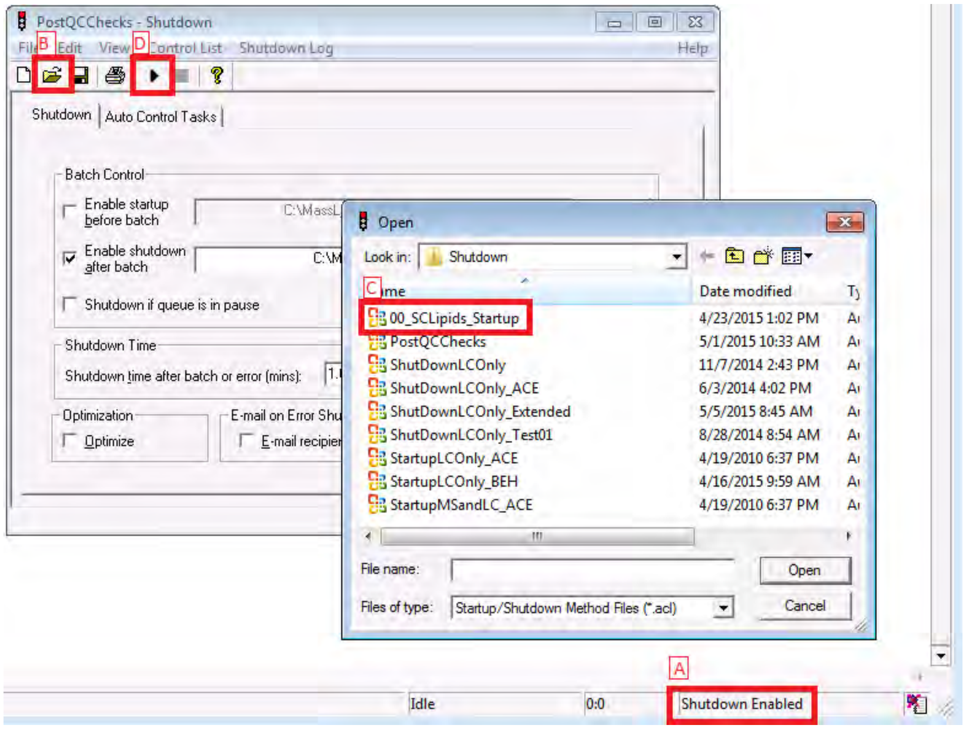

- In MassLynx, open the appropriate sample list (name QC Sample List.SPL)
- Check that the blank and SCRT vial positions are 1:A,1, and 1:F,8, respectively.
    - Inject Volume:2.000
- In the Acquity UPLC Console, wait till the Chromatographic Pump has a delta of < 20psi and has been stable for at least 2 minutes. Also make sure ABPR pressure has reached to 2000 psi.

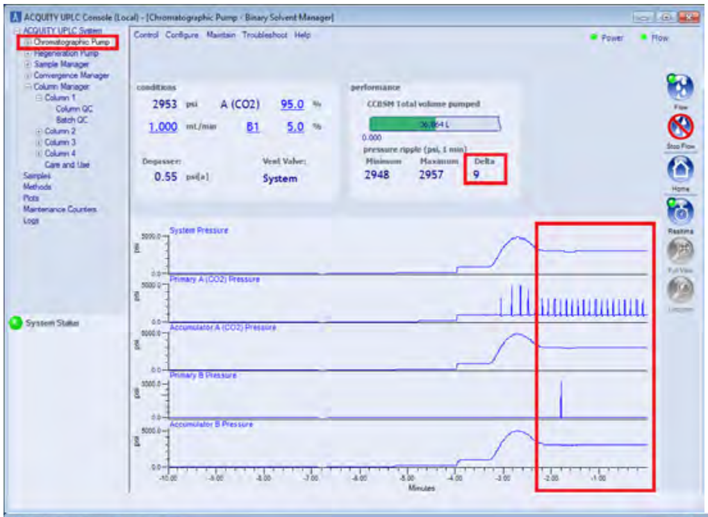


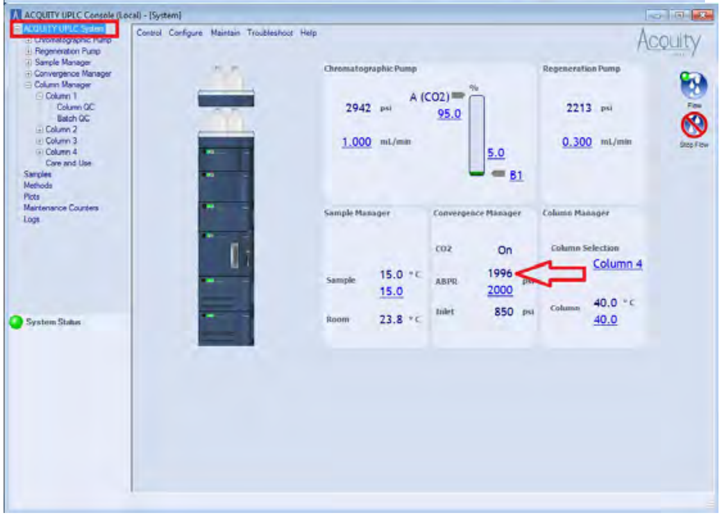


-

(OPTIONAL) Start the Blank

- Make sure Blank vial has been placed on position 1:A,1 on sample tray.
- Highlight the Blank row, press the PLAY button. Click on "Yes to all".

- Start the SCRT
  - Make sure SCRT sample has been placed on position 1:F,8 on sample tray. If there is icing on the sample cap, use a kimwipe to clean it before loading.
  - Highlight the SCRT row, press the PLAY button. Click on "Yes to all".
- In MassLynx, click on the bottom right corner of the sample list that says "Shutdow

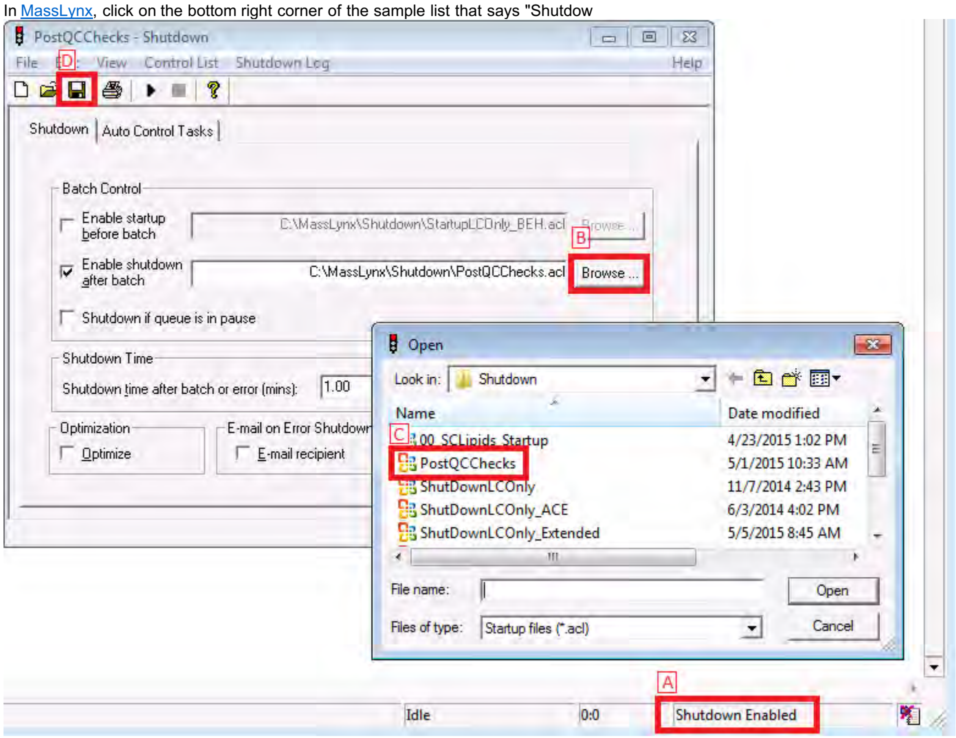


n enabled" [A]. Click BROWSE... [B], and select **PostQCChecks** [C] and save [D]. Close the window.
  - The BSM flow rate will decrease to 0.500ml/min. All other conditions will not change

- 4.10 **PROCEDURE PART X: Check RT**
  - In Analyst, open MultiQuant? 3.0 (or higher).
  - Select the current data path folder
  - Process the SCRT
    - Click the Wizard icon
    - Select QC_MonthYear from the available .wiff files
    - Double-click the current SCRT injection to move it over to the "Selected" window. Click
    - Next. Choose Method. Click Finish.
  - Check EOS peak S(C18)w30:0_18:2 for maintenance

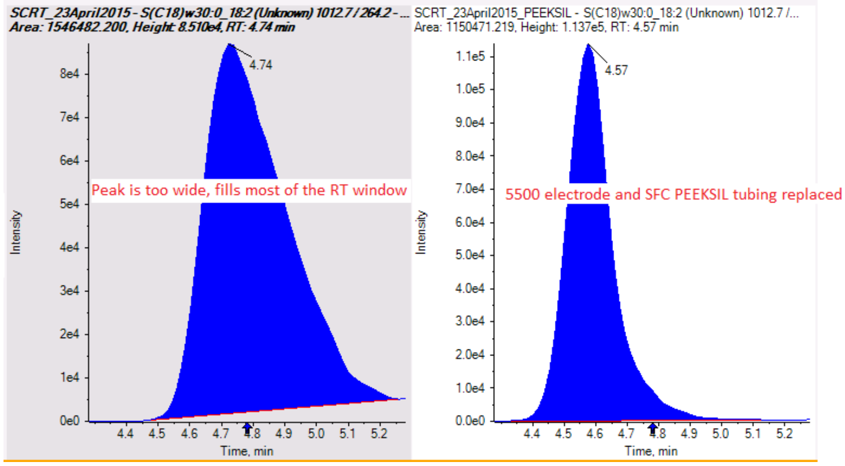


    - If poor peak shape is observed, run a Dynamic Leak Test on Pump A (then Pump B, ISM if suspected of leaking)
    - If Dynamic Leak Test(s) pass, replace the PEEKSIL tubing from the splitter to the source and the Electrode for the probe.

- Check that all peaks are assigned the appropriate RT.
  - Confirm ADS has the appropriate RT by comparing DS(C18)a24:0 and S(C18)a24:0. The two analytes should have very similar RTs (+/-0.05min).

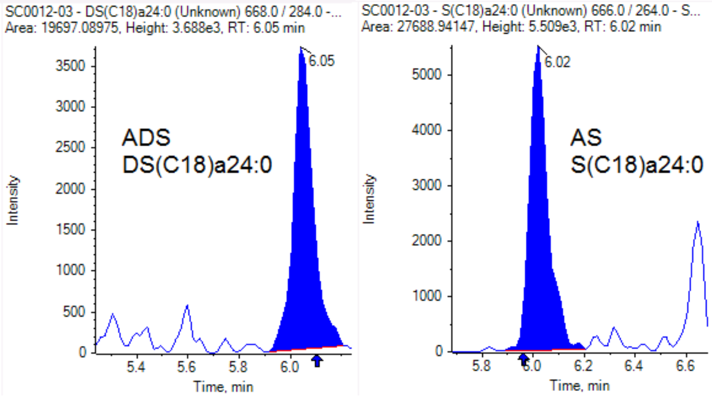


- Update RT, if needed, in the Acquisition Method.
  - In the Components & Groups window, select "All Components"
  - In the Table window, undo all previous sorting
  - Click edit, Copy Entire Table. Paste into Excel.
  - Copy the Retention Time for analytes with INDEX #1-342. Open the Analyst Acquisition Method: **00_SCLipids_sMRM.dam**. Click on the +MRM experiment and paste into the **Time (min)** column.

  - Copy the Retention Time for analytes with INDEX #343-359. Click on the -MRM experiment and paste into the **Time (min)** column.

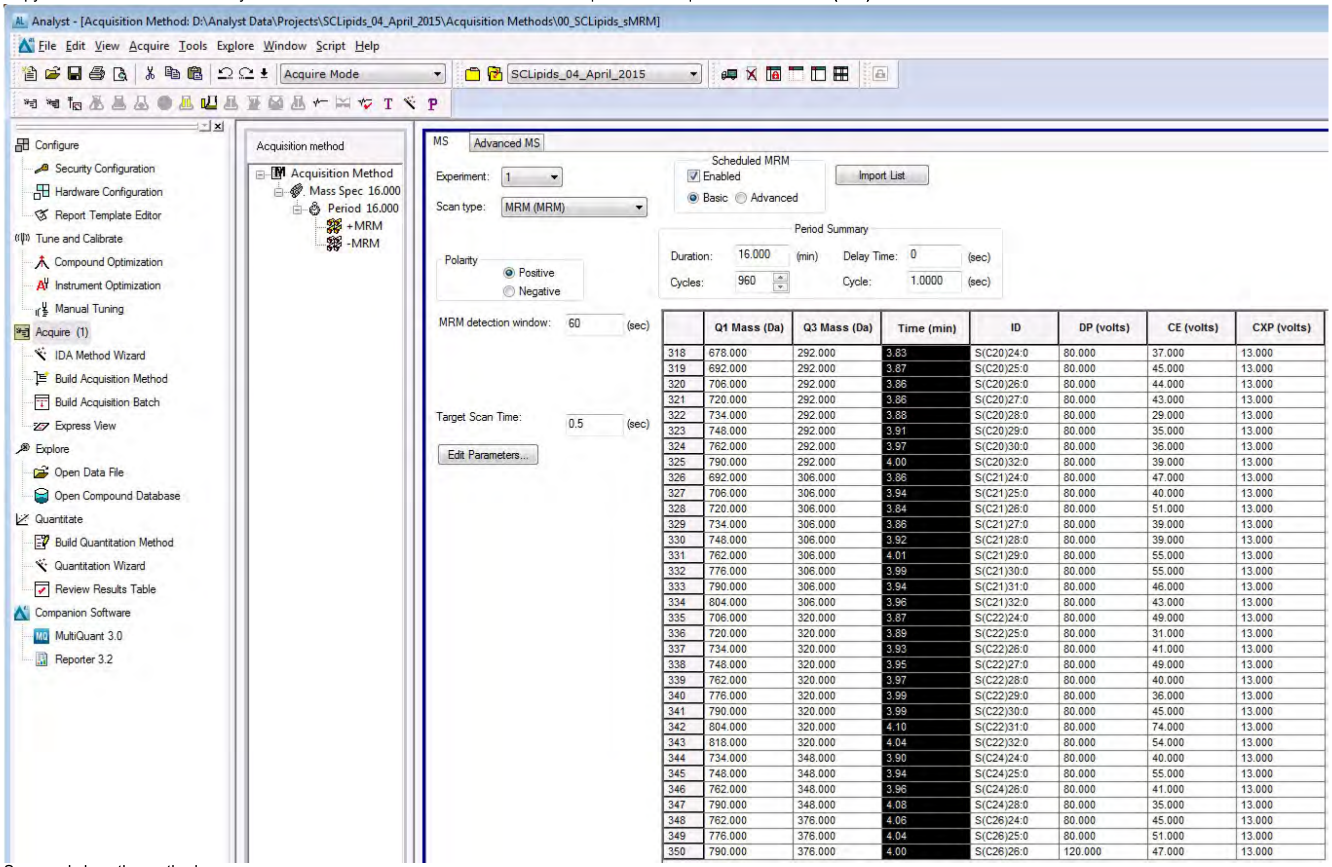


  - Save and close the method.
  - OPTIONAL: Re-inject SCRT to confirm

Software Version: Analyst 1.6.2

**Log Information from Devices at Start of acquisition:**

| | | | |
|---|---|---|---|
| Mass Spectrometer | QTRAP 5500 | 0 | SFC |
| Config Table Version | 04 | | |
| Firmware Version | ------- ------- PIL1400 PIB0101 | | |
| Component Name | Linear Ion Trap Quadrupole LC/MS/MS Mass Spectrometer | | |
| Component ID | QTRAP 5500 | | |
| Manufacturer | AB Sciex Instruments | | |
| Model | 1024945-BF | | |

| | | | | |
|---|---|---|---|---|
| Time from start =0.0167 min | Mass Spectrometer | QTRAP 5500 | 0 | SFC |
| Start of Run - Detailed Status | | | | |
| Vacuum Status | At Pressure | | | |
| Vacuum Gauge (10e-5 Torr) | 2.6 | | | |
| Backing Pump | Ok | | | |
| Interface Turbo Pump | Normal | | | |
| Analyzer Turbo Pump | Off | | | |
| Sample Introduction Status | Ready | | | |
| Source/Ion Path Electronics | On | | | |
| Source Type | Turbo Spray 451.0 | | | |
| Source Temperature (at setpoint) | C | | | |
| Source Exhaust Pump | On | | | |
| Injection Manifold | Bypass | | | |

| | | | | |
|---|---|---|---|---|
| Time from start =0.0167 min End of Run - Detailed Status Vacuum Status | Mass Spectrometer At Pressure | QTRAP 5500 | 0 | SFC |

| | |
|---|---|
| Vacuum Gauge (10e-5 Torr) | 2.5 |
| Backing Pump | Ok |
| Interface Turbo Pump | Normal |
| Analyzer Turbo Pump | Off |
| Sample Introduction Status | Ready |
| Source/Ion Path Electronics | On |
| Source Type | Turbo Spray |
| Source Temperature (at setpoint) | 451.0 C On |
| Source Exhaust Pump | Bypass |
| Injection Manifold | |

Time from start =27.4333 min

**Acquisition Info**

| | |
|---|---|
| Acquisition Method: | \00_SCLipids_sMRM.dam |
| Sample Acq Duration: | 16min0sec |
| Number of Scans: | 0 |
| Periods in File: | 1 |
| Synchronization Mode: | LC Sync |
| Auto-Equilibration: | Off |
| Software Version: Set | Analyst 1.6.2 |
| Name: | QC |
| Sample Name | SCRT |
| Autosampler Vial: | 0 |

| | |
|---|---|
| Rack Code: | N/A |
| Rack Position: | 0 |
| Plate Code: | N/A |
| Plate Position | 0 |

## Quantitation Information:

Dilution Factor: 1.000000

Custom Data:

Quantitation Table:

## Period 1:

--------------
Scans in Period: 1920
Relative Start Time: 0.00 msec
Experiments in Period: 2

## Period 1 Experiment 1:

----------------------------
Scan Type: MRM (MRM)
Scheduled MRM: Yes
Polarity: Positive
Scan Mode: N/A
Ion Source: Turbo Spray
MRM detection window: 60 sec
Target Scan Time: 0.5000 sec
Resolution Q1: Unit
Resolution Q3: Unit
Intensity Thres.: 0.00 cps
Settling Time: 50.0000 msec

| | |
|---|---|
| MR Pause: | 5.0070 msec |
| MCA: | No |
| Step Size: | 0.00 Da |

| Q1 Mass (Da) | Q3 Mass (Da) | Time (min) | Param | Start | Stop | ID |
|---|---|---|---|---|---|---|
| 370.000 | 264.000 | 3.65 | DP | 80.00 | 80.00 | S(C18)4:0 |
| | | | CE | 40.00 | 40.00 | |
| | | | CXP | 13.00 | 13.00 | |

| Q1 Mass (Da) | Q3 Mass (Da) | Time (min) | Param | Start | Stop | ID |
|---|---|---|---|---|---|---|
| 398.000 | 264.000 | 3.46 | DP | 80.00 | 80.00 | S(C18)6:0 |
| | | | CE | 40.00 | 40.00 | |
| | | | CXP | 13.00 | 13.00 | |

| Q1 Mass (Da) | Q3 Mass (Da) | Time (min) | Param | Start | Stop | ID |
|---|---|---|---|---|---|---|
| 426.000 | 264.000 | 3.46 | DP | 80.00 | 80.00 | S(C18)8:0 |
| | | | CE | 40.00 | 40.00 | |
| | | | CXP | 13.00 | 13.00 | |

| Q1 Mass (Da) | Q3 Mass (Da) | Time (min) | Param | Start | Stop | ID |
|---|---|---|---|---|---|---|
| 568.500 | 264.200 | 3.78 | DP | 61.00 | 61.00 | dS(C18)16:0 |
| | | | CE | 39.00 | 39.00 | |
| | | | CXP | 6.00 | 6.00 | |

| Q1 Mass (Da) | Q3 Mass (Da) | Time (min) | Param | Start | Stop | ID |
|---|---|---|---|---|---|---|
| 584.000 | 284.000 | 5.08 | DP | 80.00 | 80.00 | DS(C18)a18:0 |
| | | | CE | 45.00 | 45.00 | |
| | | | CXP | 13.00 | 13.00 | |

| Q1 Mass (Da) | Q3 Mass (Da) | Time (min) | Param | Start | Stop | ID |
|---|---|---|---|---|---|---|
| 668.000 | 284.000 | 5.21 | DP | 80.00 | 80.00 | DS(C18)a24:0 |
| | | | CE | 42.00 | 42.00 | |
| | | | CXP | 13.00 | 13.00 | |

| Q1 Mass (Da) | Q3 Mass (Da) | Time (min) | Param | Start | Stop | ID |
|---|---|---|---|---|---|---|
| 682.000 | 284.000 | 5.22 | DP | 80.00 | 80.00 | DS(C18)a25:0 |
| | | | CE | 45.00 | 45.00 | |
| | | | CXP | 13.00 | 13.00 | |

| Q1 Mass (Da) | Q3 Mass (Da) | Time (min) | Param | Start | Stop | ID |
|---|---|---|---|---|---|---|
| 696.000 | 284.000 | 5.22 | DP | 80.00 | 80.00 | DS(C18)a26:0 |
| | | | CE | 42.00 | 42.00 | |
| | | | CXP | 13.00 | 13.00 | |

| Q1 Mass (Da) | Q3 Mass (Da) | Time (min) | Param | Start | Stop | ID |
|---|---|---|---|---|---|---|
| 696.000 | 312.000 | 5.28 | DP | 80.00 | 80.00 | DS(C20)a24:0 |
| | | | CE | 46.00 | 46.00 | |
| | | | CXP | 13.00 | 13.00 | |

| Q1 Mass (Da) | Q3 Mass (Da) | Time (min) | Param | Start | Stop | ID |
|---|---|---|---|---|---|---|
| 710.000 | 312.000 | 5.30 | DP | 80.00 | 80.00 | DS(C20)a25:0 |
| | | | CE | 45.00 | 45.00 | |
| | | | CXP | 13.00 | 13.00 | |

| Q1 Mass (Da) | Q3 Mass (Da) | Time (min) | Param | Start | Stop | ID |
|---|---|---|---|---|---|---|
| 724.000 | 312.000 | 5.32 | DP | 80.00 | 80.00 | DS(C20)a26:0 |
| | | | CE | 40.00 | 40.00 | |
| | | | CXP | 13.00 | 13.00 | |

| Q1 Mass (Da) | Q3 Mass (Da) | Time (min) | Param | Start | Stop | ID |
|---|---|---|---|---|---|---|
| 636.000 | 252.000 | 6.71 | DP | 80.00 | 80.00 | H(C16)a24:0 |
| | | | CE | 36.00 | 36.00 | |
| | | | CXP | 13.00 | 13.00 | |

| Q1 Mass (Da) | Q3 Mass (Da) | Time (min) | Param | Start | Stop | ID |
|---|---|---|---|---|---|---|
| 650.000 | 252.000 | 6.72 | DP | 80.00 | 80.00 | H(C16)a25:0 |
| | | | CE | 33.00 | 33.00 | |
| | | | CXP | 13.00 | 13.00 | |

| Q1 Mass (Da) | Q3 Mass (Da) | Time (min) | Param | Start | Stop | ID |
|---|---|---|---|---|---|---|
| 664.000 | 252.000 | 6.77 | DP | 80.00 | 80.00 | H(C16)a26:0 |
| | | | CE | 75.00 | 75.00 | |
| | | | CXP | 13.00 | 13.00 | |

| Q1 Mass (Da) | Q3 Mass (Da) | Time (min) | Param | Start | Stop | ID |
|---|---|---|---|---|---|---|
| 678.000 | 252.000 | 6.76 | DP | 80.00 | 80.00 | H(C16)a27:0 |
| | | | CE | 36.00 | 36.00 | |
| | | | CXP | 13.00 | 13.00 | |

| Q1 Mass (Da) | Q3 Mass (Da) | Time (min) | Param | Start | Stop | ID |
|---|---|---|---|---|---|---|
| 692.000 | 252.000 | 6.80 | DP | 80.00 | 80.00 | H(C16)a28:0 |
| | | | CE | 45.00 | 45.00 | |
| | | | CXP | 13.00 | 13.00 | |

| Q1 Mass (Da) | Q3 Mass (Da) | Time (min) | Param | Start | Stop | ID |
|---|---|---|---|---|---|---|
| 650.000 | 266.000 | 6.74 | DP | 80.00 | 80.00 | H(C17)a24:0 |
| | | | CE | 31.00 | 31.00 | |
| | | | CXP | 13.00 | 13.00 | |

| Q1 Mass (Da) | Q3 Mass (Da) | Time (min) | Param | Start | Stop | ID |
|---|---|---|---|---|---|---|
| 664.000 | 266.000 | 6.75 | DP | 80.00 | 80.00 | H(C17)a25:0 |
| | | | CE | 29.00 | 29.00 | |
| | | | CXP | 13.00 | 13.00 | |

| Q1 Mass (Da) | Q3 Mass (Da) | Time (min) | Param | Start | Stop | ID |
|---|---|---|---|---|---|---|
| 678.000 | 266.000 | 6.76 | DP | 80.00 | 80.00 | H(C17)a26:0 |
| | | | CE | 35.00 | 35.00 | |
| | | | CXP | 13.00 | 13.00 | |

| Q1 Mass (Da) | Q3 Mass (Da) | Time (min) | Param | Start | Stop | ID |
|---|---|---|---|---|---|---|
| 692.000 | 266.000 | 6.83 | DP | 80.00 | 80.00 | H(C17)a27:0 |
| | | | CE | 53.00 | 53.00 | |
| | | | CXP | 13.00 | 13.00 | |

| Q1 Mass (Da) | Q3 Mass (Da) | Time (min) | Param | Start | Stop | ID |
|---|---|---|---|---|---|---|
| 720.000 | 266.000 | 6.89 | DP | 80.00 | 80.00 | H(C17)a29:0 |
| | | | CE | 44.00 | 44.00 | |
| | | | CXP | 13.00 | 13.00 | |

| Q1 Mass (Da) | Q3 Mass (Da) | Time (min) | Param | Start | Stop | ID |
|---|---|---|---|---|---|---|
| 608.000 | 280.000 | 6.73 | DP | 80.00 | 80.00 | H(C18)a20:0 |
| | | | CE | 44.00 | 44.00 | |
| | | | CXP | 13.00 | 13.00 | |

| Q1 Mass (Da) | Q3 Mass (Da) | Time (min) | Param | Start | Stop | ID |
|---|---|---|---|---|---|---|
| 636.000 | 280.000 | 6.76 | DP | 80.00 | 80.00 | H(C18)a22:0 |
| | | | CE | 37.00 | 37.00 | |
| | | | CXP | 13.00 | 13.00 | |

| Q1 Mass (Da) | Q3 Mass (Da) | Time (min) | Param | Start | Stop | ID |
|---|---|---|---|---|---|---|
| 664.000 | 280.000 | 6.78 | DP | 80.00 | 80.00 | H(C18)a24:0 |
| | | | CE | 34.00 | 34.00 | |
| | | | CXP | 13.00 | 13.00 | |

| Q1 Mass (Da) | Q3 Mass (Da) | Time (min) | Param | Start | Stop | ID |
|---|---|---|---|---|---|---|
| 678.000 | 280.000 | 6.80 | DP | 80.00 | 80.00 | H(C18)a25:0 |
| | | | CE | 34.00 | 34.00 | |
| | | | CXP | 13.00 | 13.00 | |

| Q1 Mass (Da) | Q3 Mass (Da) | Time (min) | Param | Start | Stop | ID |
|---|---|---|---|---|---|---|

| Q1 Mass (Da) | Q3 Mass (Da) | Time (min) | Param | Start | Stop | ID |
|---|---|---|---|---|---|---|
| 692.000 | 280.000 | 6.80 | DP | 80.00 | 80.00 | H(C18)a26:0 |
| | | | CE | 38.00 | 38.00 | |
| | | | CXP | 13.00 | 13.00 | |

| Q1 Mass (Da) | Q3 Mass (Da) | Time (min) | Param | Start | Stop | ID |
|---|---|---|---|---|---|---|
| 706.000 | 280.000 | 6.81 | DP | 80.00 | 80.00 | H(C18)a27:0 |
| | | | CE | 35.00 | 35.00 | |
| | | | CXP | 13.00 | 13.00 | |

| Q1 Mass (Da) | Q3 Mass (Da) | Time (min) | Param | Start | Stop | ID |
|---|---|---|---|---|---|---|
| 720.000 | 280.000 | 6.83 | DP | 80.00 | 80.00 | H(C18)a28:0 |
| | | | CE | 35.00 | 35.00 | |
| | | | CXP | 13.00 | 13.00 | |

| Q1 Mass (Da) | Q3 Mass (Da) | Time (min) | Param | Start | Stop | ID |
|---|---|---|---|---|---|---|
| 678.000 | 294.000 | 6.82 | DP | 80.00 | 80.00 | H(C19)a24:0 |
| | | | CE | 33.00 | 33.00 | |
| | | | CXP | 13.00 | 13.00 | |

| Q1 Mass (Da) | Q3 Mass (Da) | Time (min) | Param | Start | Stop | ID |
|---|---|---|---|---|---|---|
| 692.000 | 294.000 | 6.83 | DP | 80.00 | 80.00 | H(C19)a25:0 |
| | | | CE | 34.00 | 34.00 | |
| | | | CXP | 13.00 | 13.00 | |

| Q1 Mass (Da) | Q3 Mass (Da) | Time (min) | Param | Start | Stop | ID |
|---|---|---|---|---|---|---|
| 706.000 | 294.000 | 6.85 | DP | 80.00 | 80.00 | H(C19)a26:0 |

| | | | | | | |
|---|---|---|---|---|---|---|
| | | | CE | 34.00 | 34.00 | |
| | | | CXP | 13.00 | 13.00 | |

| Q1 Mass (Da) | Q3 Mass (Da) | Time (min) | Param | Start | Stop | ID |
|---|---|---|---|---|---|---|
| 692.000 | 308.000 | 6.86 | DP | 80.00 | 80.00 | H(C20)a24:0 |
| | | | CE | 36.00 | 36.00 | |
| | | | CXP | 13.00 | 13.00 | |

| Q1 Mass (Da) | Q3 Mass (Da) | Time (min) | Param | Start | Stop | ID |
|---|---|---|---|---|---|---|
| 706.000 | 308.000 | 6.87 | DP | 80.00 | 80.00 | H(C20)a25:0 |
| | | | CE | 34.00 | 34.00 | |
| | | | CXP | 13.00 | 13.00 | |

| Q1 Mass (Da) | Q3 Mass (Da) | Time (min) | Param | Start | Stop | ID |
|---|---|---|---|---|---|---|
| 720.000 | 308.000 | 6.88 | DP | 80.00 | 80.00 | H(C20)a26:0 |
| | | | CE | 35.00 | 35.00 | |
| | | | CXP | 13.00 | 13.00 | |

| Q1 Mass (Da) | Q3 Mass (Da) | Time (min) | Param | Start | Stop | ID |
|---|---|---|---|---|---|---|
| 748.000 | 308.000 | 6.91 | DP | 80.00 | 80.00 | H(C20)a28:0 |
| | | | CE | 34.00 | 34.00 | |
| | | | CXP | 13.00 | 13.00 | |

| Q1 Mass (Da) | Q3 Mass (Da) | Time (min) | Param | Start | Stop | ID |
|---|---|---|---|---|---|---|
| 706.000 | 322.000 | 6.89 | DP | 80.00 | 80.00 | H(C21)a24:0 |
| | | | CE | 31.00 | 31.00 | |

| Q1 Mass (Da) | Q3 Mass (Da) | Time (min) | Param | Start | Stop | ID |
|---|---|---|---|---|---|---|
| | | | CXP | 13.00 | 13.00 | |

| Q1 Mass (Da) | Q3 Mass (Da) | Time (min) | Param | Start | Stop | ID |
|---|---|---|---|---|---|---|
| 720.000 | 322.000 | 6.91 | DP | 80.00 | 80.00 | H(C21)a25:0 |
| | | | CE | 44.00 | 44.00 | |
| | | | CXP | 13.00 | 13.00 | |

| Q1 Mass (Da) | Q3 Mass (Da) | Time (min) | Param | Start | Stop | ID |
|---|---|---|---|---|---|---|
| 734.000 | 322.000 | 6.91 | DP | 80.00 | 80.00 | H(C21)a26:0 |
| | | | CE | 32.00 | 32.00 | |
| | | | CXP | 13.00 | 13.00 | |

| Q1 Mass (Da) | Q3 Mass (Da) | Time (min) | Param | Start | Stop | ID |
|---|---|---|---|---|---|---|
| 608.000 | 336.000 | 6.75 | DP | 80.00 | 80.00 | H(C22)a16:0 |
| | | | CE | 40.00 | 40.00 | |
| | | | CXP | 13.00 | 13.00 | |

| Q1 Mass (Da) | Q3 Mass (Da) | Time (min) | Param | Start | Stop | ID |
|---|---|---|---|---|---|---|
| 720.000 | 336.000 | 6.91 | DP | 80.00 | 80.00 | H(C22)a24:0 |
| | | | CE | 44.00 | 44.00 | |
| | | | CXP | 13.00 | 13.00 | |

| Q1 Mass (Da) | Q3 Mass (Da) | Time (min) | Param | Start | Stop | ID |
|---|---|---|---|---|---|---|
| 734.000 | 336.000 | 6.93 | DP | 80.00 | 80.00 | H(C22)a25:0 |
| | | | CE | 35.00 | 35.00 | |
| | | | CXP | 13.00 | 13.00 | |

| Q1 Mass (Da) | Q3 Mass (Da) | Time (min) | Param | Start | Stop | ID |
|---|---|---|---|---|---|---|
| 748.000 | 336.000 | 6.94 | DP | 80.00 | 80.00 | H(C22)a26:0 |
| | | | CE | 40.00 | 40.00 | |
| | | | CXP | 13.00 | 13.00 | |

| Q1 Mass (Da) | Q3 Mass (Da) | Time (min) | Param | Start | Stop | ID |
|---|---|---|---|---|---|---|
| 656.000 | 254.000 | 6.01 | DP | 50.00 | 50.00 | P(C16)a24:0 |
| | | | CE | 49.00 | 49.00 | |
| | | | CXP | 14.00 | 14.00 | |

| Q1 Mass (Da) | Q3 Mass (Da) | Time (min) | Param | Start | Stop | ID |
|---|---|---|---|---|---|---|
| 670.000 | 254.000 | 6.02 | DP | 50.00 | 50.00 | P(C16)a25:0 |
| | | | CE | 49.00 | 49.00 | |
| | | | CXP | 14.00 | 14.00 | |

| Q1 Mass (Da) | Q3 Mass (Da) | Time (min) | Param | Start | Stop | ID |
|---|---|---|---|---|---|---|
| 684.000 | 254.000 | 6.03 | DP | 50.00 | 50.00 | P(C16)a26:0 |
| | | | CE | 49.00 | 49.00 | |
| | | | CXP | 14.00 | 14.00 | |

| Q1 Mass (Da) | Q3 Mass (Da) | Time (min) | Param | Start | Stop | ID |
|---|---|---|---|---|---|---|
| 670.000 | 268.000 | 6.04 | DP | 50.00 | 50.00 | P(C17)a24:0 |
| | | | CE | 43.00 | 43.00 | |
| | | | CXP | 14.00 | 14.00 | |

| Q1 Mass (Da) | Q3 Mass (Da) | Time (min) | Param | Start | Stop | ID |
|---|---|---|---|---|---|---|
| 684.000 | 268.000 | 6.06 | DP | 50.00 | 50.00 | P(C17)a25:0 |
| | | | CE | 45.00 | 45.00 | |
| | | | CXP | 14.00 | 14.00 | |

| Q1 Mass (Da) | Q3 Mass (Da) | Time (min) | Param | Start | Stop | ID |
|---|---|---|---|---|---|---|
| 698.000 | 268.000 | 6.07 | DP | 50.00 | 50.00 | P(C17)a26:0 |
| | | | CE | 39.00 | 39.00 | |
| | | | CXP | 14.00 | 14.00 | |

| Q1 Mass (Da) | Q3 Mass (Da) | Time (min) | Param | Start | Stop | ID |
|---|---|---|---|---|---|---|
| 600.000 | 282.000 | 5.97 | DP | 50.00 | 50.00 | P(C18)a18:0 |
| | | | CE | 30.00 | 30.00 | |
| | | | CXP | 14.00 | 14.00 | |

| Q1 Mass (Da) | Q3 Mass (Da) | Time (min) | Param | Start | Stop | ID |
|---|---|---|---|---|---|---|
| 628.000 | 282.000 | 6.01 | DP | 50.00 | 50.00 | P(C18)a20:0 |
| | | | CE | 36.00 | 36.00 | |
| | | | CXP | 14.00 | 14.00 | |

| Q1 Mass (Da) | Q3 Mass (Da) | Time (min) | Param | Start | Stop | ID |
|---|---|---|---|---|---|---|
| 656.000 | 282.000 | 6.05 | DP | 50.00 | 50.00 | P(C18)a22:0 |
| | | | CE | 44.00 | 44.00 | |
| | | | CXP | 14.00 | 14.00 | |

| Q1 Mass (Da) | Q3 Mass (Da) | Time (min) | Param | Start | Stop | ID |
|---|---|---|---|---|---|---|
| 670.000 | 282.000 | 6.06 | DP | 50.00 | 50.00 | P(C18)a23:0 |
| | | | CE | 40.00 | 40.00 | |
| | | | CXP | 14.00 | 14.00 | |

| Q1 Mass (Da) | Q3 Mass (Da) | Time (min) | Param | Start | Stop | ID |
|---|---|---|---|---|---|---|
| 684.000 | 264.200 | 6.08 | DP | 50.00 | 50.00 | P(C18)a24:0 |
| | | | CE | 45.00 | 45.00 | |
| | | | CXP | 14.00 | 14.00 | |

| Q1 Mass (Da) | Q3 Mass (Da) | Time (min) | Param | Start | Stop | ID |
|---|---|---|---|---|---|---|
| 698.000 | 282.000 | 6.09 | DP | 50.00 | 50.00 | P(C18)a25:0 |
| | | | CE | 45.00 | 45.00 | |
| | | | CXP | 14.00 | 14.00 | |

| Q1 Mass (Da) | Q3 Mass (Da) | Time (min) | Param | Start | Stop | ID |
|---|---|---|---|---|---|---|
| 712.000 | 282.000 | 6.10 | DP | 50.00 | 50.00 | P(C18)a26:0 |
| | | | CE | 40.00 | 40.00 | |
| | | | CXP | 14.00 | 14.00 | |

| Q1 Mass (Da) | Q3 Mass (Da) | Time (min) | Param | Start | Stop | ID |
|---|---|---|---|---|---|---|
| 726.000 | 282.000 | 6.14 | DP | 50.00 | 50.00 | P(C18)a27:0 |
| | | | CE | 49.00 | 49.00 | |
| | | | CXP | 14.00 | 14.00 | |

| Q1 Mass (Da) | Q3 Mass (Da) | Time (min) | Param | Start | Stop | ID |
|---|---|---|---|---|---|---|
| 740.000 | 282.000 | 6.15 | DP | 50.00 | 50.00 | P(C18)a28:0 |
| | | | CE | 48.00 | 48.00 | |
| | | | CXP | 14.00 | 14.00 | |

| Q1 Mass (Da) | Q3 Mass (Da) | Time (min) | Param | Start | Stop | ID |
|---|---|---|---|---|---|---|
| 698.000 | 296.000 | 6.12 | DP | 50.00 | 50.00 | P(C19)a24:0 |
| | | | CE | 42.00 | 42.00 | |
| | | | CXP | 14.00 | 14.00 | |

| Q1 Mass (Da) | Q3 Mass (Da) | Time (min) | Param | Start | Stop | ID |
|---|---|---|---|---|---|---|
| 712.000 | 296.000 | 6.12 | DP | 50.00 | 50.00 | P(C19)a25:0 |
| | | | CE | 40.00 | 40.00 | |
| | | | CXP | 14.00 | 14.00 | |

| Q1 Mass (Da) | Q3 Mass (Da) | Time (min) | Param | Start | Stop | ID |
|---|---|---|---|---|---|---|
| 726.000 | 296.000 | 6.13 | DP | 50.00 | 50.00 | P(C19)a26:0 |
| | | | CE | 49.00 | 49.00 | |
| | | | CXP | 14.00 | 14.00 | |

| Q1 Mass (Da) | Q3 Mass (Da) | Time (min) | Param | Start | Stop | ID |
|---|---|---|---|---|---|---|
| 656.000 | 310.000 | 6.07 | DP | 50.00 | 50.00 | P(C20)a20:0 |
| | | | CE | 44.00 | 44.00 | |
| | | | CXP | 14.00 | 14.00 | |

| Q1 Mass (Da) | Q3 Mass (Da) | Time (min) | Param | Start | Stop | ID |
|---|---|---|---|---|---|---|

| | | | | | | |
|---|---|---|---|---|---|---|
| 684.000 | 310.000 | 6.11 | DP | 50.00 | 50.00 | P(C20)a22:0 |
| | | | CE | 49.00 | 49.00 | |
| | | | CXP | 14.00 | 14.00 | |

| Q1 Mass (Da) | Q3 Mass (Da) | Time (min) | Param | Start | Stop | ID |
|---|---|---|---|---|---|---|
| 698.000 | 310.000 | 6.12 | DP | 50.00 | 50.00 | P(C20)a23:0 |
| | | | CE | 41.00 | 41.00 | |
| | | | CXP | 14.00 | 14.00 | |

| Q1 Mass (Da) | Q3 Mass (Da) | Time (min) | Param | Start | Stop | ID |
|---|---|---|---|---|---|---|
| 712.000 | 310.000 | 6.14 | DP | 50.00 | 50.00 | P(C20)a24:0 |
| | | | CE | 40.00 | 40.00 | |
| | | | CXP | 14.00 | 14.00 | |

| Q1 Mass (Da) | Q3 Mass (Da) | Time (min) | Param | Start | Stop | ID |
|---|---|---|---|---|---|---|
| 726.000 | 310.000 | 6.16 | DP | 50.00 | 50.00 | P(C20)a25:0 |
| | | | CE | 46.00 | 46.00 | |
| | | | CXP | 14.00 | 14.00 | |

| Q1 Mass (Da) | Q3 Mass (Da) | Time (min) | Param | Start | Stop | ID |
|---|---|---|---|---|---|---|
| 740.000 | 310.000 | 6.18 | DP | 50.00 | 50.00 | P(C20)a26:0 |
| | | | CE | 45.00 | 45.00 | |
| | | | CXP | 14.00 | 14.00 | |

| Q1 Mass (Da) | Q3 Mass (Da) | Time (min) | Param | Start | Stop | ID |
|---|---|---|---|---|---|---|
| 726.000 | 324.000 | 6.17 | DP | 50.00 | 50.00 | P(C21)a24:0 |

| | | | | | | |
|---|---|---|---|---|---|---|
| | | | CE | 40.00 | 40.00 | |
| | | | CXP | 14.00 | 14.00 | |

| Q1 Mass (Da) | Q3 Mass (Da) | Time (min) | Param | Start | Stop | ID |
|---|---|---|---|---|---|---|
| 740.000 | 324.000 | 6.20 | DP | 50.00 | 50.00 | P(C21)a25:0 |
| | | | CE | 55.00 | 55.00 | |
| | | | CXP | 14.00 | 14.00 | |

| Q1 Mass (Da) | Q3 Mass (Da) | Time (min) | Param | Start | Stop | ID |
|---|---|---|---|---|---|---|
| 754.000 | 324.000 | 6.20 | DP | 50.00 | 50.00 | P(C21)a26:0 |
| | | | CE | 40.00 | 40.00 | |
| | | | CXP | 14.00 | 14.00 | |

| Q1 Mass (Da) | Q3 Mass (Da) | Time (min) | Param | Start | Stop | ID |
|---|---|---|---|---|---|---|
| 712.000 | 338.000 | 6.16 | DP | 50.00 | 50.00 | P(C22)a22:0 |
| | | | CE | 45.00 | 45.00 | |
| | | | CXP | 14.00 | 14.00 | |

| Q1 Mass (Da) | Q3 Mass (Da) | Time (min) | Param | Start | Stop | ID |
|---|---|---|---|---|---|---|
| 740.000 | 338.000 | 6.21 | DP | 50.00 | 50.00 | P(C22)a24:0 |
| | | | CE | 37.00 | 37.00 | |
| | | | CXP | 14.00 | 14.00 | |

| Q1 Mass (Da) | Q3 Mass (Da) | Time (min) | Param | Start | Stop | ID |
|---|---|---|---|---|---|---|
| 754.000 | 338.000 | 6.23 | DP | 50.00 | 50.00 | P(C22)a25:0 |
| | | | CE | 45.00 | 45.00 | |

| | | | | | | |
|---|---|---|---|---|---|---|
| | | | CXP | 14.00 | 14.00 | |

| Q1 Mass (Da) | Q3 Mass (Da) | Time (min) | Param | Start | Stop | ID |
|---|---|---|---|---|---|---|
| 768.000 | 338.000 | 6.25 | DP | 50.00 | 50.00 | P(C22)a26:0 |
| | | | CE | 50.00 | 50.00 | |
| | | | CXP | 14.00 | 14.00 | |

| Q1 Mass (Da) | Q3 Mass (Da) | Time (min) | Param | Start | Stop | ID |
|---|---|---|---|---|---|---|
| 638.000 | 236.000 | 5.14 | DP | 80.00 | 80.00 | S(C16)a24:0 |
| | | | CE | 37.00 | 37.00 | |
| | | | CXP | 13.00 | 13.00 | |

| Q1 Mass (Da) | Q3 Mass (Da) | Time (min) | Param | Start | Stop | ID |
|---|---|---|---|---|---|---|
| 652.000 | 236.000 | 5.16 | DP | 80.00 | 80.00 | S(C16)a25:0 |
| | | | CE | 44.00 | 44.00 | |
| | | | CXP | 13.00 | 13.00 | |

| Q1 Mass (Da) | Q3 Mass (Da) | Time (min) | Param | Start | Stop | ID |
|---|---|---|---|---|---|---|
| 666.000 | 236.000 | 5.17 | DP | 80.00 | 80.00 | S(C16)a26:0 |
| | | | CE | 40.00 | 40.00 | |
| | | | CXP | 13.00 | 13.00 | |

| Q1 Mass (Da) | Q3 Mass (Da) | Time (min) | Param | Start | Stop | ID |
|---|---|---|---|---|---|---|
| 680.000 | 236.000 | 5.17 | DP | 80.00 | 80.00 | S(C16)a27:0 |
| | | | CE | 37.00 | 37.00 | |
| | | | CXP | 13.00 | 13.00 | |

| Q1 Mass (Da) | Q3 Mass (Da) | Time (min) | Param | Start | Stop | ID |
|---|---|---|---|---|---|---|
| 694.000 | 236.000 | 5.19 | DP | 80.00 | 80.00 | S(C16)a28:0 |
| | | | CE | 44.00 | 44.00 | |
| | | | CXP | 13.00 | 13.00 | |

| Q1 Mass (Da) | Q3 Mass (Da) | Time (min) | Param | Start | Stop | ID |
|---|---|---|---|---|---|---|
| 652.000 | 250.000 | 5.18 | DP | 80.00 | 80.00 | S(C17)a24:0 |
| | | | CE | 34.00 | 34.00 | |
| | | | CXP | 13.00 | 13.00 | |

| Q1 Mass (Da) | Q3 Mass (Da) | Time (min) | Param | Start | Stop | ID |
|---|---|---|---|---|---|---|
| 666.000 | 250.000 | 5.20 | DP | 80.00 | 80.00 | S(C17)a25:0 |
| | | | CE | 34.00 | 34.00 | |
| | | | CXP | 13.00 | 13.00 | |

| Q1 Mass (Da) | Q3 Mass (Da) | Time (min) | Param | Start | Stop | ID |
|---|---|---|---|---|---|---|
| 680.000 | 250.000 | 5.21 | DP | 80.00 | 80.00 | S(C17)a26:0 |
| | | | CE | 39.00 | 39.00 | |
| | | | CXP | 13.00 | 13.00 | |

| Q1 Mass (Da) | Q3 Mass (Da) | Time (min) | Param | Start | Stop | ID |
|---|---|---|---|---|---|---|
| 694.000 | 250.000 | 5.25 | DP | 80.00 | 80.00 | S(C17)a27:0 |
| | | | CE | 45.00 | 45.00 | |
| | | | CXP | 13.00 | 13.00 | |

| Q1 Mass (Da) | Q3 Mass (Da) | Time (min) | Param | Start | Stop | ID |
|---|---|---|---|---|---|---|
| 708.000 | 250.000 | 5.23 | DP | 80.00 | 80.00 | S(C17)a28:0 |
| | | | CE | 40.00 | 40.00 | |
| | | | CXP | 13.00 | 13.00 | |

| Q1 Mass (Da) | Q3 Mass (Da) | Time (min) | Param | Start | Stop | ID |
|---|---|---|---|---|---|---|
| 568.000 | 264.000 | 5.08 | DP | 80.00 | 80.00 | S(C18)a17:0 |
| | | | CE | 35.00 | 35.00 | |
| | | | CXP | 13.00 | 13.00 | |

| Q1 Mass (Da) | Q3 Mass (Da) | Time (min) | Param | Start | Stop | ID |
|---|---|---|---|---|---|---|
| 582.500 | 264.200 | 5.10 | DP | 50.00 | 50.00 | S(C18)a18:0 |
| | | | CE | 39.00 | 39.00 | |
| | | | CXP | 18.00 | 18.00 | |

| Q1 Mass (Da) | Q3 Mass (Da) | Time (min) | Param | Start | Stop | ID |
|---|---|---|---|---|---|---|
| 610.200 | 264.300 | 5.13 | DP | 41.00 | 41.00 | S(C18)a20:0 |
| | | | CE | 45.00 | 45.00 | |
| | | | CXP | 18.00 | 18.00 | |

| Q1 Mass (Da) | Q3 Mass (Da) | Time (min) | Param | Start | Stop | ID |
|---|---|---|---|---|---|---|
| 638.800 | 264.200 | 5.19 | DP | 21.00 | 21.00 | S(C18)a22:0 |
| | | | CE | 49.00 | 49.00 | |
| | | | CXP | 14.00 | 14.00 | |

| Q1 Mass (Da) | Q3 Mass (Da) | Time (min) | Param | Start | Stop | ID |
|---|---|---|---|---|---|---|
| 652.000 | 264.000 | 5.20 | DP | 80.00 | 80.00 | S(C18)a23:0 |
| | | | CE | 36.00 | 36.00 | |
| | | | CXP | 13.00 | 13.00 | |

| Q1 Mass (Da) | Q3 Mass (Da) | Time (min) | Param | Start | Stop | ID |
|---|---|---|---|---|---|---|
| 666.000 | 264.000 | 5.21 | DP | 80.00 | 80.00 | S(C18)a24:0 |
| | | | CE | 31.00 | 31.00 | |
| | | | CXP | 13.00 | 13.00 | |

| Q1 Mass (Da) | Q3 Mass (Da) | Time (min) | Param | Start | Stop | ID |
|---|---|---|---|---|---|---|
| 680.000 | 264.000 | 5.24 | DP | 80.00 | 80.00 | S(C18)a25:0 |
| | | | CE | 39.00 | 39.00 | |
| | | | CXP | 13.00 | 13.00 | |

| Q1 Mass (Da) | Q3 Mass (Da) | Time (min) | Param | Start | Stop | ID |
|---|---|---|---|---|---|---|
| 694.000 | 264.000 | 5.23 | DP | 80.00 | 80.00 | S(C18)a26:0 |
| | | | CE | 37.00 | 37.00 | |
| | | | CXP | 13.00 | 13.00 | |

| Q1 Mass (Da) | Q3 Mass (Da) | Time (min) | Param | Start | Stop | ID |
|---|---|---|---|---|---|---|
| 708.000 | 264.000 | 5.24 | DP | 80.00 | 80.00 | S(C18)a27:0 |
| | | | CE | 49.00 | 49.00 | |
| | | | CXP | 13.00 | 13.00 | |

| Q1 Mass (Da) | Q3 Mass (Da) | Time (min) | Param | Start | Stop | ID |
|---|---|---|---|---|---|---|
| 722.000 | 264.000 | 5.25 | DP | 80.00 | 80.00 | S(C18)a28:0 |
| | | | CE | 44.00 | 44.00 | |
| | | | CXP | 13.00 | 13.00 | |

| Q1 Mass (Da) | Q3 Mass (Da) | Time (min) | Param | Start | Stop | ID |
|---|---|---|---|---|---|---|
| 680.000 | 278.000 | 5.23 | DP | 80.00 | 80.00 | S(C19)a24:0 |
| | | | CE | 45.00 | 45.00 | |
| | | | CXP | 13.00 | 13.00 | |

| Q1 Mass (Da) | Q3 Mass (Da) | Time (min) | Param | Start | Stop | ID |
|---|---|---|---|---|---|---|
| 694.000 | 278.000 | 5.29 | DP | 80.00 | 80.00 | S(C19)a25:0 |
| | | | CE | 50.00 | 50.00 | |
| | | | CXP | 13.00 | 13.00 | |

| Q1 Mass (Da) | Q3 Mass (Da) | Time (min) | Param | Start | Stop | ID |
|---|---|---|---|---|---|---|
| 708.000 | 278.000 | 5.27 | DP | 80.00 | 80.00 | S(C19)a26:0 |
| | | | CE | 50.00 | 50.00 | |
| | | | CXP | 13.00 | 13.00 | |

| Q1 Mass (Da) | Q3 Mass (Da) | Time (min) | Param | Start | Stop | ID |
|---|---|---|---|---|---|---|
| 610.000 | 292.000 | 5.14 | DP | 80.00 | 80.00 | S(C20)a18:0 |
| | | | CE | 49.00 | 49.00 | |
| | | | CXP | 13.00 | 13.00 | |

| Q1 Mass (Da) | Q3 Mass (Da) | Time (min) | Param | Start | Stop | ID |
|---|---|---|---|---|---|---|

| Q1 Mass (Da) | Q3 Mass (Da) | Time (min) | Param | Start | Stop | ID |
|---|---|---|---|---|---|---|
| 694.000 | 292.000 | 5.28 | DP | 80.00 | 80.00 | S(C20)a24:0 |
| | | | CE | 43.00 | 43.00 | |
| | | | CXP | 13.00 | 13.00 | |

| Q1 Mass (Da) | Q3 Mass (Da) | Time (min) | Param | Start | Stop | ID |
|---|---|---|---|---|---|---|
| 708.000 | 292.000 | 5.31 | DP | 80.00 | 80.00 | S(C20)a25:0 |
| | | | CE | 43.00 | 43.00 | |
| | | | CXP | 13.00 | 13.00 | |

| Q1 Mass (Da) | Q3 Mass (Da) | Time (min) | Param | Start | Stop | ID |
|---|---|---|---|---|---|---|
| 722.000 | 292.000 | 5.30 | DP | 80.00 | 80.00 | S(C20)a26:0 |
| | | | CE | 35.00 | 35.00 | |
| | | | CXP | 13.00 | 13.00 | |

| Q1 Mass (Da) | Q3 Mass (Da) | Time (min) | Param | Start | Stop | ID |
|---|---|---|---|---|---|---|
| 736.000 | 292.000 | 5.33 | DP | 80.00 | 80.00 | S(C20)a27:0 |
| | | | CE | 50.00 | 50.00 | |
| | | | CXP | 13.00 | 13.00 | |

| Q1 Mass (Da) | Q3 Mass (Da) | Time (min) | Param | Start | Stop | ID |
|---|---|---|---|---|---|---|
| 750.000 | 292.000 | 5.32 | DP | 80.00 | 80.00 | S(C20)a28:0 |
| | | | CE | 39.00 | 39.00 | |
| | | | CXP | 13.00 | 13.00 | |

| Q1 Mass (Da) | Q3 Mass (Da) | Time (min) | Param | Start | Stop | ID |
|---|---|---|---|---|---|---|
| 708.000 | 306.000 | 5.31 | DP | 80.00 | 80.00 | S(C21)a24:0 |

| | | | | | | |
|---|---|---|---|---|---|---|
| | | | CE | 41.00 | 41.00 | |
| | | | CXP | 13.00 | 13.00 | |

| Q1 Mass (Da) | Q3 Mass (Da) | Time (min) | Param | Start | Stop | ID |
|---|---|---|---|---|---|---|
| 722.000 | 306.000 | 5.34 | DP | 80.00 | 80.00 | S(C21)a25:0 |
| | | | CE | 49.00 | 49.00 | |
| | | | CXP | 13.00 | 13.00 | |

| Q1 Mass (Da) | Q3 Mass (Da) | Time (min) | Param | Start | Stop | ID |
|---|---|---|---|---|---|---|
| 736.000 | 306.000 | 5.34 | DP | 80.00 | 80.00 | S(C21)a26:0 |
| | | | CE | 37.00 | 37.00 | |
| | | | CXP | 13.00 | 13.00 | |

| Q1 Mass (Da) | Q3 Mass (Da) | Time (min) | Param | Start | Stop | ID |
|---|---|---|---|---|---|---|
| 722.000 | 320.000 | 5.35 | DP | 80.00 | 80.00 | S(C22)a24:0 |
| | | | CE | 46.00 | 46.00 | |
| | | | CXP | 13.00 | 13.00 | |

| Q1 Mass (Da) | Q3 Mass (Da) | Time (min) | Param | Start | Stop | ID |
|---|---|---|---|---|---|---|
| 736.000 | 320.000 | 5.36 | DP | 80.00 | 80.00 | S(C22)a25:0 |
| | | | CE | 44.00 | 44.00 | |
| | | | CXP | 13.00 | 13.00 | |

| Q1 Mass (Da) | Q3 Mass (Da) | Time (min) | Param | Start | Stop | ID |
|---|---|---|---|---|---|---|
| 750.000 | 320.000 | 5.39 | DP | 80.00 | 80.00 | S(C22)a26:0 |
| | | | CE | 55.00 | 55.00 | |

| | | | | | | |
|---|---|---|---|---|---|---|
| | | | CXP | 13.00 | 13.00 | |

| Q1 Mass (Da) | Q3 Mass (Da) | Time (min) | Param | Start | Stop | ID |
|---|---|---|---|---|---|---|
| 764.000 | 320.000 | 5.42 | DP | 80.00 | 80.00 | S(C22)a27:0 |
| | | | CE | 56.00 | 56.00 | |
| | | | CXP | 13.00 | 13.00 | |

| Q1 Mass (Da) | Q3 Mass (Da) | Time (min) | Param | Start | Stop | ID |
|---|---|---|---|---|---|---|
| 778.000 | 320.000 | 5.40 | DP | 80.00 | 80.00 | S(C22)a28:0 |
| | | | CE | 51.00 | 51.00 | |
| | | | CXP | 13.00 | 13.00 | |

| Q1 Mass (Da) | Q3 Mass (Da) | Time (min) | Param | Start | Stop | ID |
|---|---|---|---|---|---|---|
| 1010.000 | 280.000 | 6.55 | DP | 80.00 | 80.00 | H(C18)w30:0_18:2 |
| | | | CE | 64.00 | 64.00 | |
| | | | CXP | 13.00 | 13.00 | |

| Q1 Mass (Da) | Q3 Mass (Da) | Time (min) | Param | Start | Stop | ID |
|---|---|---|---|---|---|---|
| 1038.000 | 280.000 | 6.61 | DP | 80.00 | 80.00 | H(C18)w32:0_18:2 |
| | | | CE | 79.00 | 79.00 | |
| | | | CXP | 13.00 | 13.00 | |

| Q1 Mass (Da) | Q3 Mass (Da) | Time (min) | Param | Start | Stop | ID |
|---|---|---|---|---|---|---|
| 1066.000 | 280.000 | 6.66 | DP | 80.00 | 80.00 | H(C18)w34:0_18:2 |
| | | | CE | 94.00 | 94.00 | |
| | | | CXP | 13.00 | 13.00 | |

| Q1 Mass (Da) | Q3 Mass (Da) | Time (min) | Param | Start | Stop | ID |
|---|---|---|---|---|---|---|
| 1038.000 | 308.000 | 6.65 | DP | 80.00 | 80.00 | H(C20)w30:0_18:2 |
| | | | CE | 70.00 | 70.00 | |
| | | | CXP | 13.00 | 13.00 | |

| Q1 Mass (Da) | Q3 Mass (Da) | Time (min) | Param | Start | Stop | ID |
|---|---|---|---|---|---|---|
| 1066.000 | 308.000 | 6.70 | DP | 80.00 | 80.00 | H(C20)w32:0_18:2 |
| | | | CE | 76.00 | 76.00 | |
| | | | CXP | 13.00 | 13.00 | |

| Q1 Mass (Da) | Q3 Mass (Da) | Time (min) | Param | Start | Stop | ID |
|---|---|---|---|---|---|---|
| 1094.000 | 308.000 | 6.73 | DP | 80.00 | 80.00 | H(C20)w34:0_18:2 |
| | | | CE | 76.00 | 76.00 | |
| | | | CXP | 13.00 | 13.00 | |

| Q1 Mass (Da) | Q3 Mass (Da) | Time (min) | Param | Start | Stop | ID |
|---|---|---|---|---|---|---|
| 1080.000 | 350.000 | 6.85 | DP | 80.00 | 80.00 | H(C23)w30:0_18:2 |
| | | | CE | 90.00 | 90.00 | |
| | | | CXP | 13.00 | 13.00 | |

| Q1 Mass (Da) | Q3 Mass (Da) | Time (min) | Param | Start | Stop | ID |
|---|---|---|---|---|---|---|
| 1108.000 | 350.000 | 6.96 | DP | 80.00 | 80.00 | H(C23)w32:0_18:2 |
| | | | CE | 80.00 | 80.00 | |
| | | | CXP | 13.00 | 13.00 | |

| Q1 Mass (Da) | Q3 Mass (Da) | Time (min) | Param | Start | Stop | ID |
|---|---|---|---|---|---|---|
| 1136.000 | 350.000 | 6.98 | DP | 80.00 | 80.00 | H(C23)w34:0_18:2 |
| | | | CE | 75.00 | 75.00 | |
| | | | CXP | 13.00 | 13.00 | |

| Q1 Mass (Da) | Q3 Mass (Da) | Time (min) | Param | Start | Stop | ID |
|---|---|---|---|---|---|---|
| 1094.000 | 364.000 | 7.07 | DP | 80.00 | 80.00 | H(C24)w30:0_18:2 |
| | | | CE | 65.00 | 65.00 | |
| | | | CXP | 13.00 | 13.00 | |

| Q1 Mass (Da) | Q3 Mass (Da) | Time (min) | Param | Start | Stop | ID |
|---|---|---|---|---|---|---|
| 1122.000 | 364.000 | 6.90 | DP | 80.00 | 80.00 | H(C24)w32:0_18:2 |
| | | | CE | 89.00 | 89.00 | |
| | | | CXP | 13.00 | 13.00 | |

| Q1 Mass (Da) | Q3 Mass (Da) | Time (min) | Param | Start | Stop | ID |
|---|---|---|---|---|---|---|
| 1150.000 | 364.000 | 7.05 | DP | 80.00 | 80.00 | H(C24)w34:0_18:2 |
| | | | CE | 90.00 | 90.00 | |
| | | | CXP | 13.00 | 13.00 | |

| Q1 Mass (Da) | Q3 Mass (Da) | Time (min) | Param | Start | Stop | ID |
|---|---|---|---|---|---|---|
| 1012.700 | 264.200 | 4.86 | DP | 80.00 | 80.00 | S(C18)w30:0_18:2 |
| | | | CE | 58.00 | 58.00 | |
| | | | CXP | 15.00 | 15.00 | |

| Q1 Mass (Da) | Q3 Mass (Da) | Time (min) | Param | Start | Stop | ID |
|---|---|---|---|---|---|---|
| 1040.000 | 264.000 | 4.95 | DP | 80.00 | 80.00 | S(C18)w32:0_18:2 |
| | | | CE | 70.00 | 70.00 | |
| | | | CXP | 15.00 | 15.00 | |

| Q1 Mass (Da) | Q3 Mass (Da) | Time (min) | Param | Start | Stop | ID |
|---|---|---|---|---|---|---|
| 1068.000 | 264.000 | 4.99 | DP | 80.00 | 80.00 | S(C18)w34:0_18:2 |
| | | | CE | 100.00 | 100.00 | |
| | | | CXP | 15.00 | 15.00 | |

| Q1 Mass (Da) | Q3 Mass (Da) | Time (min) | Param | Start | Stop | ID |
|---|---|---|---|---|---|---|
| 1012.000 | 292.000 | 4.93 | DP | 80.00 | 80.00 | S(C20)w28:0_18:2 |
| | | | CE | 64.00 | 64.00 | |
| | | | CXP | 15.00 | 15.00 | |

| Q1 Mass (Da) | Q3 Mass (Da) | Time (min) | Param | Start | Stop | ID |
|---|---|---|---|---|---|---|
| 1026.000 | 292.000 | 4.97 | DP | 80.00 | 80.00 | S(C20)w29:0_18:2 |
| | | | CE | 105.00 | 105.00 | |
| | | | CXP | 15.00 | 15.00 | |

| Q1 Mass (Da) | Q3 Mass (Da) | Time (min) | Param | Start | Stop | ID |
|---|---|---|---|---|---|---|
| 1040.000 | 292.000 | 4.99 | DP | 80.00 | 80.00 | S(C20)w30:0_18:2 |
| | | | CE | 54.00 | 54.00 | |
| | | | CXP | 15.00 | 15.00 | |

| Q1 Mass (Da) | Q3 Mass (Da) | Time (min) | Param | Start | Stop | ID |
|---|---|---|---|---|---|---|
| 1054.000 | 292.000 | 5.01 | DP | 80.00 | 80.00 | S(C20)w31:0_18:2 |
| | | | CE | 63.00 | 63.00 | |
| | | | CXP | 15.00 | 15.00 | |

| Q1 Mass (Da) | Q3 Mass (Da) | Time (min) | Param | Start | Stop | ID |
|---|---|---|---|---|---|---|
| 1068.000 | 292.000 | 5.05 | DP | 80.00 | 80.00 | S(C20)w32:0_18:2 |
| | | | CE | 69.00 | 69.00 | |
| | | | CXP | 15.00 | 15.00 | |

| Q1 Mass (Da) | Q3 Mass (Da) | Time (min) | Param | Start | Stop | ID |
|---|---|---|---|---|---|---|
| 1082.000 | 292.000 | 4.99 | DP | 80.00 | 80.00 | S(C20)w33:0_18:2 |
| | | | CE | 96.00 | 96.00 | |
| | | | CXP | 15.00 | 15.00 | |

| Q1 Mass (Da) | Q3 Mass (Da) | Time (min) | Param | Start | Stop | ID |
|---|---|---|---|---|---|---|
| 1096.000 | 292.000 | 5.12 | DP | 80.00 | 80.00 | S(C20)w34:0_18:2 |
| | | | CE | 70.00 | 70.00 | |
| | | | CXP | 15.00 | 15.00 | |

| Q1 Mass (Da) | Q3 Mass (Da) | Time (min) | Param | Start | Stop | ID |
|---|---|---|---|---|---|---|
| 1054.000 | 306.000 | 5.04 | DP | 80.00 | 80.00 | S(C21)w30:0_18:2 |
| | | | CE | 66.00 | 66.00 | |
| | | | CXP | 15.00 | 15.00 | |

| Q1 Mass (Da) | Q3 Mass (Da) | Time (min) | Param | Start | Stop | ID |
|---|---|---|---|---|---|---|

| Q1 Mass (Da) | Q3 Mass (Da) | Time (min) | Param | Start | Stop | ID |
|---|---|---|---|---|---|---|
| 1082.000 | 306.000 | 5.08 | DP | 80.00 | 80.00 | S(C21)w32:0_18:2 |
| | | | CE | 63.00 | 63.00 | |
| | | | CXP | 15.00 | 15.00 | |

| Q1 Mass (Da) | Q3 Mass (Da) | Time (min) | Param | Start | Stop | ID |
|---|---|---|---|---|---|---|
| 1110.000 | 306.000 | 5.15 | DP | 80.00 | 80.00 | S(C21)w34:0_18:2 |
| | | | CE | 85.00 | 85.00 | |
| | | | CXP | 15.00 | 15.00 | |

| Q1 Mass (Da) | Q3 Mass (Da) | Time (min) | Param | Start | Stop | ID |
|---|---|---|---|---|---|---|
| 1068.000 | 320.000 | 5.08 | DP | 80.00 | 80.00 | S(C22)w30:0_18:2 |
| | | | CE | 74.00 | 74.00 | |
| | | | CXP | 15.00 | 15.00 | |

| Q1 Mass (Da) | Q3 Mass (Da) | Time (min) | Param | Start | Stop | ID |
|---|---|---|---|---|---|---|
| 1082.000 | 320.000 | 5.13 | DP | 80.00 | 80.00 | S(C22)w31:0_18:2 |
| | | | CE | 76.00 | 76.00 | |
| | | | CXP | 15.00 | 15.00 | |

| Q1 Mass (Da) | Q3 Mass (Da) | Time (min) | Param | Start | Stop | ID |
|---|---|---|---|---|---|---|
| 1096.000 | 320.000 | 5.12 | DP | 80.00 | 80.00 | S(C22)w32:0_18:2 |
| | | | CE | 80.00 | 80.00 | |
| | | | CXP | 15.00 | 15.00 | |

| Q1 Mass (Da) | Q3 Mass (Da) | Time (min) | Param | Start | Stop | ID |
|---|---|---|---|---|---|---|
| 1110.000 | 320.000 | 5.10 | DP | 80.00 | 80.00 | S(C22)w33:0_18:2 |

| | | | Param | Start | Stop | ID |
|---|---|---|---|---|---|---|
| | | | CE | 61.00 | 61.00 | |
| | | | CXP | 15.00 | 15.00 | |

| Q1 Mass (Da) | Q3 Mass (Da) | Time (min) | Param | Start | Stop | ID |
|---|---|---|---|---|---|---|
| 1124.000 | 320.000 | 5.18 | DP | 80.00 | 80.00 | S(C22)w34:0_18:2 |
| | | | CE | 80.00 | 80.00 | |
| | | | CXP | 15.00 | 15.00 | |

| Q1 Mass (Da) | Q3 Mass (Da) | Time (min) | Param | Start | Stop | ID |
|---|---|---|---|---|---|---|
| 1096.000 | 348.000 | 5.12 | DP | 80.00 | 80.00 | S(C24)w30:0_18:2 |
| | | | CE | 94.00 | 94.00 | |
| | | | CXP | 15.00 | 15.00 | |

| Q1 Mass (Da) | Q3 Mass (Da) | Time (min) | Param | Start | Stop | ID |
|---|---|---|---|---|---|---|
| 1124.000 | 348.000 | 5.19 | DP | 80.00 | 80.00 | S(C24)w32:0_18:2 |
| | | | CE | 70.00 | 70.00 | |
| | | | CXP | 15.00 | 15.00 | |

| Q1 Mass (Da) | Q3 Mass (Da) | Time (min) | Param | Start | Stop | ID |
|---|---|---|---|---|---|---|
| 1152.000 | 348.000 | 5.24 | DP | 80.00 | 80.00 | S(C24)w34:0_18:2 |
| | | | CE | 100.00 | 100.00 | |
| | | | CXP | 15.00 | 15.00 | |

| Q1 Mass (Da) | Q3 Mass (Da) | Time (min) | Param | Start | Stop | ID |
|---|---|---|---|---|---|---|
| 624.000 | 256.000 | 3.85 | DP | 80.00 | 80.00 | DS(C16)24:0 |
| | | | CE | 45.00 | 45.00 | |

| | | | | | | |
|---|---|---|---|---|---|---|
| | | | CXP | 13.00 | 13.00 | |

| Q1 Mass (Da) | Q3 Mass (Da) | Time (min) | Param | Start | Stop | ID |
|---|---|---|---|---|---|---|
| 638.000 | 256.000 | 3.87 | DP | 80.00 | 80.00 | DS(C16)25:0 |
| | | | CE | 53.00 | 53.00 | |
| | | | CXP | 13.00 | 13.00 | |

| Q1 Mass (Da) | Q3 Mass (Da) | Time (min) | Param | Start | Stop | ID |
|---|---|---|---|---|---|---|
| 652.000 | 256.000 | 3.99 | DP | 80.00 | 80.00 | DS(C16)26:0 |
| | | | CE | 21.00 | 21.00 | |
| | | | CXP | 13.00 | 13.00 | |

| Q1 Mass (Da) | Q3 Mass (Da) | Time (min) | Param | Start | Stop | ID |
|---|---|---|---|---|---|---|
| 638.000 | 270.000 | 3.86 | DP | 80.00 | 80.00 | DS(C17)24:0 |
| | | | CE | 39.00 | 39.00 | |
| | | | CXP | 13.00 | 13.00 | |

| Q1 Mass (Da) | Q3 Mass (Da) | Time (min) | Param | Start | Stop | ID |
|---|---|---|---|---|---|---|
| 652.000 | 270.000 | 3.89 | DP | 80.00 | 80.00 | DS(C17)25:0 |
| | | | CE | 55.00 | 55.00 | |
| | | | CXP | 13.00 | 13.00 | |

| Q1 Mass (Da) | Q3 Mass (Da) | Time (min) | Param | Start | Stop | ID |
|---|---|---|---|---|---|---|
| 666.000 | 270.000 | 3.89 | DP | 80.00 | 80.00 | DS(C17)26:0 |
| | | | CE | 31.00 | 31.00 | |
| | | | CXP | 13.00 | 13.00 | |

| Q1 Mass (Da) | Q3 Mass (Da) | Time (min) | Param | Start | Stop | ID |
|---|---|---|---|---|---|---|
| 568.300 | 266.200 | 3.76 | DP | 80.00 | 80.00 | DS(C18)18:0 |
| | | | CE | 35.00 | 35.00 | |
| | | | CXP | 13.00 | 13.00 | |

| Q1 Mass (Da) | Q3 Mass (Da) | Time (min) | Param | Start | Stop | ID |
|---|---|---|---|---|---|---|
| 596.300 | 266.200 | 3.77 | DP | 80.00 | 80.00 | DS(C18)20:0 |
| | | | CE | 48.00 | 48.00 | |
| | | | CXP | 13.00 | 13.00 | |

| Q1 Mass (Da) | Q3 Mass (Da) | Time (min) | Param | Start | Stop | ID |
|---|---|---|---|---|---|---|
| 638.000 | 284.000 | 3.89 | DP | 80.00 | 80.00 | DS(C18)23:0 |
| | | | CE | 45.00 | 45.00 | |
| | | | CXP | 13.00 | 13.00 | |

| Q1 Mass (Da) | Q3 Mass (Da) | Time (min) | Param | Start | Stop | ID |
|---|---|---|---|---|---|---|
| 652.300 | 266.200 | 3.90 | DP | 80.00 | 80.00 | DS(C18)24:0 |
| | | | CE | 45.00 | 45.00 | |
| | | | CXP | 13.00 | 13.00 | |

| Q1 Mass (Da) | Q3 Mass (Da) | Time (min) | Param | Start | Stop | ID |
|---|---|---|---|---|---|---|
| 666.000 | 284.000 | 3.92 | DP | 80.00 | 80.00 | DS(C18)25:0 |
| | | | CE | 39.00 | 39.00 | |
| | | | CXP | 13.00 | 13.00 | |

| Q1 Mass (Da) | Q3 Mass (Da) | Time (min) | Param | Start | Stop | ID |
|---|---|---|---|---|---|---|
| 680.000 | 284.000 | 3.94 | DP | 80.00 | 80.00 | DS(C18)26:0 |
| | | | CE | 35.00 | 35.00 | |
| | | | CXP | 13.00 | 13.00 | |

| Q1 Mass (Da) | Q3 Mass (Da) | Time (min) | Param | Start | Stop | ID |
|---|---|---|---|---|---|---|
| 708.000 | 284.000 | 3.96 | DP | 80.00 | 80.00 | DS(C18)28:0 |
| | | | CE | 53.00 | 53.00 | |
| | | | CXP | 13.00 | 13.00 | |

| Q1 Mass (Da) | Q3 Mass (Da) | Time (min) | Param | Start | Stop | ID |
|---|---|---|---|---|---|---|
| 666.000 | 298.000 | 3.93 | DP | 80.00 | 80.00 | DS(C19)24:0 |
| | | | CE | 36.00 | 36.00 | |
| | | | CXP | 13.00 | 13.00 | |

| Q1 Mass (Da) | Q3 Mass (Da) | Time (min) | Param | Start | Stop | ID |
|---|---|---|---|---|---|---|
| 680.000 | 298.000 | 4.00 | DP | 80.00 | 80.00 | DS(C19)25:0 |
| | | | CE | 60.00 | 60.00 | |
| | | | CXP | 13.00 | 13.00 | |

| Q1 Mass (Da) | Q3 Mass (Da) | Time (min) | Param | Start | Stop | ID |
|---|---|---|---|---|---|---|
| 694.000 | 298.000 | 3.93 | DP | 80.00 | 80.00 | DS(C19)26:0 |
| | | | CE | 35.00 | 35.00 | |
| | | | CXP | 13.00 | 13.00 | |

| Q1 Mass (Da) | Q3 Mass (Da) | Time (min) | Param | Start | Stop | ID |
|---|---|---|---|---|---|---|
| 624.000 | 312.000 | 3.88 | DP | 80.00 | 80.00 | DS(C20)20:0 |
| | | | CE | 40.00 | 40.00 | |
| | | | CXP | 13.00 | 13.00 | |

| Q1 Mass (Da) | Q3 Mass (Da) | Time (min) | Param | Start | Stop | ID |
|---|---|---|---|---|---|---|
| 652.000 | 312.000 | 3.94 | DP | 80.00 | 80.00 | DS(C20)22:0 |
| | | | CE | 41.00 | 41.00 | |
| | | | CXP | 13.00 | 13.00 | |

| Q1 Mass (Da) | Q3 Mass (Da) | Time (min) | Param | Start | Stop | ID |
|---|---|---|---|---|---|---|
| 666.000 | 312.000 | 3.95 | DP | 80.00 | 80.00 | DS(C20)23:0 |
| | | | CE | 41.00 | 41.00 | |
| | | | CXP | 13.00 | 13.00 | |

| Q1 Mass (Da) | Q3 Mass (Da) | Time (min) | Param | Start | Stop | ID |
|---|---|---|---|---|---|---|
| 680.000 | 312.000 | 3.95 | DP | 80.00 | 80.00 | DS(C20)24:0 |
| | | | CE | 49.00 | 49.00 | |
| | | | CXP | 13.00 | 13.00 | |

| Q1 Mass (Da) | Q3 Mass (Da) | Time (min) | Param | Start | Stop | ID |
|---|---|---|---|---|---|---|
| 694.000 | 312.000 | 3.97 | DP | 80.00 | 80.00 | DS(C20)25:0 |
| | | | CE | 51.00 | 51.00 | |
| | | | CXP | 13.00 | 13.00 | |

| Q1 Mass (Da) | Q3 Mass (Da) | Time (min) | Param | Start | Stop | ID |
|---|---|---|---|---|---|---|
| 708.000 | 312.000 | 3.99 | DP | 80.00 | 80.00 | DS(C20)26:0 |
| | | | CE | 45.00 | 45.00 | |
| | | | CXP | 13.00 | 13.00 | |

| Q1 Mass (Da) | Q3 Mass (Da) | Time (min) | Param | Start | Stop | ID |
|---|---|---|---|---|---|---|
| 736.000 | 312.000 | 4.03 | DP | 80.00 | 80.00 | DS(C20)28:0 |
| | | | CE | 52.00 | 52.00 | |
| | | | CXP | 13.00 | 13.00 | |

| Q1 Mass (Da) | Q3 Mass (Da) | Time (min) | Param | Start | Stop | ID |
|---|---|---|---|---|---|---|
| 694.000 | 326.000 | 3.98 | DP | 80.00 | 80.00 | DS(C21)24:0 |
| | | | CE | 35.00 | 35.00 | |
| | | | CXP | 13.00 | 13.00 | |

| Q1 Mass (Da) | Q3 Mass (Da) | Time (min) | Param | Start | Stop | ID |
|---|---|---|---|---|---|---|
| 708.000 | 326.000 | 4.03 | DP | 80.00 | 80.00 | DS(C21)25:0 |
| | | | CE | 55.00 | 55.00 | |
| | | | CXP | 13.00 | 13.00 | |

| Q1 Mass (Da) | Q3 Mass (Da) | Time (min) | Param | Start | Stop | ID |
|---|---|---|---|---|---|---|
| 722.000 | 326.000 | 4.05 | DP | 80.00 | 80.00 | DS(C21)26:0 |
| | | | CE | 34.00 | 34.00 | |
| | | | CXP | 13.00 | 13.00 | |

| Q1 Mass (Da) | Q3 Mass (Da) | Time (min) | Param | Start | Stop | ID |
|---|---|---|---|---|---|---|

| | | | | | | |
|---|---|---|---|---|---|---|
| 624.000 | 340.000 | 3.87 | DP | 80.00 | 80.00 | DS(C22)18:0 |
| | | | CE | 36.00 | 36.00 | |
| | | | CXP | 13.00 | 13.00 | |
| Q1 Mass (Da) | Q3 Mass (Da) | Time (min) | Param | Start | Stop | ID |
| 652.000 | 340.000 | 3.93 | DP | 80.00 | 80.00 | DS(C22)20:0 |
| | | | CE | 38.00 | 38.00 | |
| | | | CXP | 13.00 | 13.00 | |
| Q1 Mass (Da) | Q3 Mass (Da) | Time (min) | Param | Start | Stop | ID |
| 680.000 | 340.000 | 3.97 | DP | 80.00 | 80.00 | DS(C22)22:0 |
| | | | CE | 48.00 | 48.00 | |
| | | | CXP | 13.00 | 13.00 | |
| Q1 Mass (Da) | Q3 Mass (Da) | Time (min) | Param | Start | Stop | ID |
| 708.000 | 340.000 | 4.02 | DP | 80.00 | 80.00 | DS(C22)24:0 |
| | | | CE | 40.00 | 40.00 | |
| | | | CXP | 13.00 | 13.00 | |
| Q1 Mass (Da) | Q3 Mass (Da) | Time (min) | Param | Start | Stop | ID |
| 722.000 | 340.000 | 4.07 | DP | 80.00 | 80.00 | DS(C22)25:0 |
| | | | CE | 49.00 | 49.00 | |
| | | | CXP | 13.00 | 13.00 | |
| Q1 Mass (Da) | Q3 Mass (Da) | Time (min) | Param | Start | Stop | ID |
| 736.000 | 340.000 | 4.06 | DP | 80.00 | 80.00 | DS(C22)26:0 |

| Q1 Mass (Da) | Q3 Mass (Da) | Time (min) | Param | Start | Stop | ID |
|---|---|---|---|---|---|---|
| | | | CE | 44.00 | 44.00 | |
| | | | CXP | 13.00 | 13.00 | |
| Q1 Mass (Da) | Q3 Mass (Da) | Time (min) | Param | Start | Stop | ID |
| 764.000 | 340.000 | 4.16 | DP | 80.00 | 80.00 | DS(C22)28:0 |
| | | | CE | 40.00 | 40.00 | |
| | | | CXP | 13.00 | 13.00 | |
| Q1 Mass (Da) | Q3 Mass (Da) | Time (min) | Param | Start | Stop | ID |
| 638.000 | 354.000 | 3.90 | DP | 80.00 | 80.00 | DS(C23)18:0 |
| | | | CE | 40.00 | 40.00 | |
| | | | CXP | 13.00 | 13.00 | |
| Q1 Mass (Da) | Q3 Mass (Da) | Time (min) | Param | Start | Stop | ID |
| 666.000 | 354.000 | 3.95 | DP | 80.00 | 80.00 | DS(C23)20:0 |
| | | | CE | 44.00 | 44.00 | |
| | | | CXP | 13.00 | 13.00 | |
| Q1 Mass (Da) | Q3 Mass (Da) | Time (min) | Param | Start | Stop | ID |
| 722.000 | 354.000 | 4.02 | DP | 80.00 | 80.00 | DS(C23)24:0 |
| | | | CE | 50.00 | 50.00 | |
| | | | CXP | 13.00 | 13.00 | |
| Q1 Mass (Da) | Q3 Mass (Da) | Time (min) | Param | Start | Stop | ID |
| 736.000 | 354.000 | 4.13 | DP | 80.00 | 80.00 | DS(C23)25:0 |
| | | | CE | 45.00 | 45.00 | |

| | | | | | | |
|---|---|---|---|---|---|---|
| | | | CXP | 13.00 | 13.00 | |

| Q1 Mass (Da) | Q3 Mass (Da) | Time (min) | Param | Start | Stop | ID |
|---|---|---|---|---|---|---|
| 750.000 | 354.000 | 4.17 | DP | 80.00 | 80.00 | DS(C23)26:0 |
| | | | CE | 40.00 | 40.00 | |
| | | | CXP | 13.00 | 13.00 | |

| Q1 Mass (Da) | Q3 Mass (Da) | Time (min) | Param | Start | Stop | ID |
|---|---|---|---|---|---|---|
| 624.000 | 368.000 | 3.87 | DP | 80.00 | 80.00 | DS(C24)16:0 |
| | | | CE | 36.00 | 36.00 | |
| | | | CXP | 13.00 | 13.00 | |

| Q1 Mass (Da) | Q3 Mass (Da) | Time (min) | Param | Start | Stop | ID |
|---|---|---|---|---|---|---|
| 638.000 | 368.000 | 3.86 | DP | 80.00 | 80.00 | DS(C24)17:0 |
| | | | CE | 44.00 | 44.00 | |
| | | | CXP | 13.00 | 13.00 | |

| Q1 Mass (Da) | Q3 Mass (Da) | Time (min) | Param | Start | Stop | ID |
|---|---|---|---|---|---|---|
| 652.000 | 368.000 | 3.90 | DP | 80.00 | 80.00 | DS(C24)18:0 |
| | | | CE | 47.00 | 47.00 | |
| | | | CXP | 13.00 | 13.00 | |

| Q1 Mass (Da) | Q3 Mass (Da) | Time (min) | Param | Start | Stop | ID |
|---|---|---|---|---|---|---|
| 666.000 | 368.000 | 3.92 | DP | 80.00 | 80.00 | DS(C24)19:0 |
| | | | CE | 50.00 | 50.00 | |
| | | | CXP | 13.00 | 13.00 | |

| Q1 Mass (Da) | Q3 Mass (Da) | Time (min) | Param | Start | Stop | ID |
|---|---|---|---|---|---|---|
| 680.000 | 368.000 | 3.97 | DP | 80.00 | 80.00 | DS(C24)20:0 |
| | | | CE | 48.00 | 48.00 | |
| | | | CXP | 13.00 | 13.00 | |

| Q1 Mass (Da) | Q3 Mass (Da) | Time (min) | Param | Start | Stop | ID |
|---|---|---|---|---|---|---|
| 694.000 | 368.000 | 3.97 | DP | 80.00 | 80.00 | DS(C24)21:0 |
| | | | CE | 40.00 | 40.00 | |
| | | | CXP | 13.00 | 13.00 | |

| Q1 Mass (Da) | Q3 Mass (Da) | Time (min) | Param | Start | Stop | ID |
|---|---|---|---|---|---|---|
| 708.000 | 368.000 | 4.04 | DP | 80.00 | 80.00 | DS(C24)22:0 |
| | | | CE | 46.00 | 46.00 | |
| | | | CXP | 13.00 | 13.00 | |

| Q1 Mass (Da) | Q3 Mass (Da) | Time (min) | Param | Start | Stop | ID |
|---|---|---|---|---|---|---|
| 736.000 | 368.000 | 4.08 | DP | 80.00 | 80.00 | DS(C24)24:0 |
| | | | CE | 44.00 | 44.00 | |
| | | | CXP | 13.00 | 13.00 | |

| Q1 Mass (Da) | Q3 Mass (Da) | Time (min) | Param | Start | Stop | ID |
|---|---|---|---|---|---|---|
| 750.000 | 368.000 | 4.11 | DP | 80.00 | 80.00 | DS(C24)25:0 |
| | | | CE | 36.00 | 36.00 | |
| | | | CXP | 13.00 | 13.00 | |

| Q1 Mass (Da) | Q3 Mass (Da) | Time (min) | Param | Start | Stop | ID |
|---|---|---|---|---|---|---|
| 764.000 | 368.000 | 4.16 | DP | 80.00 | 80.00 | DS(C24)26:0 |
| | | | CE | 60.00 | 60.00 | |
| | | | CXP | 13.00 | 13.00 | |

| Q1 Mass (Da) | Q3 Mass (Da) | Time (min) | Param | Start | Stop | ID |
|---|---|---|---|---|---|---|
| 652.000 | 396.000 | 3.88 | DP | 80.00 | 80.00 | DS(C26)16:0 |
| | | | CE | 45.00 | 45.00 | |
| | | | CXP | 13.00 | 13.00 | |

| Q1 Mass (Da) | Q3 Mass (Da) | Time (min) | Param | Start | Stop | ID |
|---|---|---|---|---|---|---|
| 708.000 | 396.000 | 4.01 | DP | 80.00 | 80.00 | DS(C26)20:0 |
| | | | CE | 49.00 | 49.00 | |
| | | | CXP | 13.00 | 13.00 | |

| Q1 Mass (Da) | Q3 Mass (Da) | Time (min) | Param | Start | Stop | ID |
|---|---|---|---|---|---|---|
| 736.000 | 396.000 | 4.08 | DP | 80.00 | 80.00 | DS(C26)22:0 |
| | | | CE | 50.00 | 50.00 | |
| | | | CXP | 13.00 | 13.00 | |

| Q1 Mass (Da) | Q3 Mass (Da) | Time (min) | Param | Start | Stop | ID |
|---|---|---|---|---|---|---|
| 764.000 | 396.000 | 4.16 | DP | 80.00 | 80.00 | DS(C26)24:0 |
| | | | CE | 52.00 | 52.00 | |
| | | | CXP | 13.00 | 13.00 | |

| Q1 Mass (Da) | Q3 Mass (Da) | Time (min) | Param | Start | Stop | ID |
|---|---|---|---|---|---|---|
| 778.000 | 396.000 | 4.13 | DP | 80.00 | 80.00 | DS(C26)25:0 |
| | | | CE | 60.00 | 60.00 | |
| | | | CXP | 13.00 | 13.00 | |

| Q1 Mass (Da) | Q3 Mass (Da) | Time (min) | Param | Start | Stop | ID |
|---|---|---|---|---|---|---|
| 792.000 | 396.000 | 4.23 | DP | 80.00 | 80.00 | DS(C26)26:0 |
| | | | CE | 55.00 | 55.00 | |
| | | | CXP | 13.00 | 13.00 | |

| Q1 Mass (Da) | Q3 Mass (Da) | Time (min) | Param | Start | Stop | ID |
|---|---|---|---|---|---|---|
| 620.000 | 252.000 | 5.67 | DP | 80.00 | 80.00 | H(C16)24:0 |
| | | | CE | 25.00 | 25.00 | |
| | | | CXP | 13.00 | 13.00 | |

| Q1 Mass (Da) | Q3 Mass (Da) | Time (min) | Param | Start | Stop | ID |
|---|---|---|---|---|---|---|
| 634.000 | 252.000 | 5.69 | DP | 80.00 | 80.00 | H(C16)25:0 |
| | | | CE | 31.00 | 31.00 | |
| | | | CXP | 13.00 | 13.00 | |

| Q1 Mass (Da) | Q3 Mass (Da) | Time (min) | Param | Start | Stop | ID |
|---|---|---|---|---|---|---|
| 648.000 | 252.000 | 5.71 | DP | 80.00 | 80.00 | H(C16)26:0 |
| | | | CE | 31.00 | 31.00 | |
| | | | CXP | 13.00 | 13.00 | |

| Q1 Mass (Da) | Q3 Mass (Da) | Time (min) | Param | Start | Stop | ID |
|---|---|---|---|---|---|---|
| 662.000 | 252.000 | 5.75 | DP | 80.00 | 80.00 | H(C16)27:0 |
| | | | CE | 45.00 | 45.00 | |
| | | | CXP | 13.00 | 13.00 | |

| Q1 Mass (Da) | Q3 Mass (Da) | Time (min) | Param | Start | Stop | ID |
|---|---|---|---|---|---|---|
| 676.000 | 252.000 | 5.76 | DP | 80.00 | 80.00 | H(C16)28:0 |
| | | | CE | 45.00 | 45.00 | |
| | | | CXP | 13.00 | 13.00 | |

| Q1 Mass (Da) | Q3 Mass (Da) | Time (min) | Param | Start | Stop | ID |
|---|---|---|---|---|---|---|
| 634.000 | 266.000 | 5.72 | DP | 80.00 | 80.00 | H(C17)24:0 |
| | | | CE | 34.00 | 34.00 | |
| | | | CXP | 13.00 | 13.00 | |

| Q1 Mass (Da) | Q3 Mass (Da) | Time (min) | Param | Start | Stop | ID |
|---|---|---|---|---|---|---|
| 648.000 | 266.000 | 5.72 | DP | 80.00 | 80.00 | H(C17)25:0 |
| | | | CE | 33.00 | 33.00 | |
| | | | CXP | 13.00 | 13.00 | |

| Q1 Mass (Da) | Q3 Mass (Da) | Time (min) | Param | Start | Stop | ID |
|---|---|---|---|---|---|---|
| 662.000 | 266.000 | 5.74 | DP | 80.00 | 80.00 | H(C17)26:0 |
| | | | CE | 31.00 | 31.00 | |
| | | | CXP | 13.00 | 13.00 | |

| Q1 Mass (Da) | Q3 Mass (Da) | Time (min) | Param | Start | Stop | ID |
|---|---|---|---|---|---|---|

| | | | | | | |
|---|---|---|---|---|---|---|
| 676.000 | 266.000 | 5.75 | DP | 80.00 | 80.00 | H(C17)27:0 |
| | | | CE | 32.00 | 32.00 | |
| | | | CXP | 13.00 | 13.00 | |

| Q1 Mass (Da) | Q3 Mass (Da) | Time (min) | Param | Start | Stop | ID |
|---|---|---|---|---|---|---|
| 690.000 | 266.000 | 5.76 | DP | 80.00 | 80.00 | H(C17)28:0 |
| | | | CE | 34.00 | 34.00 | |
| | | | CXP | 13.00 | 13.00 | |

| Q1 Mass (Da) | Q3 Mass (Da) | Time (min) | Param | Start | Stop | ID |
|---|---|---|---|---|---|---|
| 634.000 | 280.000 | 5.73 | DP | 80.00 | 80.00 | H(C18)23:0 |
| | | | CE | 25.00 | 25.00 | |
| | | | CXP | 13.00 | 13.00 | |

| Q1 Mass (Da) | Q3 Mass (Da) | Time (min) | Param | Start | Stop | ID |
|---|---|---|---|---|---|---|
| 648.000 | 280.000 | 5.75 | DP | 80.00 | 80.00 | H(C18)24:0 |
| | | | CE | 32.00 | 32.00 | |
| | | | CXP | 13.00 | 13.00 | |

| Q1 Mass (Da) | Q3 Mass (Da) | Time (min) | Param | Start | Stop | ID |
|---|---|---|---|---|---|---|
| 662.000 | 280.000 | 5.76 | DP | 80.00 | 80.00 | H(C18)25:0 |
| | | | CE | 40.00 | 40.00 | |
| | | | CXP | 13.00 | 13.00 | |

| Q1 Mass (Da) | Q3 Mass (Da) | Time (min) | Param | Start | Stop | ID |
|---|---|---|---|---|---|---|
| 676.000 | 280.000 | 5.78 | DP | 80.00 | 80.00 | H(C18)26:0 |

| | | | | | | |
|---|---|---|---|---|---|---|
| | | | CE | 38.00 | 38.00 | |
| | | | CXP | 13.00 | 13.00 | |

| Q1 Mass (Da) | Q3 Mass (Da) | Time (min) | Param | Start | Stop | ID |
|---|---|---|---|---|---|---|
| 690.000 | 280.000 | 5.78 | DP | 80.00 | 80.00 | H(C18)27:0 |
| | | | CE | 38.00 | 38.00 | |
| | | | CXP | 13.00 | 13.00 | |

| Q1 Mass (Da) | Q3 Mass (Da) | Time (min) | Param | Start | Stop | ID |
|---|---|---|---|---|---|---|
| 704.000 | 280.000 | 5.80 | DP | 80.00 | 80.00 | H(C18)28:0 |
| | | | CE | 32.00 | 32.00 | |
| | | | CXP | 13.00 | 13.00 | |

| Q1 Mass (Da) | Q3 Mass (Da) | Time (min) | Param | Start | Stop | ID |
|---|---|---|---|---|---|---|
| 718.000 | 280.000 | 5.81 | DP | 80.00 | 80.00 | H(C18)29:0 |
| | | | CE | 40.00 | 40.00 | |
| | | | CXP | 13.00 | 13.00 | |

| Q1 Mass (Da) | Q3 Mass (Da) | Time (min) | Param | Start | Stop | ID |
|---|---|---|---|---|---|---|
| 732.000 | 280.000 | 5.84 | DP | 80.00 | 80.00 | H(C18)30:0 |
| | | | CE | 35.00 | 35.00 | |
| | | | CXP | 13.00 | 13.00 | |

| Q1 Mass (Da) | Q3 Mass (Da) | Time (min) | Param | Start | Stop | ID |
|---|---|---|---|---|---|---|
| 634.000 | 294.000 | 5.75 | DP | 80.00 | 80.00 | H(C19)22:0 |
| | | | CE | 36.00 | 36.00 | |

| Q1 Mass (Da) | Q3 Mass (Da) | Time (min) | Param | Start | Stop | ID |
|---|---|---|---|---|---|---|
| | | | CXP | 13.00 | 13.00 | |

| Q1 Mass (Da) | Q3 Mass (Da) | Time (min) | Param | Start | Stop | ID |
|---|---|---|---|---|---|---|
| 648.000 | 294.000 | 5.77 | DP | 80.00 | 80.00 | H(C19)23:0 |
| | | | CE | 31.00 | 31.00 | |
| | | | CXP | 13.00 | 13.00 | |

| Q1 Mass (Da) | Q3 Mass (Da) | Time (min) | Param | Start | Stop | ID |
|---|---|---|---|---|---|---|
| 662.000 | 294.000 | 5.78 | DP | 80.00 | 80.00 | H(C19)24:0 |
| | | | CE | 40.00 | 40.00 | |
| | | | CXP | 13.00 | 13.00 | |

| Q1 Mass (Da) | Q3 Mass (Da) | Time (min) | Param | Start | Stop | ID |
|---|---|---|---|---|---|---|
| 676.000 | 294.000 | 5.80 | DP | 80.00 | 80.00 | H(C19)25:0 |
| | | | CE | 30.00 | 30.00 | |
| | | | CXP | 13.00 | 13.00 | |

| Q1 Mass (Da) | Q3 Mass (Da) | Time (min) | Param | Start | Stop | ID |
|---|---|---|---|---|---|---|
| 690.000 | 294.000 | 5.81 | DP | 80.00 | 80.00 | H(C19)26:0 |
| | | | CE | 34.00 | 34.00 | |
| | | | CXP | 13.00 | 13.00 | |

| Q1 Mass (Da) | Q3 Mass (Da) | Time (min) | Param | Start | Stop | ID |
|---|---|---|---|---|---|---|
| 704.000 | 294.000 | 5.83 | DP | 80.00 | 80.00 | H(C19)27:0 |
| | | | CE | 49.00 | 49.00 | |
| | | | CXP | 13.00 | 13.00 | |

| Q1 Mass (Da) | Q3 Mass (Da) | Time (min) | Param | Start | Stop | ID |
|---|---|---|---|---|---|---|
| 718.000 | 294.000 | 5.83 | DP | 80.00 | 80.00 | H(C19)28:0 |
| | | | CE | 39.00 | 39.00 | |
| | | | CXP | 13.00 | 13.00 | |

| Q1 Mass (Da) | Q3 Mass (Da) | Time (min) | Param | Start | Stop | ID |
|---|---|---|---|---|---|---|
| 564.000 | 308.000 | 5.67 | DP | 80.00 | 80.00 | H(C20)16:0 |
| | | | CE | 30.00 | 30.00 | |
| | | | CXP | 13.00 | 13.00 | |

| Q1 Mass (Da) | Q3 Mass (Da) | Time (min) | Param | Start | Stop | ID |
|---|---|---|---|---|---|---|
| 592.000 | 308.000 | 5.71 | DP | 80.00 | 80.00 | H(C20)18:0 |
| | | | CE | 32.00 | 32.00 | |
| | | | CXP | 13.00 | 13.00 | |

| Q1 Mass (Da) | Q3 Mass (Da) | Time (min) | Param | Start | Stop | ID |
|---|---|---|---|---|---|---|
| 648.000 | 308.000 | 5.79 | DP | 80.00 | 80.00 | H(C20)22:0 |
| | | | CE | 35.00 | 35.00 | |
| | | | CXP | 13.00 | 13.00 | |

| Q1 Mass (Da) | Q3 Mass (Da) | Time (min) | Param | Start | Stop | ID |
|---|---|---|---|---|---|---|
| 676.000 | 308.000 | 5.81 | DP | 80.00 | 80.00 | H(C20)24:0 |
| | | | CE | 33.00 | 33.00 | |
| | | | CXP | 13.00 | 13.00 | |

| Q1 Mass (Da) | Q3 Mass (Da) | Time (min) | Param | Start | Stop | ID |
|---|---|---|---|---|---|---|
| 690.000 | 308.000 | 5.83 | DP | 80.00 | 80.00 | H(C20)25:0 |
| | | | CE | 38.00 | 38.00 | |
| | | | CXP | 13.00 | 13.00 | |

| Q1 Mass (Da) | Q3 Mass (Da) | Time (min) | Param | Start | Stop | ID |
|---|---|---|---|---|---|---|
| 704.000 | 308.000 | 5.85 | DP | 80.00 | 80.00 | H(C20)26:0 |
| | | | CE | 35.00 | 35.00 | |
| | | | CXP | 13.00 | 13.00 | |

| Q1 Mass (Da) | Q3 Mass (Da) | Time (min) | Param | Start | Stop | ID |
|---|---|---|---|---|---|---|
| 718.000 | 308.000 | 5.86 | DP | 80.00 | 80.00 | H(C20)27:0 |
| | | | CE | 36.00 | 36.00 | |
| | | | CXP | 13.00 | 13.00 | |

| Q1 Mass (Da) | Q3 Mass (Da) | Time (min) | Param | Start | Stop | ID |
|---|---|---|---|---|---|---|
| 732.000 | 308.000 | 5.87 | DP | 80.00 | 80.00 | H(C20)28:0 |
| | | | CE | 35.00 | 35.00 | |
| | | | CXP | 13.00 | 13.00 | |

| Q1 Mass (Da) | Q3 Mass (Da) | Time (min) | Param | Start | Stop | ID |
|---|---|---|---|---|---|---|
| 746.000 | 308.000 | 5.89 | DP | 80.00 | 80.00 | H(C20)29:0 |
| | | | CE | 40.00 | 40.00 | |
| | | | CXP | 13.00 | 13.00 | |

| Q1 Mass (Da) | Q3 Mass (Da) | Time (min) | Param | Start | Stop | ID |
|---|---|---|---|---|---|---|
| 760.000 | 308.000 | 5.91 | DP | 80.00 | 80.00 | H(C20)30:0 |
| | | | CE | 35.00 | 35.00 | |
| | | | CXP | 13.00 | 13.00 | |

| Q1 Mass (Da) | Q3 Mass (Da) | Time (min) | Param | Start | Stop | ID |
|---|---|---|---|---|---|---|
| 704.000 | 336.000 | 5.89 | DP | 80.00 | 80.00 | H(C22)24:0 |
| | | | CE | 30.00 | 30.00 | |
| | | | CXP | 13.00 | 13.00 | |

| Q1 Mass (Da) | Q3 Mass (Da) | Time (min) | Param | Start | Stop | ID |
|---|---|---|---|---|---|---|
| 718.000 | 336.000 | 5.90 | DP | 80.00 | 80.00 | H(C22)25:0 |
| | | | CE | 34.00 | 34.00 | |
| | | | CXP | 13.00 | 13.00 | |

| Q1 Mass (Da) | Q3 Mass (Da) | Time (min) | Param | Start | Stop | ID |
|---|---|---|---|---|---|---|
| 732.000 | 336.000 | 5.92 | DP | 80.00 | 80.00 | H(C22)26:0 |
| | | | CE | 33.00 | 33.00 | |
| | | | CXP | 13.00 | 13.00 | |

| Q1 Mass (Da) | Q3 Mass (Da) | Time (min) | Param | Start | Stop | ID |
|---|---|---|---|---|---|---|
| 746.000 | 336.000 | 5.92 | DP | 80.00 | 80.00 | H(C22)27:0 |
| | | | CE | 40.00 | 40.00 | |
| | | | CXP | 13.00 | 13.00 | |

| Q1 Mass (Da) | Q3 Mass (Da) | Time (min) | Param | Start | Stop | ID |
|---|---|---|---|---|---|---|
| 640.000 | 254.000 | 4.67 | DP | 40.00 | 40.00 | P(C16)24:0 |
| | | | CE | 41.00 | 41.00 | |
| | | | CXP | 13.00 | 13.00 | |

| Q1 Mass (Da) | Q3 Mass (Da) | Time (min) | Param | Start | Stop | ID |
|---|---|---|---|---|---|---|
| 654.000 | 254.000 | 4.69 | DP | 40.00 | 40.00 | P(C16)25:0 |
| | | | CE | 41.00 | 41.00 | |
| | | | CXP | 13.00 | 13.00 | |

| Q1 Mass (Da) | Q3 Mass (Da) | Time (min) | Param | Start | Stop | ID |
|---|---|---|---|---|---|---|
| 668.000 | 254.000 | 4.70 | DP | 40.00 | 40.00 | P(C16)26:0 |
| | | | CE | 41.00 | 41.00 | |
| | | | CXP | 13.00 | 13.00 | |

| Q1 Mass (Da) | Q3 Mass (Da) | Time (min) | Param | Start | Stop | ID |
|---|---|---|---|---|---|---|
| 682.000 | 254.000 | 4.74 | DP | 40.00 | 40.00 | P(C16)27:0 |
| | | | CE | 45.00 | 45.00 | |
| | | | CXP | 13.00 | 13.00 | |

| Q1 Mass (Da) | Q3 Mass (Da) | Time (min) | Param | Start | Stop | ID |
|---|---|---|---|---|---|---|
| 696.000 | 254.000 | 4.77 | DP | 40.00 | 40.00 | P(C16)28:0 |
| | | | CE | 60.00 | 60.00 | |
| | | | CXP | 13.00 | 13.00 | |

| Q1 Mass (Da) | Q3 Mass (Da) | Time (min) | Param | Start | Stop | ID |
|---|---|---|---|---|---|---|

| | | | | | | |
|---|---|---|---|---|---|---|
| 626.000 | 268.000 | 4.67 | DP | 40.00 | 40.00 | P(C17)22:0 |
| | | | CE | 34.00 | 34.00 | |
| | | | CXP | 13.00 | 13.00 | |

| Q1 Mass (Da) | Q3 Mass (Da) | Time (min) | Param | Start | Stop | ID |
|---|---|---|---|---|---|---|
| 654.000 | 268.000 | 4.71 | DP | 40.00 | 40.00 | P(C17)24:0 |
| | | | CE | 36.00 | 36.00 | |
| | | | CXP | 13.00 | 13.00 | |

| Q1 Mass (Da) | Q3 Mass (Da) | Time (min) | Param | Start | Stop | ID |
|---|---|---|---|---|---|---|
| 670.000 | 250.000 | 4.72 | DP | 40.00 | 40.00 | P(C17)25:0 |
| | | | CE | 48.00 | 48.00 | |
| | | | CXP | 13.00 | 13.00 | |

| Q1 Mass (Da) | Q3 Mass (Da) | Time (min) | Param | Start | Stop | ID |
|---|---|---|---|---|---|---|
| 682.000 | 268.000 | 4.74 | DP | 40.00 | 40.00 | P(C17)26:0 |
| | | | CE | 35.00 | 35.00 | |
| | | | CXP | 13.00 | 13.00 | |

| Q1 Mass (Da) | Q3 Mass (Da) | Time (min) | Param | Start | Stop | ID |
|---|---|---|---|---|---|---|
| 696.000 | 268.000 | 4.75 | DP | 40.00 | 40.00 | P(C17)27:0 |
| | | | CE | 48.00 | 48.00 | |
| | | | CXP | 13.00 | 13.00 | |

| Q1 Mass (Da) | Q3 Mass (Da) | Time (min) | Param | Start | Stop | ID |
|---|---|---|---|---|---|---|
| 710.000 | 268.000 | 4.76 | DP | 40.00 | 40.00 | P(C17)28:0 |

| Q1 Mass (Da) | Q3 Mass (Da) | Time (min) | Param | Start | Stop | ID |
|---|---|---|---|---|---|---|
| | | | CE | 46.00 | 46.00 | |
| | | | CXP | 13.00 | 13.00 | |

| Q1 Mass (Da) | Q3 Mass (Da) | Time (min) | Param | Start | Stop | ID |
|---|---|---|---|---|---|---|
| 584.400 | 264.300 | 4.63 | DP | 40.00 | 40.00 | P(C18)18:0 |
| | | | CE | 34.00 | 34.00 | |
| | | | CXP | 13.00 | 13.00 | |

| Q1 Mass (Da) | Q3 Mass (Da) | Time (min) | Param | Start | Stop | ID |
|---|---|---|---|---|---|---|
| 612.000 | 282.000 | 4.68 | DP | 40.00 | 40.00 | P(C18)20:0 |
| | | | CE | 33.00 | 33.00 | |
| | | | CXP | 13.00 | 13.00 | |

| Q1 Mass (Da) | Q3 Mass (Da) | Time (min) | Param | Start | Stop | ID |
|---|---|---|---|---|---|---|
| 640.000 | 282.000 | 4.71 | DP | 40.00 | 40.00 | P(C18)22:0 |
| | | | CE | 39.00 | 39.00 | |
| | | | CXP | 13.00 | 13.00 | |

| Q1 Mass (Da) | Q3 Mass (Da) | Time (min) | Param | Start | Stop | ID |
|---|---|---|---|---|---|---|
| 654.000 | 282.000 | 4.73 | DP | 40.00 | 40.00 | P(C18)23:0 |
| | | | CE | 35.00 | 35.00 | |
| | | | CXP | 13.00 | 13.00 | |

| Q1 Mass (Da) | Q3 Mass (Da) | Time (min) | Param | Start | Stop | ID |
|---|---|---|---|---|---|---|
| 668.000 | 282.000 | 4.75 | DP | 40.00 | 40.00 | P(C18)24:0 |
| | | | CE | 40.00 | 40.00 | |

| | | | | | | |
|---|---|---|---|---|---|---|
| | | | CXP | 13.00 | 13.00 | |

| Q1 Mass (Da) | Q3 Mass (Da) | Time (min) | Param | Start | Stop | ID |
|---|---|---|---|---|---|---|
| 682.000 | 282.000 | 4.77 | DP | 40.00 | 40.00 | P(C18)25:0 |
| | | | CE | 45.00 | 45.00 | |
| | | | CXP | 13.00 | 13.00 | |

| Q1 Mass (Da) | Q3 Mass (Da) | Time (min) | Param | Start | Stop | ID |
|---|---|---|---|---|---|---|
| 696.000 | 282.000 | 4.78 | DP | 40.00 | 40.00 | P(C18)26:0 |
| | | | CE | 45.00 | 45.00 | |
| | | | CXP | 13.00 | 13.00 | |

| Q1 Mass (Da) | Q3 Mass (Da) | Time (min) | Param | Start | Stop | ID |
|---|---|---|---|---|---|---|
| 710.000 | 282.000 | 4.80 | DP | 40.00 | 40.00 | P(C18)27:0 |
| | | | CE | 40.00 | 40.00 | |
| | | | CXP | 13.00 | 13.00 | |

| Q1 Mass (Da) | Q3 Mass (Da) | Time (min) | Param | Start | Stop | ID |
|---|---|---|---|---|---|---|
| 724.000 | 282.000 | 4.81 | DP | 40.00 | 40.00 | P(C18)28:0 |
| | | | CE | 50.00 | 50.00 | |
| | | | CXP | 13.00 | 13.00 | |

| Q1 Mass (Da) | Q3 Mass (Da) | Time (min) | Param | Start | Stop | ID |
|---|---|---|---|---|---|---|
| 738.000 | 282.000 | 4.77 | DP | 40.00 | 40.00 | P(C18)29:0 |
| | | | CE | 41.00 | 41.00 | |
| | | | CXP | 13.00 | 13.00 | |

| Q1 Mass (Da) | Q3 Mass (Da) | Time (min) | Param | Start | Stop | ID |
|---|---|---|---|---|---|---|
| 682.000 | 296.000 | 4.79 | DP | 40.00 | 40.00 | P(C19)24:0 |
| | | | CE | 40.00 | 40.00 | |
| | | | CXP | 13.00 | 13.00 | |

| Q1 Mass (Da) | Q3 Mass (Da) | Time (min) | Param | Start | Stop | ID |
|---|---|---|---|---|---|---|
| 696.000 | 296.000 | 4.80 | DP | 40.00 | 40.00 | P(C19)25:0 |
| | | | CE | 54.00 | 54.00 | |
| | | | CXP | 13.00 | 13.00 | |

| Q1 Mass (Da) | Q3 Mass (Da) | Time (min) | Param | Start | Stop | ID |
|---|---|---|---|---|---|---|
| 710.000 | 296.000 | 4.82 | DP | 40.00 | 40.00 | P(C19)26:0 |
| | | | CE | 46.00 | 46.00 | |
| | | | CXP | 13.00 | 13.00 | |

| Q1 Mass (Da) | Q3 Mass (Da) | Time (min) | Param | Start | Stop | ID |
|---|---|---|---|---|---|---|
| 682.000 | 310.000 | 4.78 | DP | 40.00 | 40.00 | P(C20)23:0 |
| | | | CE | 45.00 | 45.00 | |
| | | | CXP | 13.00 | 13.00 | |

| Q1 Mass (Da) | Q3 Mass (Da) | Time (min) | Param | Start | Stop | ID |
|---|---|---|---|---|---|---|
| 696.000 | 310.000 | 4.83 | DP | 40.00 | 40.00 | P(C20)24:0 |
| | | | CE | 40.00 | 40.00 | |
| | | | CXP | 13.00 | 13.00 | |

| Q1 Mass (Da) | Q3 Mass (Da) | Time (min) | Param | Start | Stop | ID |
|---|---|---|---|---|---|---|
| 710.000 | 310.000 | 4.84 | DP | 40.00 | 40.00 | P(C20)25:0 |
| | | | CE | 47.00 | 47.00 | |
| | | | CXP | 13.00 | 13.00 | |

| Q1 Mass (Da) | Q3 Mass (Da) | Time (min) | Param | Start | Stop | ID |
|---|---|---|---|---|---|---|
| 724.000 | 310.000 | 4.85 | DP | 40.00 | 40.00 | P(C20)26:0 |
| | | | CE | 30.00 | 30.00 | |
| | | | CXP | 13.00 | 13.00 | |

| Q1 Mass (Da) | Q3 Mass (Da) | Time (min) | Param | Start | Stop | ID |
|---|---|---|---|---|---|---|
| 738.000 | 310.000 | 4.85 | DP | 40.00 | 40.00 | P(C20)27:0 |
| | | | CE | 44.00 | 44.00 | |
| | | | CXP | 13.00 | 13.00 | |

| Q1 Mass (Da) | Q3 Mass (Da) | Time (min) | Param | Start | Stop | ID |
|---|---|---|---|---|---|---|
| 752.000 | 310.000 | 4.89 | DP | 40.00 | 40.00 | P(C20)28:0 |
| | | | CE | 45.00 | 45.00 | |
| | | | CXP | 13.00 | 13.00 | |

| Q1 Mass (Da) | Q3 Mass (Da) | Time (min) | Param | Start | Stop | ID |
|---|---|---|---|---|---|---|
| 710.000 | 324.000 | 4.85 | DP | 40.00 | 40.00 | P(C21)24:0 |
| | | | CE | 45.00 | 45.00 | |
| | | | CXP | 13.00 | 13.00 | |

| Q1 Mass (Da) | Q3 Mass (Da) | Time (min) | Param | Start | Stop | ID |
|---|---|---|---|---|---|---|
| 724.000 | 324.000 | 4.88 | DP | 40.00 | 40.00 | P(C21)25:0 |
| | | | CE | 55.00 | 55.00 | |
| | | | CXP | 13.00 | 13.00 | |

| Q1 Mass (Da) | Q3 Mass (Da) | Time (min) | Param | Start | Stop | ID |
|---|---|---|---|---|---|---|
| 738.000 | 324.000 | 4.87 | DP | 40.00 | 40.00 | P(C21)26:0 |
| | | | CE | 31.00 | 31.00 | |
| | | | CXP | 13.00 | 13.00 | |

| Q1 Mass (Da) | Q3 Mass (Da) | Time (min) | Param | Start | Stop | ID |
|---|---|---|---|---|---|---|
| 696.000 | 338.000 | 4.85 | DP | 40.00 | 40.00 | P(C22)22:0 |
| | | | CE | 40.00 | 40.00 | |
| | | | CXP | 13.00 | 13.00 | |

| Q1 Mass (Da) | Q3 Mass (Da) | Time (min) | Param | Start | Stop | ID |
|---|---|---|---|---|---|---|
| 724.000 | 338.000 | 4.88 | DP | 40.00 | 40.00 | P(C22)24:0 |
| | | | CE | 50.00 | 50.00 | |
| | | | CXP | 13.00 | 13.00 | |

| Q1 Mass (Da) | Q3 Mass (Da) | Time (min) | Param | Start | Stop | ID |
|---|---|---|---|---|---|---|
| 738.000 | 338.000 | 4.90 | DP | 40.00 | 40.00 | P(C22)25:0 |
| | | | CE | 43.00 | 43.00 | |
| | | | CXP | 13.00 | 13.00 | |

| Q1 Mass (Da) | Q3 Mass (Da) | Time (min) | Param | Start | Stop | ID |
|---|---|---|---|---|---|---|
| 752.000 | 338.000 | 4.93 | DP | 40.00 | 40.00 | P(C22)26:0 |
| | | | CE | 50.00 | 50.00 | |
| | | | CXP | 13.00 | 13.00 | |

| Q1 Mass (Da) | Q3 Mass (Da) | Time (min) | Param | Start | Stop | ID |
|---|---|---|---|---|---|---|
| 780.000 | 338.000 | 5.00 | DP | 40.00 | 40.00 | P(C22)28:0 |
| | | | CE | 42.00 | 42.00 | |
| | | | CXP | 13.00 | 13.00 | |

| Q1 Mass (Da) | Q3 Mass (Da) | Time (min) | Param | Start | Stop | ID |
|---|---|---|---|---|---|---|
| 752.000 | 366.000 | 4.96 | DP | 40.00 | 40.00 | P(C24)24:0 |
| | | | CE | 50.00 | 50.00 | |
| | | | CXP | 13.00 | 13.00 | |

| Q1 Mass (Da) | Q3 Mass (Da) | Time (min) | Param | Start | Stop | ID |
|---|---|---|---|---|---|---|
| 766.000 | 366.000 | 4.99 | DP | 40.00 | 40.00 | P(C24)25:0 |
| | | | CE | 55.00 | 55.00 | |
| | | | CXP | 13.00 | 13.00 | |

| Q1 Mass (Da) | Q3 Mass (Da) | Time (min) | Param | Start | Stop | ID |
|---|---|---|---|---|---|---|
| 780.000 | 366.000 | 5.01 | DP | 40.00 | 40.00 | P(C24)26:0 |
| | | | CE | 45.00 | 45.00 | |
| | | | CXP | 13.00 | 13.00 | |

| Q1 Mass (Da) | Q3 Mass (Da) | Time (min) | Param | Start | Stop | ID |
|---|---|---|---|---|---|---|

| Q1 Mass (Da) | Q3 Mass (Da) | Time (min) | Param | Start | Stop | ID |
|---|---|---|---|---|---|---|
| 608.000 | 236.000 | 3.89 | DP | 80.00 | 80.00 | S(C16)23:0 |
| | | | CE | 42.00 | 42.00 | |
| | | | CXP | 13.00 | 13.00 | |

| Q1 Mass (Da) | Q3 Mass (Da) | Time (min) | Param | Start | Stop | ID |
|---|---|---|---|---|---|---|
| 622.000 | 236.000 | 3.90 | DP | 80.00 | 80.00 | S(C16)24:0 |
| | | | CE | 36.00 | 36.00 | |
| | | | CXP | 13.00 | 13.00 | |

| Q1 Mass (Da) | Q3 Mass (Da) | Time (min) | Param | Start | Stop | ID |
|---|---|---|---|---|---|---|
| 636.000 | 236.000 | 3.91 | DP | 80.00 | 80.00 | S(C16)25:0 |
| | | | CE | 36.00 | 36.00 | |
| | | | CXP | 13.00 | 13.00 | |

| Q1 Mass (Da) | Q3 Mass (Da) | Time (min) | Param | Start | Stop | ID |
|---|---|---|---|---|---|---|
| 650.000 | 236.000 | 3.94 | DP | 80.00 | 80.00 | S(C16)26:0 |
| | | | CE | 39.00 | 39.00 | |
| | | | CXP | 13.00 | 13.00 | |

| Q1 Mass (Da) | Q3 Mass (Da) | Time (min) | Param | Start | Stop | ID |
|---|---|---|---|---|---|---|
| 664.000 | 236.000 | 3.93 | DP | 80.00 | 80.00 | S(C16)27:0 |
| | | | CE | 33.00 | 33.00 | |
| | | | CXP | 13.00 | 13.00 | |

| Q1 Mass (Da) | Q3 Mass (Da) | Time (min) | Param | Start | Stop | ID |
|---|---|---|---|---|---|---|
| 678.000 | 236.000 | 3.94 | DP | 80.00 | 80.00 | S(C16)28:0 |

| Q1 Mass (Da) | Q3 Mass (Da) | Time (min) | Param | Start | Stop | ID |
|---|---|---|---|---|---|---|
| | | | CE | 60.00 | 60.00 | |
| | | | CXP | 13.00 | 13.00 | |

| Q1 Mass (Da) | Q3 Mass (Da) | Time (min) | Param | Start | Stop | ID |
|---|---|---|---|---|---|---|
| 636.000 | 250.000 | 3.93 | DP | 80.00 | 80.00 | S(C17)24:0 |
| | | | CE | 43.00 | 43.00 | |
| | | | CXP | 13.00 | 13.00 | |

| Q1 Mass (Da) | Q3 Mass (Da) | Time (min) | Param | Start | Stop | ID |
|---|---|---|---|---|---|---|
| 650.000 | 250.000 | 3.95 | DP | 80.00 | 80.00 | S(C17)25:0 |
| | | | CE | 55.00 | 55.00 | |
| | | | CXP | 13.00 | 13.00 | |

| Q1 Mass (Da) | Q3 Mass (Da) | Time (min) | Param | Start | Stop | ID |
|---|---|---|---|---|---|---|
| 664.000 | 250.000 | 3.96 | DP | 80.00 | 80.00 | S(C17)26:0 |
| | | | CE | 39.00 | 39.00 | |
| | | | CXP | 13.00 | 13.00 | |

| Q1 Mass (Da) | Q3 Mass (Da) | Time (min) | Param | Start | Stop | ID |
|---|---|---|---|---|---|---|
| 678.000 | 250.000 | 3.97 | DP | 80.00 | 80.00 | S(C17)27:0 |
| | | | CE | 39.00 | 39.00 | |
| | | | CXP | 13.00 | 13.00 | |

| Q1 Mass (Da) | Q3 Mass (Da) | Time (min) | Param | Start | Stop | ID |
|---|---|---|---|---|---|---|
| 692.000 | 250.000 | 4.01 | DP | 80.00 | 80.00 | S(C17)28:0 |
| | | | CE | 41.00 | 41.00 | |

| | | | | | | |
|---|---|---|---|---|---|---|
| | | | CXP | 13.00 | 13.00 | |

| Q1 Mass (Da) | Q3 Mass (Da) | Time (min) | Param | Start | Stop | ID |
|---|---|---|---|---|---|---|
| 510.300 | 264.200 | 3.72 | DP | 80.00 | 80.00 | S(C18)14:0 |
| | | | CE | 35.00 | 35.00 | |
| | | | CXP | 13.00 | 13.00 | |

| Q1 Mass (Da) | Q3 Mass (Da) | Time (min) | Param | Start | Stop | ID |
|---|---|---|---|---|---|---|
| 538.300 | 264.200 | 3.79 | DP | 80.00 | 80.00 | S(C18)16:0 |
| | | | CE | 31.00 | 31.00 | |
| | | | CXP | 13.00 | 13.00 | |

| Q1 Mass (Da) | Q3 Mass (Da) | Time (min) | Param | Start | Stop | ID |
|---|---|---|---|---|---|---|
| 552.000 | 264.000 | 3.75 | DP | 80.00 | 80.00 | S(C18)17:0 |
| | | | CE | 29.00 | 29.00 | |
| | | | CXP | 13.00 | 13.00 | |

| Q1 Mass (Da) | Q3 Mass (Da) | Time (min) | Param | Start | Stop | ID |
|---|---|---|---|---|---|---|
| 566.300 | 264.200 | 3.77 | DP | 80.00 | 80.00 | S(C18)18:0 |
| | | | CE | 31.00 | 31.00 | |
| | | | CXP | 13.00 | 13.00 | |

| Q1 Mass (Da) | Q3 Mass (Da) | Time (min) | Param | Start | Stop | ID |
|---|---|---|---|---|---|---|
| 594.300 | 264.200 | 3.89 | DP | 80.00 | 80.00 | S(C18)20:0 |
| | | | CE | 49.00 | 49.00 | |
| | | | CXP | 13.00 | 13.00 | |

| Q1 Mass (Da) | Q3 Mass (Da) | Time (min) | Param | Start | Stop | ID |
|---|---|---|---|---|---|---|
| 608.000 | 264.000 | 3.92 | DP | 80.00 | 80.00 | S(C18)21:0 |
| | | | CE | 42.00 | 42.00 | |
| | | | CXP | 13.00 | 13.00 | |

| Q1 Mass (Da) | Q3 Mass (Da) | Time (min) | Param | Start | Stop | ID |
|---|---|---|---|---|---|---|
| 622.000 | 264.000 | 3.94 | DP | 80.00 | 80.00 | S(C18)22:0 |
| | | | CE | 41.00 | 41.00 | |
| | | | CXP | 13.00 | 13.00 | |

| Q1 Mass (Da) | Q3 Mass (Da) | Time (min) | Param | Start | Stop | ID |
|---|---|---|---|---|---|---|
| 636.000 | 264.000 | 3.96 | DP | 80.00 | 80.00 | S(C18)23:0 |
| | | | CE | 55.00 | 55.00 | |
| | | | CXP | 13.00 | 13.00 | |

| Q1 Mass (Da) | Q3 Mass (Da) | Time (min) | Param | Start | Stop | ID |
|---|---|---|---|---|---|---|
| 650.000 | 264.000 | 3.97 | DP | 80.00 | 80.00 | S(C18)24:0 |
| | | | CE | 40.00 | 40.00 | |
| | | | CXP | 13.00 | 13.00 | |

| Q1 Mass (Da) | Q3 Mass (Da) | Time (min) | Param | Start | Stop | ID |
|---|---|---|---|---|---|---|
| 664.000 | 264.000 | 3.99 | DP | 80.00 | 80.00 | S(C18)25:0 |
| | | | CE | 41.00 | 41.00 | |
| | | | CXP | 13.00 | 13.00 | |

| Q1 Mass (Da) | Q3 Mass (Da) | Time (min) | Param | Start | Stop | ID |
|---|---|---|---|---|---|---|
| 678.000 | 264.000 | 4.01 | DP | 80.00 | 80.00 | S(C18)26:0 |
| | | | CE | 37.00 | 37.00 | |
| | | | CXP | 13.00 | 13.00 | |

| Q1 Mass (Da) | Q3 Mass (Da) | Time (min) | Param | Start | Stop | ID |
|---|---|---|---|---|---|---|
| 692.000 | 264.000 | 4.03 | DP | 80.00 | 80.00 | S(C18)27:0 |
| | | | CE | 40.00 | 40.00 | |
| | | | CXP | 13.00 | 13.00 | |

| Q1 Mass (Da) | Q3 Mass (Da) | Time (min) | Param | Start | Stop | ID |
|---|---|---|---|---|---|---|
| 706.000 | 264.000 | 4.04 | DP | 80.00 | 80.00 | S(C18)28:0 |
| | | | CE | 51.00 | 51.00 | |
| | | | CXP | 13.00 | 13.00 | |

| Q1 Mass (Da) | Q3 Mass (Da) | Time (min) | Param | Start | Stop | ID |
|---|---|---|---|---|---|---|
| 720.000 | 264.000 | 4.03 | DP | 80.00 | 80.00 | S(C18)29:0 |
| | | | CE | 55.00 | 55.00 | |
| | | | CXP | 13.00 | 13.00 | |

| Q1 Mass (Da) | Q3 Mass (Da) | Time (min) | Param | Start | Stop | ID |
|---|---|---|---|---|---|---|
| 734.000 | 264.000 | 4.09 | DP | 80.00 | 80.00 | S(C18)30:0 |
| | | | CE | 41.00 | 41.00 | |
| | | | CXP | 13.00 | 13.00 | |

| Q1 Mass (Da) | Q3 Mass (Da) | Time (min) | Param | Start | Stop | ID |
|---|---|---|---|---|---|---|
| 664.000 | 278.000 | 4.01 | DP | 80.00 | 80.00 | S(C19)24:0 |
| | | | CE | 50.00 | 50.00 | |
| | | | CXP | 13.00 | 13.00 | |

| Q1 Mass (Da) | Q3 Mass (Da) | Time (min) | Param | Start | Stop | ID |
|---|---|---|---|---|---|---|
| 678.000 | 278.000 | 4.03 | DP | 80.00 | 80.00 | S(C19)25:0 |
| | | | CE | 36.00 | 36.00 | |
| | | | CXP | 13.00 | 13.00 | |

| Q1 Mass (Da) | Q3 Mass (Da) | Time (min) | Param | Start | Stop | ID |
|---|---|---|---|---|---|---|
| 692.000 | 278.000 | 4.04 | DP | 80.00 | 80.00 | S(C19)26:0 |
| | | | CE | 45.00 | 45.00 | |
| | | | CXP | 13.00 | 13.00 | |

| Q1 Mass (Da) | Q3 Mass (Da) | Time (min) | Param | Start | Stop | ID |
|---|---|---|---|---|---|---|
| 706.000 | 278.000 | 4.06 | DP | 80.00 | 80.00 | S(C19)27:0 |
| | | | CE | 40.00 | 40.00 | |
| | | | CXP | 13.00 | 13.00 | |

| Q1 Mass (Da) | Q3 Mass (Da) | Time (min) | Param | Start | Stop | ID |
|---|---|---|---|---|---|---|
| 720.000 | 278.000 | 4.09 | DP | 80.00 | 80.00 | S(C19)28:0 |
| | | | CE | 39.00 | 39.00 | |
| | | | CXP | 13.00 | 13.00 | |

| Q1 Mass (Da) | Q3 Mass (Da) | Time (min) | Param | Start | Stop | ID |
|---|---|---|---|---|---|---|
| 650.000 | 292.000 | 4.00 | DP | 80.00 | 80.00 | S(C20)22:0 |
| | | | CE | 45.00 | 45.00 | |
| | | | CXP | 13.00 | 13.00 | |

| Q1 Mass (Da) | Q3 Mass (Da) | Time (min) | Param | Start | Stop | ID |
|---|---|---|---|---|---|---|
| 678.000 | 292.000 | 4.04 | DP | 80.00 | 80.00 | S(C20)24:0 |
| | | | CE | 37.00 | 37.00 | |
| | | | CXP | 13.00 | 13.00 | |

| Q1 Mass (Da) | Q3 Mass (Da) | Time (min) | Param | Start | Stop | ID |
|---|---|---|---|---|---|---|
| 692.000 | 292.000 | 4.06 | DP | 80.00 | 80.00 | S(C20)25:0 |
| | | | CE | 45.00 | 45.00 | |
| | | | CXP | 13.00 | 13.00 | |

| Q1 Mass (Da) | Q3 Mass (Da) | Time (min) | Param | Start | Stop | ID |
|---|---|---|---|---|---|---|
| 706.000 | 292.000 | 4.10 | DP | 80.00 | 80.00 | S(C20)26:0 |
| | | | CE | 44.00 | 44.00 | |
| | | | CXP | 13.00 | 13.00 | |

| Q1 Mass (Da) | Q3 Mass (Da) | Time (min) | Param | Start | Stop | ID |
|---|---|---|---|---|---|---|
| 720.000 | 292.000 | 4.11 | DP | 80.00 | 80.00 | S(C20)27:0 |
| | | | CE | 43.00 | 43.00 | |
| | | | CXP | 13.00 | 13.00 | |

| Q1 Mass (Da) | Q3 Mass (Da) | Time (min) | Param | Start | Stop | ID |
|---|---|---|---|---|---|---|

| Q1 Mass (Da) | Q3 Mass (Da) | Time (min) | Param | Start | Stop | ID |
|---|---|---|---|---|---|---|
| 734.000 | 292.000 | 4.12 | DP | 80.00 | 80.00 | S(C20)28:0 |
| | | | CE | 29.00 | 29.00 | |
| | | | CXP | 13.00 | 13.00 | |

| Q1 Mass (Da) | Q3 Mass (Da) | Time (min) | Param | Start | Stop | ID |
|---|---|---|---|---|---|---|
| 748.000 | 292.000 | 4.11 | DP | 80.00 | 80.00 | S(C20)29:0 |
| | | | CE | 35.00 | 35.00 | |
| | | | CXP | 13.00 | 13.00 | |

| Q1 Mass (Da) | Q3 Mass (Da) | Time (min) | Param | Start | Stop | ID |
|---|---|---|---|---|---|---|
| 762.000 | 292.000 | 4.17 | DP | 80.00 | 80.00 | S(C20)30:0 |
| | | | CE | 36.00 | 36.00 | |
| | | | CXP | 13.00 | 13.00 | |

| Q1 Mass (Da) | Q3 Mass (Da) | Time (min) | Param | Start | Stop | ID |
|---|---|---|---|---|---|---|
| 790.000 | 292.000 | 4.18 | DP | 80.00 | 80.00 | S(C20)32:0 |
| | | | CE | 39.00 | 39.00 | |
| | | | CXP | 13.00 | 13.00 | |

| Q1 Mass (Da) | Q3 Mass (Da) | Time (min) | Param | Start | Stop | ID |
|---|---|---|---|---|---|---|
| 692.000 | 306.000 | 4.07 | DP | 80.00 | 80.00 | S(C21)24:0 |
| | | | CE | 47.00 | 47.00 | |
| | | | CXP | 13.00 | 13.00 | |

| Q1 Mass (Da) | Q3 Mass (Da) | Time (min) | Param | Start | Stop | ID |
|---|---|---|---|---|---|---|
| 706.000 | 306.000 | 4.07 | DP | 80.00 | 80.00 | S(C21)25:0 |

| Q1 Mass (Da) | Q3 Mass (Da) | Time (min) | Param | Start | Stop | ID |
|---|---|---|---|---|---|---|
| | | | CE | 40.00 | 40.00 | |
| | | | CXP | 13.00 | 13.00 | |

| Q1 Mass (Da) | Q3 Mass (Da) | Time (min) | Param | Start | Stop | ID |
|---|---|---|---|---|---|---|
| 720.000 | 306.000 | 4.10 | DP | 80.00 | 80.00 | S(C21)26:0 |
| | | | CE | 51.00 | 51.00 | |
| | | | CXP | 13.00 | 13.00 | |

| Q1 Mass (Da) | Q3 Mass (Da) | Time (min) | Param | Start | Stop | ID |
|---|---|---|---|---|---|---|
| 734.000 | 306.000 | 4.14 | DP | 80.00 | 80.00 | S(C21)27:0 |
| | | | CE | 39.00 | 39.00 | |
| | | | CXP | 13.00 | 13.00 | |

| Q1 Mass (Da) | Q3 Mass (Da) | Time (min) | Param | Start | Stop | ID |
|---|---|---|---|---|---|---|
| 748.000 | 306.000 | 4.14 | DP | 80.00 | 80.00 | S(C21)28:0 |
| | | | CE | 39.00 | 39.00 | |
| | | | CXP | 13.00 | 13.00 | |

| Q1 Mass (Da) | Q3 Mass (Da) | Time (min) | Param | Start | Stop | ID |
|---|---|---|---|---|---|---|
| 762.000 | 306.000 | 4.17 | DP | 80.00 | 80.00 | S(C21)29:0 |
| | | | CE | 55.00 | 55.00 | |
| | | | CXP | 13.00 | 13.00 | |

| Q1 Mass (Da) | Q3 Mass (Da) | Time (min) | Param | Start | Stop | ID |
|---|---|---|---|---|---|---|
| 776.000 | 306.000 | 4.18 | DP | 80.00 | 80.00 | S(C21)30:0 |
| | | | CE | 55.00 | 55.00 | |

| | | | Param | Start | Stop | |
|---|---|---|---|---|---|---|
| | | | CXP | 13.00 | 13.00 | |

| Q1 Mass (Da) | Q3 Mass (Da) | Time (min) | Param | Start | Stop | ID |
|---|---|---|---|---|---|---|
| 790.000 | 306.000 | 4.25 | DP | 80.00 | 80.00 | S(C21)31:0 |
| | | | CE | 46.00 | 46.00 | |
| | | | CXP | 13.00 | 13.00 | |

| Q1 Mass (Da) | Q3 Mass (Da) | Time (min) | Param | Start | Stop | ID |
|---|---|---|---|---|---|---|
| 804.000 | 306.000 | 4.31 | DP | 80.00 | 80.00 | S(C21)32:0 |
| | | | CE | 43.00 | 43.00 | |
| | | | CXP | 13.00 | 13.00 | |

| Q1 Mass (Da) | Q3 Mass (Da) | Time (min) | Param | Start | Stop | ID |
|---|---|---|---|---|---|---|
| 706.000 | 320.000 | 4.10 | DP | 80.00 | 80.00 | S(C22)24:0 |
| | | | CE | 49.00 | 49.00 | |
| | | | CXP | 13.00 | 13.00 | |

| Q1 Mass (Da) | Q3 Mass (Da) | Time (min) | Param | Start | Stop | ID |
|---|---|---|---|---|---|---|
| 720.000 | 320.000 | 4.14 | DP | 80.00 | 80.00 | S(C22)25:0 |
| | | | CE | 31.00 | 31.00 | |
| | | | CXP | 13.00 | 13.00 | |

| Q1 Mass (Da) | Q3 Mass (Da) | Time (min) | Param | Start | Stop | ID |
|---|---|---|---|---|---|---|
| 734.000 | 320.000 | 4.15 | DP | 80.00 | 80.00 | S(C22)26:0 |
| | | | CE | 41.00 | 41.00 | |
| | | | CXP | 13.00 | 13.00 | |

| Q1 Mass (Da) | Q3 Mass (Da) | Time (min) | Param | Start | Stop | ID |
|---|---|---|---|---|---|---|
| 748.000 | 320.000 | 4.18 | DP | 80.00 | 80.00 | S(C22)27:0 |
| | | | CE | 49.00 | 49.00 | |
| | | | CXP | 13.00 | 13.00 | |

| Q1 Mass (Da) | Q3 Mass (Da) | Time (min) | Param | Start | Stop | ID |
|---|---|---|---|---|---|---|
| 762.000 | 320.000 | 4.20 | DP | 80.00 | 80.00 | S(C22)28:0 |
| | | | CE | 40.00 | 40.00 | |
| | | | CXP | 13.00 | 13.00 | |

| Q1 Mass (Da) | Q3 Mass (Da) | Time (min) | Param | Start | Stop | ID |
|---|---|---|---|---|---|---|
| 776.000 | 320.000 | 4.18 | DP | 80.00 | 80.00 | S(C22)29:0 |
| | | | CE | 36.00 | 36.00 | |
| | | | CXP | 13.00 | 13.00 | |

| Q1 Mass (Da) | Q3 Mass (Da) | Time (min) | Param | Start | Stop | ID |
|---|---|---|---|---|---|---|
| 790.000 | 320.000 | 4.24 | DP | 80.00 | 80.00 | S(C22)30:0 |
| | | | CE | 45.00 | 45.00 | |
| | | | CXP | 13.00 | 13.00 | |

| Q1 Mass (Da) | Q3 Mass (Da) | Time (min) | Param | Start | Stop | ID |
|---|---|---|---|---|---|---|
| 804.000 | 320.000 | 4.30 | DP | 80.00 | 80.00 | S(C22)31:0 |
| | | | CE | 74.00 | 74.00 | |
| | | | CXP | 13.00 | 13.00 | |

| Q1 Mass (Da) | Q3 Mass (Da) | Time (min) | Param | Start | Stop | ID |
|---|---|---|---|---|---|---|
| 818.000 | 320.000 | 4.29 | DP | 80.00 | 80.00 | S(C22)32:0 |
| | | | CE | 54.00 | 54.00 | |
| | | | CXP | 13.00 | 13.00 | |

| Q1 Mass (Da) | Q3 Mass (Da) | Time (min) | Param | Start | Stop | ID |
|---|---|---|---|---|---|---|
| 734.000 | 348.000 | 4.18 | DP | 80.00 | 80.00 | S(C24)24:0 |
| | | | CE | 40.00 | 40.00 | |
| | | | CXP | 13.00 | 13.00 | |

| Q1 Mass (Da) | Q3 Mass (Da) | Time (min) | Param | Start | Stop | ID |
|---|---|---|---|---|---|---|
| 748.000 | 348.000 | 4.19 | DP | 80.00 | 80.00 | S(C24)25:0 |
| | | | CE | 55.00 | 55.00 | |
| | | | CXP | 13.00 | 13.00 | |

| Q1 Mass (Da) | Q3 Mass (Da) | Time (min) | Param | Start | Stop | ID |
|---|---|---|---|---|---|---|
| 762.000 | 348.000 | 4.21 | DP | 80.00 | 80.00 | S(C24)26:0 |
| | | | CE | 41.00 | 41.00 | |
| | | | CXP | 13.00 | 13.00 | |

| Q1 Mass (Da) | Q3 Mass (Da) | Time (min) | Param | Start | Stop | ID |
|---|---|---|---|---|---|---|
| 790.000 | 348.000 | 4.32 | DP | 80.00 | 80.00 | S(C24)28:0 |
| | | | CE | 35.00 | 35.00 | |
| | | | CXP | 13.00 | 13.00 | |

| Q1 Mass (Da) | Q3 Mass (Da) | Time (min) | Param | Start | Stop | ID |
|---|---|---|---|---|---|---|
| 762.000 | 376.000 | 4.24 | DP | 80.00 | 80.00 | S(C26)24:0 |
| | | | CE | 45.00 | 45.00 | |
| | | | CXP | 13.00 | 13.00 | |

| Q1 Mass (Da) | Q3 Mass (Da) | Time (min) | Param | Start | Stop | ID |
|---|---|---|---|---|---|---|
| 776.000 | 376.000 | 4.24 | DP | 80.00 | 80.00 | S(C26)25:0 |
| | | | CE | 51.00 | 51.00 | |
| | | | CXP | 13.00 | 13.00 | |

| Q1 Mass (Da) | Q3 Mass (Da) | Time (min) | Param | Start | Stop | ID |
|---|---|---|---|---|---|---|
| 790.000 | 376.000 | 4.28 | DP | 80.00 | 80.00 | S(C26)26:0 |
| | | | CE | 50.00 | 50.00 | |
| | | | CXP | 13.00 | 13.00 | |

**Parameter Table(Period 1 Experiment 1)**

| | |
|---|---|
| CUR: | 25.00 |
| CAD: | Medium |
| TEM: | 450.00 |
| GS1: | 50.00 |
| GS2: | 60.00 |
| IS: | 4500.00 |
| EP | 10.00 |

**Period 1 Experiment 2:**

----------------------------

| | |
|---|---|
| Scan Type: | MRM (MRM) |
| Scheduled MRM: | Yes |
| Polarity: | Negative |

Scan Mode: N/A
Ion Source: Turbo Spray
MRM detection window: 60 sec
Target Scan Time: 0.5000 sec
Resolution Q1: Unit
Resolution Q3: Unit
Intensity Thres.: 0.00 cps
Settling Time: 50.0000 msec
MR Pause: 5.0070 msec
MCA: No
Step Size: 0.00 Da

| Q1 Mass (Da) | Q3 Mass (Da) | Time (min) | Param | Start | Stop | ID |
|---|---|---|---|---|---|---|
| 472.100 | 80.000 | 9.58 | DP | -100.00 | -100.00 | d7-CholSO4 |
| | | | CE | -110.00 | -110.00 | |
| | | | CXP | -12.00 | -12.00 | |

| Q1 Mass (Da) | Q3 Mass (Da) | Time (min) | Param | Start | Stop | ID |
|---|---|---|---|---|---|---|
| 267.000 | 267.000 | 0.89 | DP | -140.00 | -140.00 | FA17:1_SC |
| | | | CE | -12.00 | -12.00 | |
| | | | CXP | -18.00 | -18.00 | |

| Q1 Mass (Da) | Q3 Mass (Da) | Time (min) | Param | Start | Stop | ID |
|---|---|---|---|---|---|---|
| 297.000 | 297.000 | 0.89 | DP | -160.00 | -160.00 | FA19:0_SC |
| | | | CE | -12.00 | -12.00 | |
| | | | CXP | -20.00 | -20.00 | |

| Q1 Mass (Da) | Q3 Mass (Da) | Time (min) | Param | Start | Stop | ID |
|---|---|---|---|---|---|---|
| 320.900 | 321.200 | 0.93 | DP | -110.00 | -110.00 | FA21:2_SC |
| | | | CE | -17.00 | -17.00 | |

| | | | Param | Start | Stop | ID |
|---|---|---|---|---|---|---|
| | | | CXP | -25.00 | -25.00 | |

| Q1 Mass (Da) | Q3 Mass (Da) | Time (min) | Param | Start | Stop | ID |
|---|---|---|---|---|---|---|
| 255.000 | 255.000 | 0.87 | DP | -160.00 | -160.00 | FA16:0_SC |
| | | | CE | -16.00 | -16.00 | |
| | | | CXP | -16.00 | -16.00 | |

| Q1 Mass (Da) | Q3 Mass (Da) | Time (min) | Param | Start | Stop | ID |
|---|---|---|---|---|---|---|
| 253.000 | 253.000 | 0.88 | DP | -160.00 | -160.00 | FA16:1_SC |
| | | | CE | -17.00 | -17.00 | |
| | | | CXP | -16.00 | -16.00 | |

| Q1 Mass (Da) | Q3 Mass (Da) | Time (min) | Param | Start | Stop | ID |
|---|---|---|---|---|---|---|
| 283.000 | 283.000 | 0.88 | DP | -160.00 | -160.00 | FA18:0_SC |
| | | | CE | -18.00 | -18.00 | |
| | | | CXP | -16.00 | -16.00 | |

| Q1 Mass (Da) | Q3 Mass (Da) | Time (min) | Param | Start | Stop | ID |
|---|---|---|---|---|---|---|
| 281.000 | 281.000 | 0.89 | DP | -160.00 | -160.00 | FA18:1_SC |
| | | | CE | -19.00 | -19.00 | |
| | | | CXP | -16.00 | -16.00 | |

| Q1 Mass (Da) | Q3 Mass (Da) | Time (min) | Param | Start | Stop | ID |
|---|---|---|---|---|---|---|
| 279.000 | 279.000 | 0.91 | DP | -160.00 | -160.00 | FA18:2_SC |
| | | | CE | -21.00 | -21.00 | |
| | | | CXP | -16.00 | -16.00 | |

| Q1 Mass (Da) | Q3 Mass (Da) | Time (min) | Param | Start | Stop | ID |
|---|---|---|---|---|---|---|
| 311.000 | 311.000 | 0.89 | DP | -160.00 | -160.00 | FA20:0_SC |
| | | | CE | -26.00 | -26.00 | |
| | | | CXP | -16.00 | -16.00 | |

| Q1 Mass (Da) | Q3 Mass (Da) | Time (min) | Param | Start | Stop | ID |
|---|---|---|---|---|---|---|
| 309.000 | 309.000 | 0.90 | DP | -160.00 | -160.00 | FA20:1_SC |
| | | | CE | -25.00 | -25.00 | |
| | | | CXP | -16.00 | -16.00 | |

| Q1 Mass (Da) | Q3 Mass (Da) | Time (min) | Param | Start | Stop | ID |
|---|---|---|---|---|---|---|
| 307.000 | 307.000 | 0.91 | DP | -160.00 | -160.00 | FA20:2_SC |
| | | | CE | -25.00 | -25.00 | |
| | | | CXP | -16.00 | -16.00 | |

| Q1 Mass (Da) | Q3 Mass (Da) | Time (min) | Param | Start | Stop | ID |
|---|---|---|---|---|---|---|
| 339.100 | 339.100 | 0.90 | DP | -160.00 | -160.00 | FA22:0_SC |
| | | | CE | -25.00 | -25.00 | |
| | | | CXP | -16.00 | -16.00 | |

| Q1 Mass (Da) | Q3 Mass (Da) | Time (min) | Param | Start | Stop | ID |
|---|---|---|---|---|---|---|
| 337.000 | 337.000 | 0.91 | DP | -160.00 | -160.00 | FA22:1_SC |
| | | | CE | -25.00 | -25.00 | |
| | | | CXP | -16.00 | -16.00 | |

| Q1 Mass (Da) | Q3 Mass (Da) | Time (min) | Param | Start | Stop | ID |
|---|---|---|---|---|---|---|
| 367.000 | 367.000 | 0.91 | DP | -160.00 | -160.00 | FA24:0_SC |
| | | | CE | -23.00 | -23.00 | |
| | | | CXP | -16.00 | -16.00 | |

| Q1 Mass (Da) | Q3 Mass (Da) | Time (min) | Param | Start | Stop | ID |
|---|---|---|---|---|---|---|
| 365.000 | 365.000 | 0.92 | DP | -160.00 | -160.00 | FA24:1_SC |
| | | | CE | -22.00 | -22.00 | |
| | | | CXP | -16.00 | -16.00 | |

| Q1 Mass (Da) | Q3 Mass (Da) | Time (min) | Param | Start | Stop | ID |
|---|---|---|---|---|---|---|
| 465.200 | 80.000 | 9.59 | DP | -180.00 | -180.00 | Chol_SO4 |
| | | | CE | -150.00 | -150.00 | |
| | | | CXP | -15.00 | -15.00 | |

**Protocol: siRNA-mediated knockdown**

SMARTpool siRNAs (mixture of 4 siRNA), siRNA targeting ELOVL4, and negative control siRNA were obtained from Dharmacon$^{TM}$-Horizon Discovery. HACaT and N/TERT cells (approximately $2\times10^5$) were seeded into 12-well plates. After overnight incubation, cells were transfected with 20 pmol of SMARTpool siRNAs using Lipofectamine RNAiMax Reagent (Thermofisher Scientific) as per manufacturer's instructions.

**Protocol: Culture and stimulation of primary human keratinocyte cell lines**

Primary human keratinocytes isolated from healthy controls (n=50) were cultured in 12- well plates until they reached 70% confluence. Medium was 154CF, Kit (Catalog number: M154CF500) supplemented with HKGS (Catalog number: S001K) and 0.1mM calcium. Keratinocyte cultures were then stimulated in parallel for 12h with IL-17A (10ng/ml), TNF (10ng/ml), or IFNg (5ng/ml). At time of harvest cells were barely confluent.
